# Six domoic acid related compounds from the red alga, *Chondria armata*, and domoic acid biosynthesis by the diatom, *Pseudo-nitzschia multiseries*

**DOI:** 10.1038/s41598-017-18651-w

**Published:** 2018-01-10

**Authors:** Yukari Maeno, Yuichi Kotaki, Ryuta Terada, Yuko Cho, Keiichi Konoki, Mari Yotsu-Yamashita

**Affiliations:** 1 Graduate School of Agricultural Science, Tohoku University, 468-1 Aramaki-Aza-Aoba, Aoba-ku, Sendai, 980-0845 Japan; 2 Fukushima College, 1-1 Chigoike Miyashiro, Fukushima, 960-0181 Japan; 3 United Graduate School of Agricultural Sciences, Kagoshima University, 1-21-24, Korimoto, Kagoshima, 890-0065 Japan

## Abstract

Domoic acid (DA, **1**), a potent neurotoxin that causes amnesic shellfish poisoning, has been found in diatoms and red algae. While biosynthetic pathway towards DA from geranyl diphosphate and l-glutamate has been previously proposed, its late stage is still unclear. Here, six novel DA related compounds, 7′-methyl-isodomoic acid A (**2**) and B (**3**), *N*-geranyl-l-glutamic acid (**4**), 7′-hydroxymethyl-isodomoic acid A (**5**) and B (**6**), and *N*-geranyl-3(*R*)-hydroxy-l-glutamic acid (**7**), were isolated from the red alga, *Chondria armata*, and their structures were determined. The compounds **4** and **7**, linear compounds, are predictable as the precursors to form the DA pyrrolidine ring. The compounds **2** and **3** are thought as the cyclized products of **7**; therefore, dehydration and electron transfer from the internal olefin of **7** is a possible mechanism for the pyrrolidine ring formation. One terminal methyl group of the side chain of **2** and **3** is predicted to be oxidized to hydroxymethyl (**5**, **6**), and then to carboxylic acids, forming isodomoic acids A and B. Finally, the terminal olefin of isodomoic acid A would be isomerized to form DA. In addition, [^15^N, D]-labeled **4** was incorporated into DA using the diatom, *Pseudo-nitzschia multiseries*, demonstrating that **4** is the genuine precursor of DA.

## Introduction

**Figure 1:**
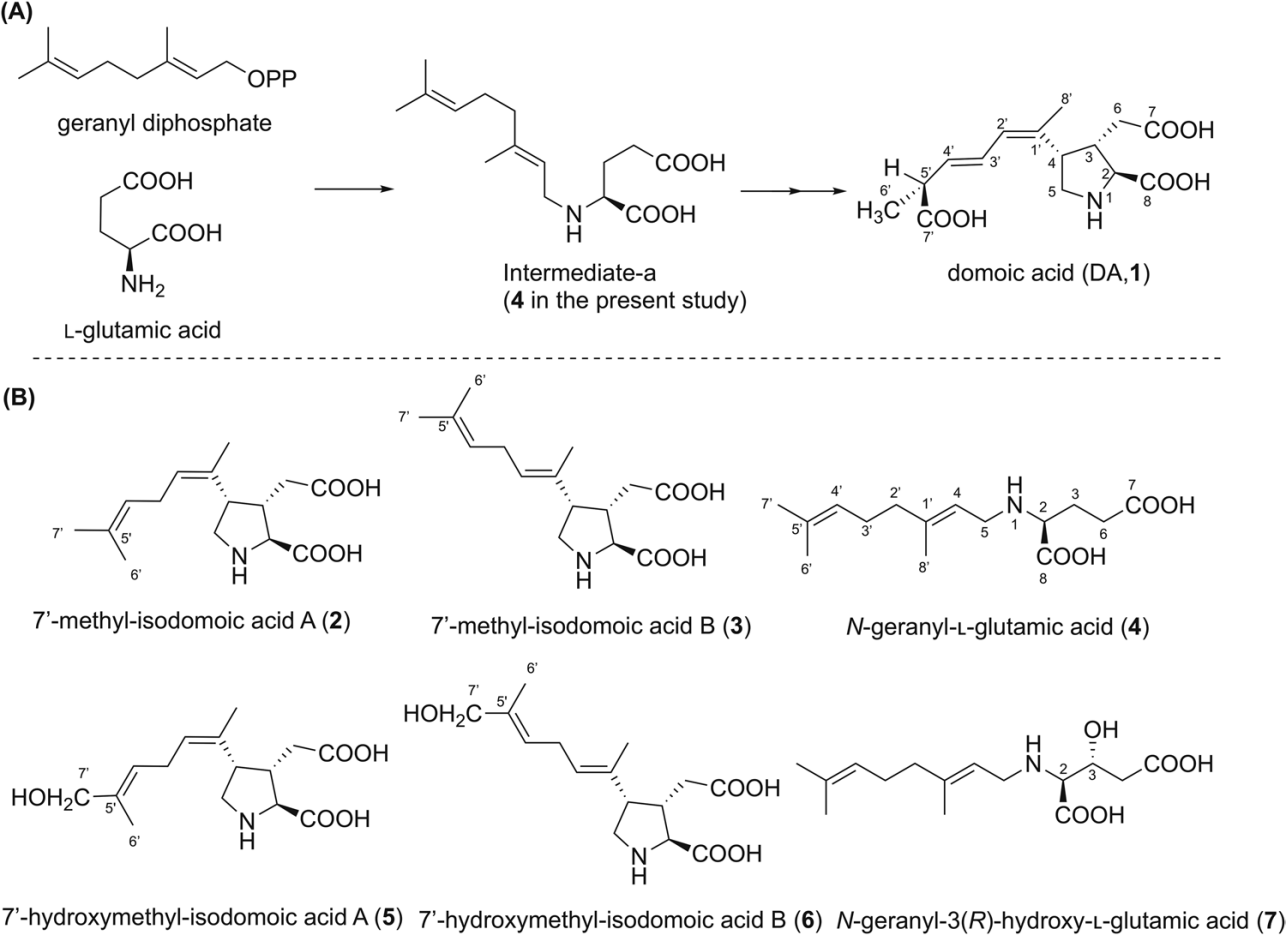
Biosynthetic pathway to DA (**1**) proposed by Savage *et al.* ^23^ (**A**), and the structures of **2**–**7** isolated from the red alga, *C. armata* in this study (**B**).

Domoic acid (DA, **1**, Fig. 1), a neuroexcitatory amino acid, is the toxic principle of amnesic shellfish poisoning (ASP) that first occurred in Prince Edward Island, Canada, in 1987^1,2^. DA was originally isolated from the marine red alga, *Chondria armata* ^3^. In the incidence in Canada, this substance was detected in the diatom *Pseudo-nitzschia multiseries* as well as in the shellfish; therefore, this diatom species was identified as a causative organism of the poisoning^4^. Extensive efforts have been devoted to screen the DA-producing pennate diatoms in recent years and several species of the genus *Pseudo-nitzschia* have been reported to produce DA. In particular, *P. australis* is known for its high toxicity and is responsible for the deaths of sea birds (e.g., pelicans and cormorants) and sea lions, which consumed DA-contaminated anchovies^5,6^. Recent work also revealed that DA impairs sea lion memory and hippocampal connectivity^7^, and that the spread of DA contamination in coastal regions worldwide is related to warm ocean conditions^8^.

DA is a member of kainoids, a group of neurologically active amino acids that include another marine metabolite, kainic acid^7,9^. These compounds possess a common structural feature resembling a conformationally restricted form of l-glutamate, and act as potent agonists of ionic glutamate receptors (iGluRs) by binding to kainate receptors (iGluR5-7, KA1, KA2)^10–12^ in central nervous system. The structures of ligand binding core of the kainate receptors, iGluR5^12^ and iGluR6^13^, have been reported as the complexes with DA.

DA has also attracted the attention of chemists, even though it is a small molecule, due to its characteristic structure that is composed of three carboxylic acids, a pyrrolidine ring, and a (*Z*, *E*)-conjugated diene side chain. The originally proposed structure of DA^3^ was revised by the first total synthesis by Ofune and Tomita^14^. As DA analogues, isodomoic acids A, B, and C (IA, IB, and IC, respectively)^15^, domoilactone A and B^16^ and isodomoic acids G and H^17^ were also isolated from *C. armata*. Small amounts of isodomoic acids A, B^18^, and C^19^ have been identified in DA-producing diatoms, and isodomoic acid D, E, F, and 5-*epi*-domoic acid have been isolated from DA-contaminated shellfish^20^. So far, eleven DA derivatives have been identified in these natural sources^18^.

An early study of the biosynthetic pathway to DA was performed by Wright’s group by examining the labeling patterns of DA produced in *P. multiseries* cultured with ^13^C-acetate. The result suggested that DA is likely produced from the condensation of a C_10_ isoprenoid such as geranyl diphosphate with a C_5_ product of the TCA cycle, α-ketoglutarate^21,22^.

They also proposed a plausible condensed product, an imine intermediate^22^, and subsequent intramolecular cyclization that forms the pyrrolidine ring, although they did not clearly identify that the nitrogen source of DA is l-glutamic acid. Then, Savage *et al.* ^23^ proposed that DA is formed by the condensation of l-glutamic acid and geranyl diphosphate by the nucleophilic displacement reaction based on the experimental incorporation of [1-^2^H_2_]geraniol into DA using *Pseudo-nitzschia* spp. They predicted the intermediate-a in Fig. 1A to be the product of this condensation. However, to the best of our knowledge, no report has been published that experimentally explains the mechanism of cyclization that forms the pyrrolidine ring in DA and the oxidation of its side chain.

Here, we found six new possible biosynthetic intermediates of DA (**2**–**7)** (Fig. 1B) in the red alga, *C. armata*, and their structures were determined by spectroscopic methods and synthesis. We also proposed a DA biosynthetic scheme, especially for the process of cyclization that forms the pyrrolidine ring and the oxidation of the side chain, based on the structures of **2**–**7**. In addition, [^15^N, D]-labeled **4** was incorporated into DA using the diatom, *Pseudo-nitzschia multiseries*, demonstrating that **4** is the genuine precursor.

## Results and Discussion

The lyophilized extract from the red alga, *C. armata* collected in Kagoshima prefecture, Japan, was desalted using a reversed phase column, and then, used for screening of new DA-related compounds having the molecular formula C_15_H_x_NO_y_ using high-resolution (HR)-LC-MS (see methods). At least six unknown peaks were detected: the compounds corresponding to two peaks detected at [M + H]^+^ *m/z* 282.1700 (C_15_H_24_NO_4_^+^) (**2**, **3**), one peak detected at *m/z* 284.1856 (C_15_H_26_NO_4_^+^) (**4**), two peaks detected at *m/z* 298.1649 (C_15_H_24_NO_5_^+^) (**5**, **6**), and one peak detected at *m/z* 300.1805 (C_15_H_26_NO_5_^+^) (**7**) **(**Figs S1–S6). Compounds **2**–**7** were purified by sequential column chromatography. The approximate yields of **2**, **3**, **4**, **5**, **6**, and **7** from 343 g (wet weight) of *C. armata* were 110, 294, 20, 41, 25 and 117 μg, respectively. The structural characterization of these compounds was mainly performed through NMR techniques; ^1^H-^1^H COSY, TOCSY, HSQC, HMBC and NOESY1D spectra were measured for **3**, **5** and **7**, while only ^1^H-^1^H COSY, TOCSY and NOESY1D spectra were measured for **2** and **6**, and only ^1^H-^1^H COSY and TOCSY spectra were measured for **4**, because of the low yields.

**Figure 2:**
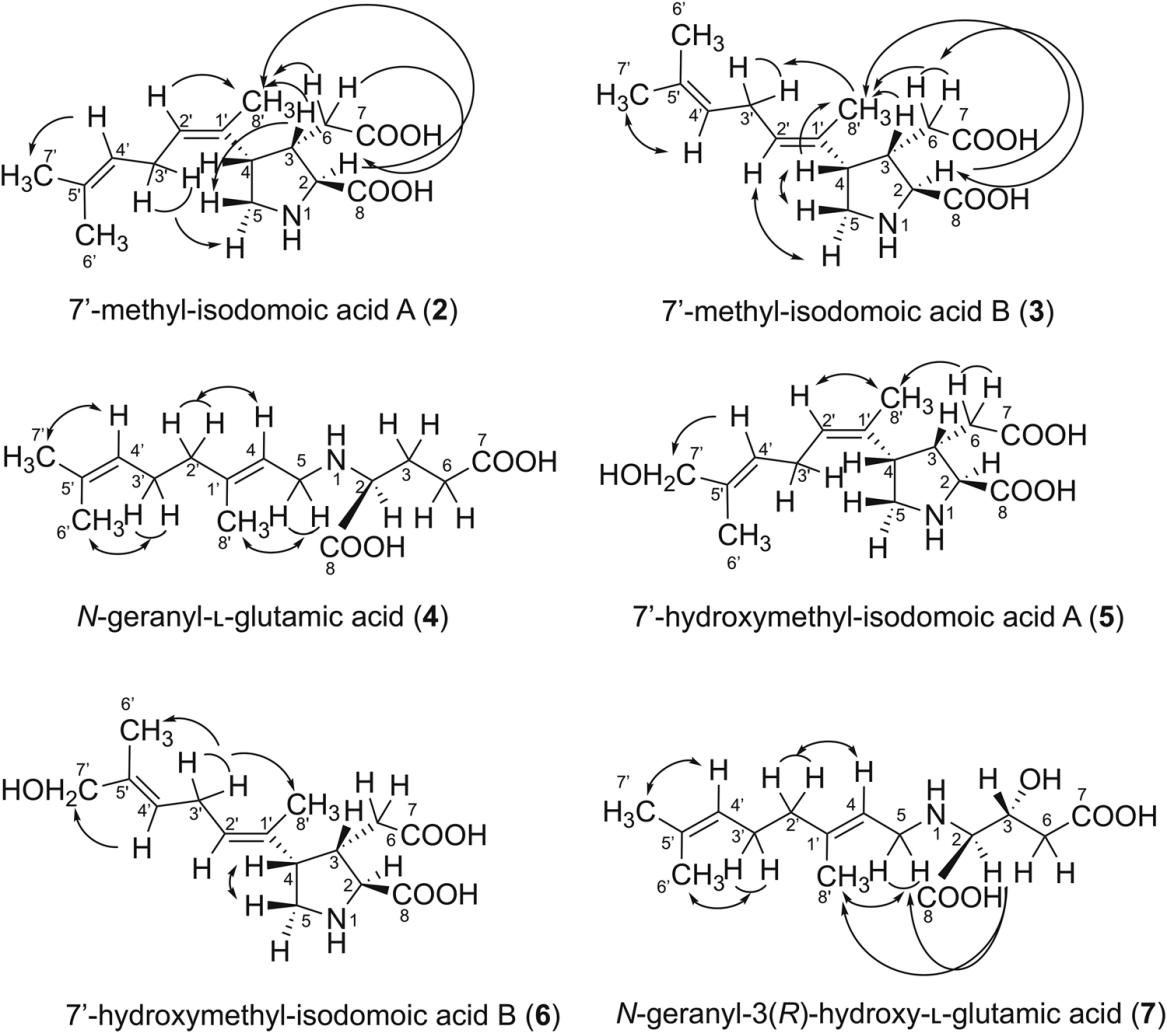
The key NOEs observed in **2**–**7**.

The compounds **2** and **3** have the same molecular formula C_15_H_23_NO_4_ ([M + H]^+^ **2**: *m/z* 282.1697, **3**: *m/z* 282.1701, calcd for C_15_H_24_NO_4_^+^ 282.1700; Figs S1, S2). The analysis of 2D NMR results suggested that **2** and **3** have the same trisubstituted pyrrolidine ring as that of DA and its substituents at C2 (COOH) and C3 (CH_2_-COOH). The side chain structures at C4 of **2** and **3** were suggested to be different from that of DA, both having an isoprene unit (dimethyl allyl group) at the terminus that is not conjugated with other methyl substituted double bond by the coupling patterns indicated in the ^1^H-^1^H COSY and TOCSY spectra including the long-range couplings (H2′/H8, H4′/H6′, H4′/H7′; Figs S8, S9, S24, S25). The connectivity of this carbon chain to C4 was confirmed by HMBC correlations of H2′/C4 and 8′-CH_3_/C4 in **3** (Fig. S27). The above data suggest that **2** and **3** are regioisomers with each other. The geometry of the C1′-C2′ double bond of **2** was assigned as *Z* based on observed NOEs from H2′ to 8′-CH_3_ (Fig. S10), and from 3′-CH_2_ to H5α (Fig. S17) in NOESY1D spectra, while that in **3** was assigned as *E* based on NOEs from 8′-CH_3_ to 3′-CH_2_ (Fig. S37), and between H2′ and H5α (Figs S28, S32) (Fig. 2). The same stereochemistry of C2-C4 in the pyrrolidine ring of **2** and **3** as that of DA was also confirmed by NOEs from H2′ to 8′-CH_3_ (Fig. S10), and from H3 to H5β (Fig. S16) in **2**, and from H2 to 8′-CH_3_ (Fig. S30), and between H2′ and H5α (Figs S28, S32) in **3**, as shown in Fig. 2. This stereochemistry of **3** was also supported by almost agreeing its ^13^C NMR chemical shifts with those of reported IB (**11**)^15^. Based on these data, **2** and **3** were assigned as 7′-methyl-isodomoic acids A and B, respectively (Fig. 1B).

**Figure 3:**
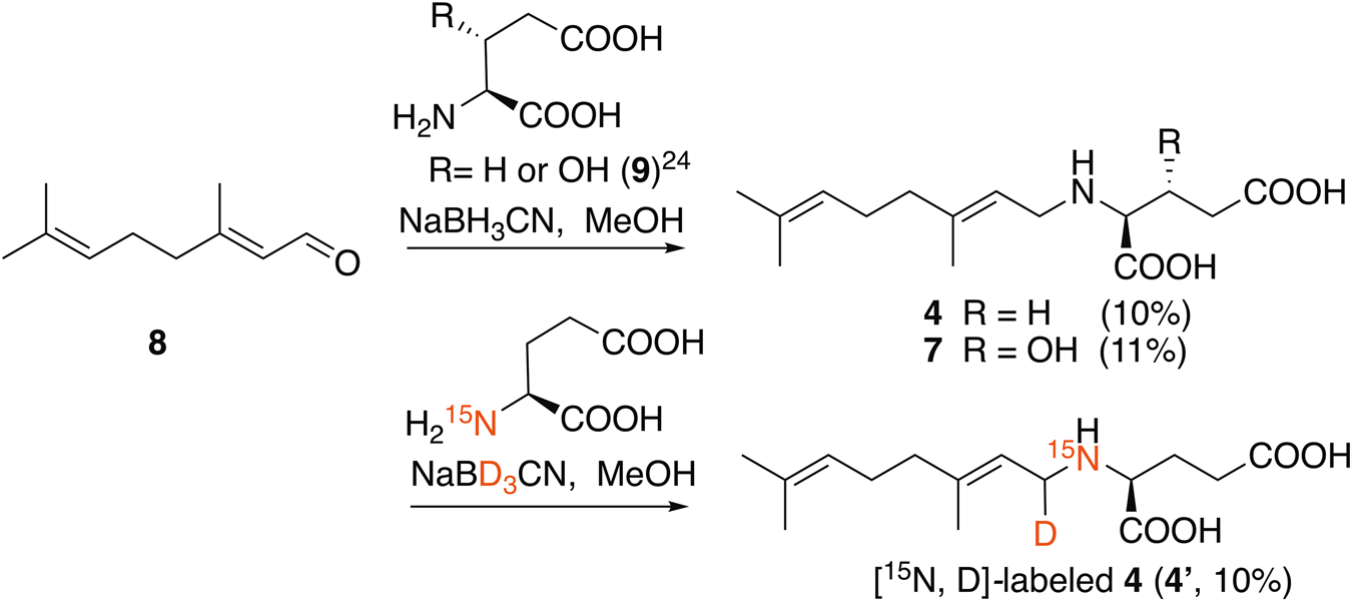
Synthesis of **4**, **7** and **4′**.

The compound **4**, that has the molecular formula C_15_H_25_NO_4_ ([M + H]^+^ **4**: *m/z* 284.1852 calcd for C_15_H_26_NO_4_^+^ 284.1856; Fig. S3), was isolated using several reversed phase columns. This compound was predicted to be identical to the intermediate-a (*N*-geranyl-l-glutamic acid) proposed by Savage *et al.* ^23^ (Fig. 1A) based on its molecular formula and ^1^H NMR,^1^H-^1^H COSY and TOCSY correlations (Figs S39–41). Therefore, **4** was synthesized by reductive amination of l-glutamic acid and geranial (**8**) (Fig. 3) (10% yield). Synthetic **4** was compared with natural **4** from *C. armata* using LC-MS/MS (Fig. S95) and ^1^H NMR spectroscopy (Fig. S93). Almost same retention times and the MS/MS patterns, together with almost identical ^1^H NMR spectra, of the synthetic and natural **4** suggested that **4** is the intermediate-a proposed by Savage *et al*. (Figure 1A)^23^. The chemical shifts of H2 (3.44 ppm) and 6-CH_2_ (2.48 ppm) of natural **4** were up field shifted 0.09 and 0.04 ppm, respectively, from those of synthetic **4**, probably due to the difference of the dissociation rate of 1-NH, 2-COOH, and 7-COOH. The major product ions from the molecular ions of synthetic and natural **4** by MS/MS (Fig. S95) were detected at *m/z* 81.0699 (synthetic) and *m/z* 81.0695 (natural). These product ions were interpreted as possibly being 3-methyl penta-1,3-diene (calcd for C_6_H_9_^+^ 81.0704) corresponding to the partial structure C2′-C7′ of **4**.

The compounds **5** and **6** have the same molecular formula C_15_H_23_NO_5_ ([M + H]^+^ **5**: *m/z* 298.1651, **6**: *m/z* 298.1657, calcd for C_15_H_24_NO_5_^+^ 298.1649; Figs S4, S5), and are suggested to have the same structure as that of DA except the side chain at C4. In the ^1^H NMR spectra of **5** and **6**, two singlet methyl signals at δ 1.65 (6′-CH_3_) and 1.72 (8′ -CH_3_) for **5**, and δ 1.67 (6′-CH_3_) and 1.71 (8′ -CH_3_) for **6**, and one singlet hydroxy methylene group at δ 3.90 (7′-CH_2_) for **5** and 3.91 (7′-CH_2_) for **6** were shown for the side chain at C4 (Figs S42, S59). The connectivities of these protons were determined based on ^1^H-^1^H COSY and TOCSY spectra, suggesting that **5** and **6** are the regional isomers having a hydroxy methyl group at the terminus of the side chain, instead of the methyl group in **2** and **3** (Figs S43, S44, S60, S61). The geometry of two double bonds C1′-C2′ and C4′-C5′ of **5** was assigned as *Z* and *E*, respectively, based on the observed NOEs between H2′ and 8′-CH_3_ (Figs S48, S57), and from H4′ to 7′-CH_2_ (Fig. S49), on the NOESY1D spectra, while those of **6** were assigned as *E* and *E*, respectively, based on NOEs from 3′-CH_2_ to 8′-CH_3_ and 6′-CH_3_ (Fig. S66), and from H4′ to 7′-CH_2_ (Fig. S62; Fig. 2). Concerning the stereochemistry of the pyrrolidine ring of **5**, the *cis* geometry of the substituents at C3 and C4 was indicated by NOE from 6b-CH_2_ to 8′-CH_3_ (Fig. S56). Although other NOEs around the pyrrolidine rings of **5** and **6** were not clearly detected, probably due to the small sample amount, the stereochemistry of the pyrrolidine rings of **5** and **6** was assumed to be identical to that of DA, based on the almost identical chemical shifts of ^1^H of H2-H5 of **6** and **3** (Δ < 0.05 ppm; Table 1), and those of ^13^C of C2-C6 of **5** and DA (Δ < 0.7 ppm; Table 2). These data suggested that **5** and **6** are 7′-hydroxymethyl-isodomoic acids A and B, respectively (Fig. 1B).

**Table 1:** ^1^H NMR spectroscopic data of natural **1**–**7** ^a^. δ_H_ in ppm, multiplicity, and *J* in Hz in parentheses.

	DA (**1**)^25^	**2**	**3**	**4**	**5**	**6**	**7**
2	3.98, d (8.1)	3.85, d (7.6)	3.94, d (4.4)	3.44, m	3.87, d (6.8)	3.98, d (6.8)	3.46, d (6.7)
3	3.05, dddd (9.1, 8.4, 5.8)	2.98, m	3.06, m	2.04, m	2.99, m	3.08, m	4.31, m
4	3.84, ddd (7.9, 7.3)	3.33, m	2.97, ddd	5.31, t (7.3)	3.62, m	2.95, m	5.29, t (7.6)
5α	3.49, dd (12.2, 7.3)	3.56, dd (11.7, 8.2)	3.30, m	3.65, dd (13.8, 7.6)	3.34, m	3.34, m	3.69, d (7.6)
5β	3.71, dd (12.2, 7.9)	3.61, dd (15.4, 7.3)	3.47, dd (11.7, 7.3)	3.58, dd (14.1, 6.8)	3.56 m	3.47, m	3.69, d (7.6)
6a	2.76, dd (16.7, 5.8)	2.68, dd (16.7, 5.9)	2.37, dd (16.6, 6.9)	2.49, m	2.66, m	2.28, m	2.82, dd (16.3, 3.7)
6b	2.50, dd (16.7, 9.1)	2.42, dd (16.7, 8.8)	2.31, dd (16.6, 7.2)	2.49, m	2.41, dd (16.6, 8.4)	2.28, m	2.60, dd (16.1, 7.9)
2′	6.13, d (11.1)	5.38, t (7.3)	5.09, t (7.1)	2.10, t (7.3)	5.42, t (7.3)	5.16, t (6.5)	2.11, m
3′a	6.35, dd (14.9, 11.1)	2.73, m	2.75, t (7.0)	2.14 dt (13.2, 7.3)	2.80, m	2.81, t (6.8)	2.14, m
3′b	6.35, dd (14.9, 11.1)	2.61, m	2.75, t (7.0)	2.14 dt (13.2, 7.3)	2.69, m	2.81, t (6.8)	2.14, m
4′	5.78, dd (14.9, 7.8)	5.04, t (7.3)	5.14, t (7.0)	5.10, t (6.4)	5.32, t (6.8)	5.38, t (6.8)	5.10, t (6.3)
5′	3.30, dq (7.8, 7.1)	—	—	—	—	—	—
6′	1.27, d (7.1)	1.61, s	1.63, s	1.61, s	1.65, s	1.67, s	1.61, s
7′	—	1.67, s	1.68, s	1.67, s	3.90, s	3.91, s	1.67, s
8′	1.81, s	1.71, s	1.69, s	1.75, s	1.72, s	1.71, s	1.73, s

^a^Spectra were measured at 600 MHz in CD_3_OD.

**Table 2:** ^13^C NMR spectroscopic data of **1**, **3**, **4**, **5** and **7** ^a^. δ_C_ in ppm.

	DA (**1**)^25^	**3**		synthetic **4**	**5**		**7**	
	δ_C_	δ_C_^b^	HMBC	δ_C_	δ_C_^b^	HMBC	δ_C_^b^	HMBC
2	67.1	67.5	5β, 6a, 6b,	62.7	66.6	5β, 6b,	66.7	3, 5, 6a, 6b
3	44.6	43.3	2, 4, 5α, 5β, 6a, 6b	27.4	44.4	2, 4, 5β, 6b	68.5	2, 6a, 6b
4	42.7	49.0	5α, 5β, 6a, 6b, 2′, 8′	115.9	42.7	2, 5α, 5β, 6b, 8′	115.8	5, 2′, 8′
5	49.1	47.9	2, 3, 4	46.1	48.4	3	46.0	2, 4
6	35.4	34.8	2, 3, 4	32.2	35.2	2, 3	41.3	2
7	177.5	175.7	3, 6	177.7	176.0	6a, 6b	175.3	3, 6a, 6b
8	174.9	173.4	2, 3	173.6	173.3	2, 3	172.0	2
1′	133.8	131.2	4, 5α, 5β, 3′, 8′	148.3	133.8	3′a, 3′b, 8′	148.2	5, 2′, 8′
2′	132.8	128.7	4, 3′, 8′	41.5	131.8	3′a, 3′b, 4′, 8′	41.3	4, 3′, 8′
3′	128.6	28.5	2′, 4′	28.0	28.0	2′, 4′	27.8	2′, 4′, 5′
4′	135.2	123.8	2′, 3′, 6′, 7′	125.5	124.5	2′, 3′a, 3′b, 6′, 7′	125.2	2′, 3′, 6′, 7′
5′	44.9	133.4	3′, 6′, 7′	133.8	136.8	3′a, 3′b, 6′, 7′	133.5	3′, 6′, 7′
6′	18.6	18.4	4′, 7′	18.6	13.8	4′, 7′	18.5	4′, 7′
7′	23.5	26.4	4′, 6′	26.7	68.9	4′, 6′	26.5	4′, 6′
8′	181.9	17.5	2′, 4	17.5	22.6	2′	17.3	4, 2′

^a^Spectra were measured at 151 MHz in CD_3_OD.

^b^Chemical shifts were roughly determined based on the cross peaks in HMBC spectra.

The compound **7** has the molecular formula C_15_H_25_NO_5_ ([M + H]^+^ *m/z* 300.1813 calcd for C_15_H_26_NO_5_^+^ 300.1805; Fig. S6). The ^1^H-^1^H COSY, TOCSY and HMBC correlations (Figs S71, S72, S74) suggested that **7** has a linear structure similar to that of **4** without the bond between C3 and C4 of DA. Hydroxylation at C3 of **7** was indicated by the presence of the oxymethine signal (H3, δ 4.31, C3, δ 68.5 ppm) and HMBC correlations (C3/H2, C3/6-CH_2_; Fig. S74). NOEs observed between H4 and 2′-CH_2_ (Figs S75, S82), and 5-CH_2_ and 8′-CH_3_ (Figs S78, S83) suggested *E* geometry for C1′-C4 olefin (Fig. 2). For determination of the stereochemistry of C3, a preliminary attempt to prepare the MTPA-derivative of **7** was unsuccessful, so **7** was synthesized by the reductive amination of 3-hydroxy glutamic acid and **8** (Fig. 3, see methods). 3-Hydroxy glutamic acid (*threo* and *erythro* diastereomers mixture) was synthesized by the reported method^24^, and then, *threo*-3-hydroxy glutamic acid (**9**) was separated from its *erythro* diastereomer by column chromatography. Next, **9** was condensed with **8** by reductive amination (Fig. 3) to yield **7** (yield 11%). The NMR spectra of synthetic **7** was identical to that of natural **7** (Fig. S94). Thus, the stereochemistry of **7** was determined to be 2 *S*, 3 *R*, because **7** should be biosynthesized from l-glutamic acid (2 *S*) related compound. Based on these data, **7** was assigned as *N*-geranyl-3(*R*)-hydroxy-l-glutamic acid (Fig. 1B). The assignments of ^1^H NMR signals of natural **2**–**7** and ^13^C NMR signals of **3**, **4** (synthetic), **5**, and **7** are listed in Tables 1 and 2, respectively, with those of **1** ^25^ for comparison.

**Figure 4:**
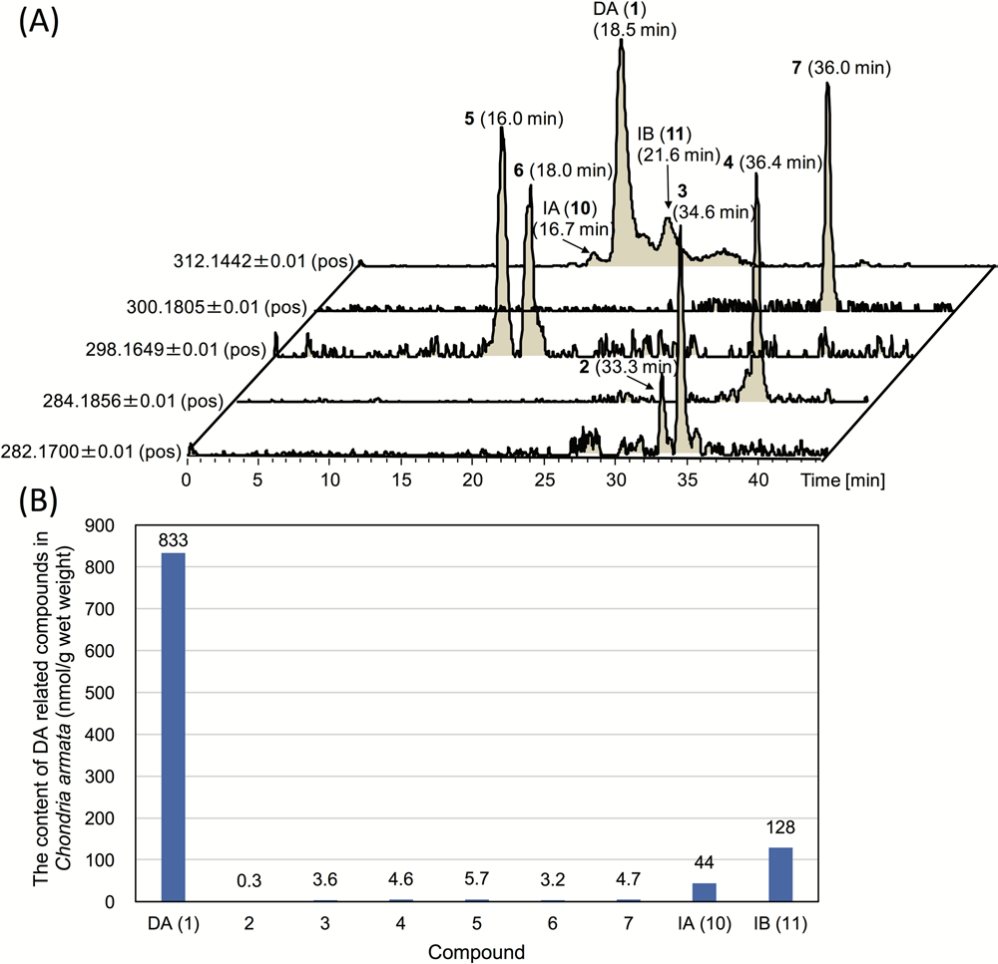
Quantitative analysis results for DA and related compounds in *Chondria armata*. The extracted mass chromatograms of **1**–**7**, **10**, and **11** in the semipurified extract from *C. armata* (see methods) (**A**), and the contents of **1**–**7**, **10**, and **11** in *C. armata* (**B**).

The contents of DA (**1**) and **2**–**7**, **10**, and **11** in *C. armata* were quantified using HR-LC-MS (see methods). The compounds **2** and **3**, and also **5** and **6**, with the same molecular formula were successfully separated from each other using a reversed phase column and gradient elution system (Fig. 4A). The analytical results of quantification of **1–7**, **10**, and **11** in *C. armata* are shown in Fig. 4B. The compounds **2**–**7** were suggested to be minor components compared with **1**, being only 0.03–0.7% (mol/mol) of **1**. Therefore, we assumed that most of the biosynthetic intermediates are eventually converted to **1** in *C. armata*, or excess amounts are excreted out of this alga.

**Figure 5:**
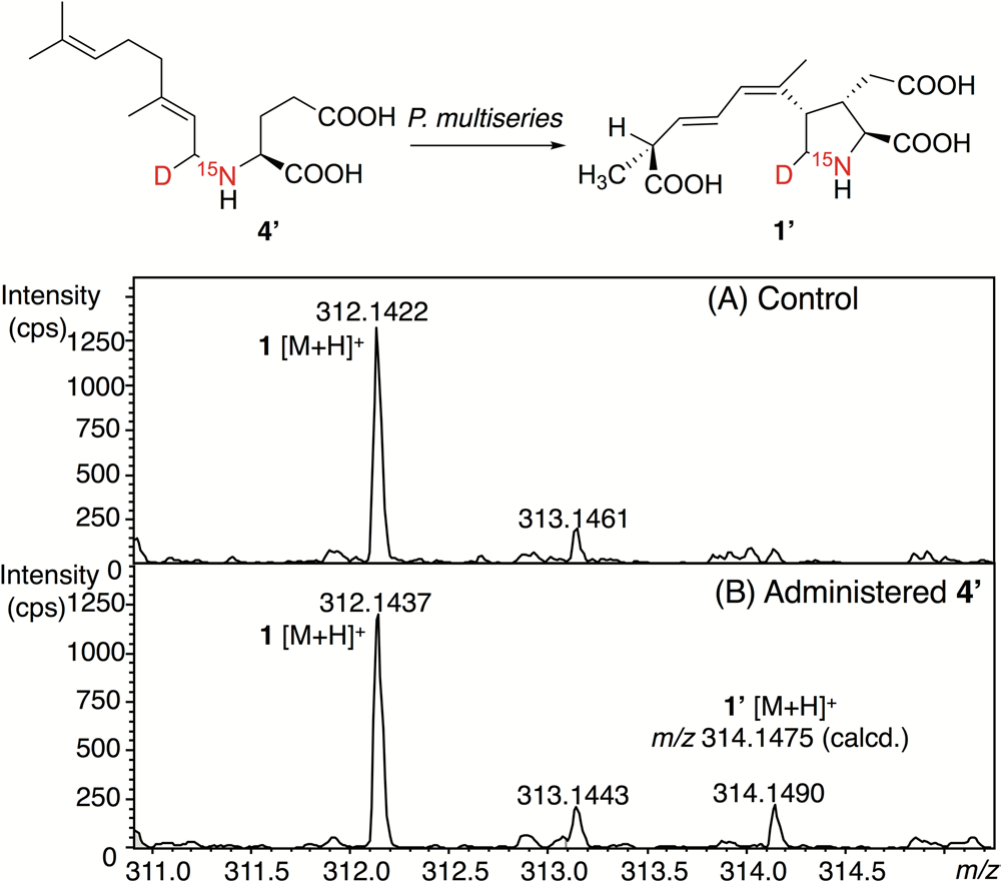
Incorporation of [^15^N, D]-labeled **4** (**4′**) into DA (**1′**) using the diatom, *Pseudo-nitzschia multiseries*, cultured for 17 days. The isotope pattern of DA (**1**) obtained from the culture of control (not administered **4′**) (**A**), and that administered **4′** (**B**).

Next, incorporation of stable isotope labeled **4** (**4**′) into DA was examined using the diatom, *Pseudo-nitzschia multiseries*. The synthetic [^15^N, D]**4** (**4**′) (Fig. 3) was administered at 25 µM to the culture medium at 5 days after inoculation. The culture without **4**′ was similarly prepared as control. DA was semi-purified from an aliquot of each 17-day culture (mixture of cells and medium) and analyzed using HR-LC-MS. The isotope patterns of DA from control (A) and that from the culture administered **4′** (B) are shown in Fig. 5. The theoretical isotope pattern of DA [M + H]^+^ (C_15_H_21_NO_6_) is *m/z* 312.1442 (relative abundance: 100), 313.1474 (17.1), 314.1496 (2.6), and the calculated exact mass of [M + H]^+^ for [^15^N, D]DA (**1′**) (C_15_H_20_D^15^NO_6_) is *m/z* 314.1475. In the isotope pattern of DA from the culture administered **4′** (Fig. 5B), the ion at *m/z* 314.1490 corresponding to **1′** was clearly detected, while that of DA from control almost agreed to the theoretical isotope pattern of DA (Fig. 5A). This result suggested that **4′** was partially incorporated into DA produced by *P. multiseries*, even in the low labeling rate (approximately 14%, based on the intensity of the isotope ions), demonstrating that **4** is the genuine precursor of DA for the first time.

**Figure 6:**
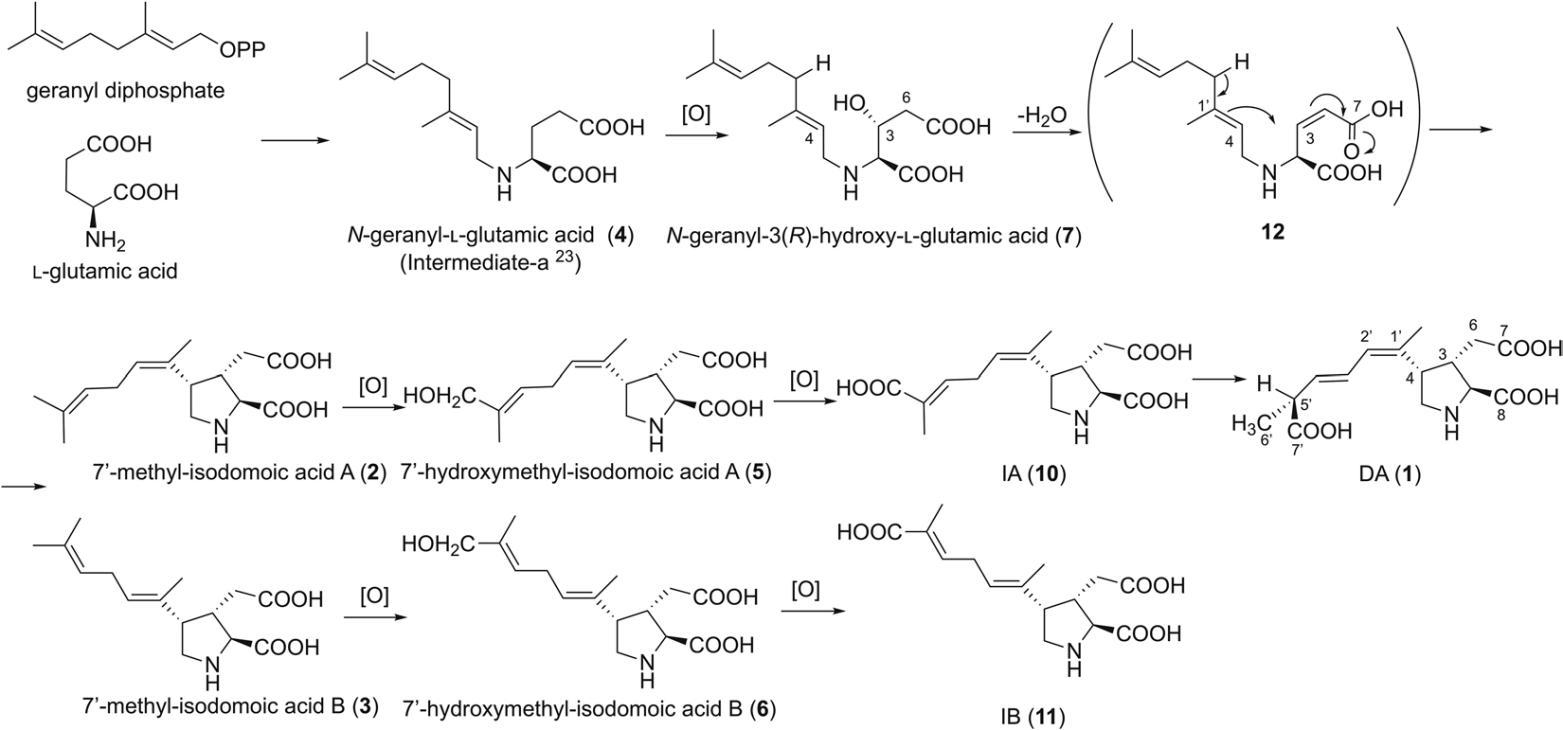
Proposed scheme for biosynthesis of DA (**1**) in the present study.

The biosynthetic pathway to DA is predicted as shown in Fig. 6, based on the structures of newly found **2**–**7** in this study, together with the previously reported IA (**10**) and IB (**11**). The biosynthesis of DA probably starts from the condensation of l-glutamic acid (or its related compound) and geranyl diphosphate to form **4** as previously predicted^21,23^, and then, C3 in **4** would be hydroxylated to generate **7** that can be thought as the adjacent precursor for the formation of the pyrrolidine ring of DA. In this study, **4** was proved to be the precursor of DA by the incorporation of this compound into DA using the diatom. Therefore, the possibility that **7** was produced by condensation of 3-hydroxy glutamic acid and geranyl diphosphate as Garson predicted^26^ can be excluded. For hydroxylation of 3-CH_2_ in **4** to form **7**, nonheme iron(II)-and α-ketoglutarate-dependent hydroxylases are predictable to catalyze such stereospecific hydroxylation of amino acids at the relatively unreactive position with molecular oxygen^27–29^.

Given that the stereochemistry of DA and all its congeners contain C3, C4 *cis* substituents, the cyclization of **7** to **2** and **3** would not proceed in S_N_2 like manner, because of the configuration of 3-hydroxy group in **7**. Instead, this reaction would preferentially proceed in S_N_1 like manner; secondary cation at C3 would be produced by elimination of water, and then, this cation would accept the electron from the double bond (C1′-C4) in the side chain of **7**. This electron transfer from this double bond would produce two geometric isomers **2** and **3** (*Z*/*E*). A similar cyclization reaction has been reported for biosynthesis of the sesquiterpene, botrydial, for example^30^. As another possible cyclization mechanism, intermolecular Michael-type cyclization reaction in **12** (dehydrated product of **7**) can be predicted, while **12** has not yet been identified in natural sources. Another Michael-type cyclization precursor similar to **12** was also proposed by Wright’s group^22^. Michael-type cyclization reactions have been reported in many biosynthetic routes, for example, psiguadinal B^31^. Interestingly, as a possible cyclization precursor of acromelic acid, another kainoid from the poisonous mushroom, *Clitocybe acromelalga*, a structurally related compound to **4** (l-*N*-[2-(3-Pyridyl)ethyl]-glutamic acid) has been reported^32^. This indicates the similar cyclization mechanism for formation of a pyrrolidine ring in DA and acromelic acid.

After the cyclization, one of the terminal methyl groups (C7′) in isoprenoid side chain of **2** and **3** are predicted to be oxidized in a stepwise manner to the hydroxymethyl groups in **5** and **6**, and then, to carboxylic acids in **10** and **11**. Then, DA would be produced from **10** by isomerization of its terminal olefin to form the conjugated diene of DA. For this side chain oxidation, cytochrome P450 mono-oxygenase (P450) is predicted to be involved. The methyl groups in terpenoids are usually oxidized by this class of enzyme, for example, during the biosynthesis of the fungal product, sesterfisheric acid^33^. P450 is commonly present in a wide range of organisms, including plants and marine diatoms^34^.

In conclusion, we isolated six novel possible biosynthetic intermediates of DA (**2**–**7**) from the red alga, *C. armata*, and proposed the biosynthetic pathway to DA, including the cyclization mechanisms for the formation of its pyrrolidine ring and the stepwise oxidation of the side chain at C4. In addition, stable isotope labeled **4** was incorporated into DA using the diatom, *P. multiseries*, demonstrating that **4** is the genuine precursor of DA. The presence of **2**–**7** in *P. multiseries*, and the labeling patterns of these compounds are now being investigated. We expect that the identification of biosynthetic intermediates will contribute to identify DA biosynthetic genes in diatoms that have not yet been identified. Furthermore, the biological activities of **2**–**7** should be examined in future.

## Methods

### General experimental procedures

Standard DA (**1**) was purchased from BioVectra, Inc. (Charlottetown, Canada). The dry solvents for organic synthesis were purchased from Wako Pure Chemical Industries, Ltd. (Osaka, Japan). The other reagents were purchased from Sigma-Aldrich Co. (St. Louis, MO, USA), Wako Pure Chemical Industries, Ltd., Tokyo Chemical Industry Co. Ltd. (Tokyo, Japan), Nacalai Tesque, Inc. (Kyoto, Japan), and Kyowa Hakko Bio. (Tokyo, Japan). LC-MS-grade acetonitrile, formic acid (Wako Pure Chemical Industries, Ltd.) and MeOH (Kanto Chemical, Tokyo, Japan) were used for HR-LC-MS. Distilled and purified water (MilliQ) by Simplicity UV (Merck Millipore Corporation, Billeraca, MA, USA) was used for all the experiments. LC-MS was performed with a micrOTOF-Q II mass spectrometer (HR, ESI, Q-TOF; Bruker Daltonics, Billerica, MA, USA) and API2000 (ESI, triple quadrupole; AB SCIEX, Foster City, CA, USA). HRMS was measured with a micrOTOF-Q II mass spectrometer. NMR spectra were mainly measured on an Agilent 600 MHz NMR spectrometer (Agilent Technologies, Santa Clara, CA, USA) with 5 mm id probe in 0.4 mL of CD_3_OD (deuteration degree 99.95%) at 20 °C. HSQC and HMBC (^3^ *J*_CH_ = 8 Hz) spectra of **5** were measured in 0.5 mL of CD_3_OD on a Bruker AVANCE III 600 (Bruker, Billerica, MA, USA) with 5 mm CryoProbe. Spectra were referenced to residual solvent signals with resonances at δ H/C = 3.30/49.8 ppm (CD_3_OD) and at δ N = 0 ppm (NH_3_). Optical rotation was measured on a P-2200 (Jasco corporation, Hachioji, Japan).

### Plant materials and diatom

The red alga, *Chondria armata*, was collected by snorkeling at Hanasezaki, Ibusuki, Kagoshima Prefecture, Japan, in July and August 2013, at a depth of approximately 1 m during the low tide, and identified by R.T. (one of the authors). The diatom, *Pseudo-nitzschia multiseries*, isolated from Ofunato bay, Iwate Prefecture, Japan, in August 2014, was identified according to Hasle^35^, and Hasle and Lundholm^36^, based on morphological observation using SEM.

### Screening of predicted biosynthetic intermediates using HR-LC-MS

The marine red alga, *C. armata* (800 g) was extracted with boiling water (1.5 L). The extract was lyophilized (35 g) and kept at −80 °C. For the screening, a part of this lyophilized sample (0.15 g) was homogenized with MeOH (3 mL) and centrifuged for 5 min at 12,000 *g* at 4 °C. The supernatant was centrifuged again and filtrated through a Cosmospin Filter H (0.45 µm, Nacalai Tesque) by centrifugation for 5 min at 12,000 *g* at 4 °C. The filtrate was loaded on a Cosmosil 140C_18_-OPN column (0.5 mL, Nacalai Tesque) pre-equilibrated with H_2_O-HCOOH (100:0.1, v/v). After the column was washed with the same solvent, the three solvents, H_2_O-MeOH-HCOOH (80:20:0.1, 50:50:0.1, and 20:80:0.1, v/v/v, 1.5 mL each), were supplied in a stepwise manner to the resin and each eluate was collected. An aliquot of each eluate was subjected to HR-LC-MS using a micrOTOF-Q II mass spectrometer. LC was performed using a Mightysil RP-18GP column (2.0 × 150 mm, 5 µm, Kanto Chemical) with H_2_O-MeOH-HCOOH (50:50:0.1, v/v/v) as the mobile phase at a flow rate of 0.15 mL/min at 28 °C, using two LC-30AD pumps (Shimadzu, Kyoto, Japan), a CTO-20AC column oven (Shimadzu), a SIL-30AC autosampler (Shimadzu), and a CBM-20A communications bus module (Shimadzu). Acquisition parameters of the mass spectrometer were as follows: ion polarity: positive, capillary: 4500 V, nebulizer: 1.6 bar, dry heater: 180 °C, dry gas: 7.0 L/min (N_2_). The extracted ion chromatograms (EICs) of the predicted molecular formula for biosynthetic intermediates of DA were analyzed with Smart Formula^TM^ software (Bruker Daltonics).

### Purification of 2–7

A part of the lyophilized extract (5 g) from *C. armata* (see above) was homogenized with MeOH (150 mL) and centrifuged for 5 min at 12,000 *g* at 4 °C. The solvent was evaporated from the supernatant under vacuum, and the residue was dissolved in H_2_O-MeOH-HCOOH (40:60:0.1, v/v/v, 4 mL). The solution was filtrated through a Cosmospin Filter H (0.45 µm) by centrifugation as described above. The filtrate was diluted with 40 mL of H_2_O and loaded onto a Cosmosil 140C_18_-OPN column (56 mL) pre-equilibrated with H_2_O-HCOOH (100:0.1, v/v). The compounds were eluted in a stepwise manner with H_2_O-HCOOH (100:0.1, v/v), H_2_O-MeOH-HCOOH (80:20:0.1, v/v/v, **1**, **5**, **6**), H_2_O-MeOH-HCOOH (50:50:0.1, v/v/v, **2**, **3**, **4** and **7**), and MeOH-HCOOH (100:0.1, v/v) each 150 mL. The flow rate was 3 mL/min. First, the eluate containing **2**, **3**, **4**, and **7** (H_2_O-MeOH-HCOOH, 50:50:0.1, v/v/v) was concentrated and applied to an InertSustain C18 column (7.6 × 250 mm, 5 µm, GL Sciences, Tokyo, Japan) with H_2_O-MeOH-HCOOH (40:60:0.1, v/v/v). The compounds **2** and **3** were eluted as a mixture first, and then, **4** and **7** were eluted. The compounds **4** and **7** were obtained in the almost pure form by further purification using an InertSustain AQ C18 column (7.6 × 250 mm, 5 µm, GL Sciences, Tokyo, Japan) with H_2_O-MeOH-HCOOH (50:50:0.1, v/v/v). The mixture of **2** and **3** were separated with each other using an InertSustain C18 column (4.6 × 250 mm, 5 µm) with H_2_O-MeOH-HCOOH (50:50:0.1, v/v/v) and obtained in the almost pure form. The above eluate containing **5** and **6** was also concentrated and applied to an InertSustain C18 column (7.6 × 250 mm, 5 µm) with H_2_O-MeOH-HCOOH (85:15:0.1, v/v/v). As a result, almost pure **5** was obtained, while **6** was further purified using a Mightysil RP-18GP column (4.6 × 250 mm, 5 µm) with H_2_O-MeOH-HCOOH (85:15:0.1, v/v/v). Eluted **2**–**7** were analyzed by routine LC-MS (see SI). Eventually, almost pure **2**, **3**, **4**, **5**, **6**, and **7** (100, 294, 20, 41, 25, and 117 µg, respectively) were obtained from *C. armata* (343 g wet weight).

### Synthesis of 4 and [^15^N, D]-labeled 4 (4′)

To a solution of l-glutamic acid (10 mg, 0.068 mmol; Kyowa Hakko Bio) in 500 µL of MeOH, citral (**8′**; mixture of geranial (**8**) and neral, approx. 1:1 mol/mol, 40 µL, 0.23 mmol; Tokyo Chemical Industry) was added. After stirring for 3 h at room temperature (25 °C), NaBH_3_CN (2 mg, 0.032 mmol) was added to the mixture that was continuously stirred for 4 h at 0 °C. The dried reaction mixture with a N_2_ stream was dissolved in H_2_O-HCOOH (100:0.1, v/v, 0.1 mL), and then loaded onto a Cosmosil 140C_18_-OPN column (0.5 mL) pre-equilibrated with H_2_O-HCOOH (100:0.1, v/v). The column was washed with H_2_O-HCOOH (100:0.1, v/v, 1.5 mL) and then with H_2_O-MeOH-HCOOH (80:20:0.1, v/v/v, 1.5 mL). Crude **4** eluted with H_2_O-MeOH-HCOOH (50:50:0.1, v/v/v, 1.5 mL) was purified using an InertSustain C18 column (7.6 × 250 mm, 5 µm) with H_2_O-MeOH-HCOOH (55:45:0.1, v/v/v). Synthetic **4** (1.0 mg, 0.0035 mmol, yield 10%); [α]^20^_D_ = 5.78 (c = 0.00225, MeOH), HRESIMS [M + H]^+^ *m/z* 284.1848 (calcd for C_15_H_26_NO_4_^+^ 284.1856). NMR data are in Tables 1 and 2. [^15^N, D]*N*-geranyl-l-glutamic acid (**4′**) was synthesized using [^15^N]l-glutamic acid (20 mg, 0.14 mmol, Taiyo Nippon Sanso, Tokyo, Japan), **8′** (80 µL, 0.47 mmol) and NaBD_3_CN (12 mg, 0.18 mmol, Sigma-Aldrich) by the same method as that for **4**, and similarly purified. **4′** (2.0 mg, 0.007 mmol, yield 10%), HRESIMS [M + H]^+^ *m/z* 286.1886 (calcd for C_15_H_25_^15^NDO_4_^+^ 286.1889). NMR data are in SI.

### Synthesis of 7 (racemic)

*Threo*-3-hydroxy glutamic acid (racemic; **9**) was synthesized from dimethyl 3-oxoglutarate (100 mg, 0.575 mmol) according to the reported method^24^, and obtained as a mixture of *erythro* and *threo* diastereomers (**9′**, 2 mg, 0.012 mmol). Before reaction, **9** was purified as described in SI. The stereochemistry of obtained **9** (1 mg, 0.0061 mmol) was identified by ^1^H NMR by comparison with the reported data^24,37^. For the reaction, **9** (1 mg, 0.0061 mmol) was dissolved in 450 µL of MeOH and then **8′** (3 µL, 0.0176 mmol) was added to this solution. The mixture was stirred for 3 h at room temperature (25 °C), and then NaBH_3_CN (2 mg, 0.032 mmol) was added. After stirring for 4 h at 0 °C, the solvent was evaporated using a N_2_ stream. The crude **7** was dissolved in H_2_O-HCOOH (100:0.1, v/v, 0.1 mL) and loaded on a Cosmosil 140C_18_OPN column (0.5 mL) pre-equilibrated with H_2_O-HCOOH (100:0.1, v/v). After washing the column with the same solvent (0.5 mL) and H_2_O-MeOH-HCOOH (80:20:0.1, v/v/v, 1.5 mL), the products including **7** were eluted with H_2_O-MeOH-HCOOH (50:50:0.1, v/v/v, 1.5 mL). Finally, HPLC separation of **7** from the condensed product of **9** with neral using an InertSustain C18 column (7.6 × 250 mm, 5 µm) with H_2_O-MeOH-HCOOH (55:45:0.1, v/v/v) gave pure **7** (racemic, 100 µg, 0.00033 mmol, yield 11%). Synthetic 300 (**7**): HRESIMS [M + H]^+^ *m/z* 300.1805 (calcd for C_15_H_26_NO_5_^+^ 300.1805). NMR data are in SI.

### Quantitative analysis of 1–7, 10 and 11 in *C. armata* by LC-MS

The lyophilized extract (0.19 g) of *C. armata* (4.3 g wet weight) with hot water (see above) was homogenized with 3 mL of MeOH and then centrifuged for 5 min at 12,000 *g* at 4 °C. The supernatant (1.6 mL) was filtered through a Cosmospin Filter H (0.45 µm) by centrifugation for 5 min at 12,000 *g* at 4 °C. A part of the filtrate (2/5, v/v) was concentrated to 0.15 mL under vacuum, and loaded on a Cosmosil 140C_18_OPN column (0.3 mL) pre-equilibrated with H_2_O-HCOOH (100:0.1, v/v). After the column was washed with the same solution, DA-related compounds were gradually eluted by increasing the concentration of MeOH (H_2_O-MeOH-HCOOH 80:20:0.1, 50:50:0.1, 20:80:0.1, v/v/v, 1.5 mL for each). An aliquot of each fraction (30, 15, and 60 µL) were diluted with H_2_O-MeOH-HCOOH (50:50:0.1, v/v/v, 70, 85, and 40 µL, respectively), and then 1 µL of each solution was subjected to HR-LC-MS three times. The contents of **1**–**7**, **10** and **11** were estimated as the total in these three fractions. Purchased **1** was used as the standard of **1**. Synthetic **4** was used as the standard for **2**, **3**, **4**, **5**, and **6**, and synthetic **7** was used as the standard for **7**. The standards of **10** and **11** were prepared by one of the author (Y.K.)^38^. HR-LC-MS was performed on micrOTOF-Q II using a Mightysil RP-18GP column (2.0 × 150 mm, 5 µm) at 25 °C with the gradient elution. Mobile phase A was H_2_O-MeOH-HCOOH (80:20:0.1, v/v/v) and mobile phase B was H_2_O-MeOH-HCOOH (10:90:0.1, v/v/v). A gradient elution program was applied as follows: 0–20 min 0% B, 20–25 min 0–40% B, 25–45 min 40% B. The flow rate was 0.2 mL/min.

### Incorporation of [^15^N, D]*N*-geranyl-l-glutamic acid (4′) into DA using *Pseudo-nitzschia multiseries*

*P. multiseries* was inoculated to f/2 medium (70 mL) in a 250 mL tissue culture flask (Falcon, product#353136, Corning, NY, USA) to set the initial concentration of cells at 2,200 cells/mL, and cultured at 15 °C under light intensity of 120–130 µmol photon/m^2^/s with 12:12 light:dark cycle. To the 5 days culture, DMSO solution of **4′** was administered to be the final concentrations of **4′** and DMSO at 25 µM and 0.14%, respectively. The same culture without **4′** was used as control. An aliquot of the 17 days culture (5 mL, **4′** administered culture: 17,400 cell/mL, **4′** not administered culture: 22,000 cells/mL) was taken and sonicated on ice for 1 min, and then concentrated under vacuum. The residue was dissolved in 0.4 mL of H_2_O-HCOOH (100:0.1, v/v) and 0.4 mL of MeOH-H_2_O-HCOOH (30:70:0.1, v/v/v) to be filtered through Cosmospin Filter H. The filtrate was applied to a Cosmosil 140C_18_OPN column (0.5 mL) equilibrated with H_2_O-HCOOH (100:0.1, v/v). After washing the column with H_2_O-HCOOH (100:0.1, v/v, 2 mL), DA was eluted with H_2_O-MeOH-HCOOH (70:30:0.1, v/v/v, 1.5 mL). An aliquot of this elution (2 µL) was applied to HR-LC-MS with a Mightysil RP-18GP column (2.0 × 150 mm, 5 µm) and H_2_O-MeOH-HCOOH (70:30:0.1, v/v/v) at 25 °C. The flow rate was 0.2 mL/min.

## Electronic supplementary material

Supplementary Information Part1 (1/2)

Supplementary Information Part2 (2/2)
