# Supplementary material for: Six domoic acid related compounds from the red alga, Chondria armata, and domoic acid biosynthesis by the diatom, Pseudo-nitzschia multiseries
Source: Sci Rep. 2018 Jan 10;8:356. doi: 10.1038/s41598-017-18651-w (PMC5762911; doi:10.1038/s41598-017-18651-w)
Supplement: Supplementary file 1 — Supplementary Information Part1 (1/2) [file 41598_2017_18651_MOESM1_ESM.pdf]

Supplementary Information   Part 1 (1/2): page 1-30 (Figures S1-S58)

Six domoic acid related compounds from the red alga, *Chondria armata,* and domoic acid biosynthesis by the diatom, *Pseudo-nitzschia multiseries*

Yukari Maeno[1], Yuichi Kotaki[2], Ryuta Terada[3], Yuko Cho[1], Keiichi Konoki[1] and Mari Yotsu-Yamashita[1,*]

[1]Graduate School of Agricultural Science, Tohoku University, 468-1 Aramaki-Aza-Aoba, Aoba-ku, Sendai 980-0845, Japan

[2]Fukushima College, 1-1 Chigoike Miyashiro, Fukushima 960-0181, Japan

[3]United Graduate School of Agricultural Science, Kagoshima University, 1-21-24, Korimoto, Kagoshima 890-0065, Japan

Table of Contents

1. HR-ESI-MS spectra of natural **2**-**7**.

・7'-methyl-isodomoic acid A (**2**) : *m/z* 282.1697 $[M+H]^+$ (calcd for $C_{15}H_{24}NO_4$ 282.1700)

・7'-methyl-isodomoic acid B (**3**) : *m/z* 282.1701 $[M+H]^+$ (calcd for $C_{15}H_{24}NO_4$ 282.1700)

・*N*-geranyl-L-glutamic acid (**4**) : *m/z* 284.1852 $[M+H]^+$ (calcd for $C_{15}H_{26}NO_4$ 284.1856)

・7'-hydroxymethyl-isodomoic acid A (**5**) : *m/z* 298.1651 $[M+H]^+$ (calcd for $C_{15}H_{24}NO_5$ 298.1649)

・7'-hydroxymethyl-isodomoic acid B (**6**) : *m/z* 282.1657 $[M+H]^+$ (calcd for $C_{15}H_{24}NO_5$ 282.1649)

・*N*-geranyl-3(*R*)-hydroxy-L-glutamic acid (**7**) : *m/z* 300.1813 $[M+H]^+$ (calcd for $C_{15}H_{26}NO_5$ 300.1805)

2. NMR spectra of natural **2**-**7**.

・7'-methyl-isodomoic acid A (**2**) : ^{1}H NMR, COSY, TOCSY, NOESY1D

・7'-methyl-isodomoic acid B (**3**) : ^{1}H NMR, COSY, TOCSY, HSQC, HMBC, NOESY1D

・*N*-geranyl-L-glutamic acid (**4**) : ^{1}H NMR, COSY, TOCSY

・7'-hydroxymethyl-isodomoic acid A (**5**) : ^{1}H NMR, COSY, TOCSY, HSQC, HMBC, NOESY1D

・7'-hydroxymethyl-isodomoic acid B (**6**) : ^{1}H NMR, COSY, TOCSY, NOESY1D

・*N*-geranyl-3(*R*)-hydroxy-L-glutamic acid (**7**) : ^{1}H NMR, COSY, TOCSY, HSQC, HMBC, NOESY1D

3. NMR spectra of synthetic **4** and **7**.

・Synthetic *N*-geranyl-L-glutamic acid (**4**) : ^{1}H NMR, ^{13}C NMR, NOESY1D

・Synthetic *N*-geranyl-3(*R*)-hydroxy-L-glutamic acid (**7**) : ^{1}H NMR, HSQC, HMBC

・Comparison of ^{1}H NMR spectra between natural **4** and synthetic **4**

・Comparison of ^{1}H NMR spectra between natural **7** and synthetic **7**

・NMR data of synthetic **4** and **7**.

4. HR-LC-MS/MS spectra of natural **4** and synthetic **4**.

5. HR-ESI-MS spectra and NMR spectra of [^{15}N, D]*N*-geranyl-L-glutamic acid (**4'**).

・[^{15}N, D]*N*-geranyl-L-glutamic acid (**4'**) : *m/z* 286.1886 $[M+H]^+$ (calcd for $C_{15}H_{25}D^{15}NO_4$ 286. 1889)

・[^{15}N, D]*N*-geranyl-L-glutamic acid (**4'**) : ^{1}H NMR, ^{13}C NMR, ^{15}N-^{1}H HMBC

6. Synthesis and purification of **9**.

7. LC-MS for routine detection of **1**-**7**.

References

1. HR-ESI-MS spectra of 2-7.

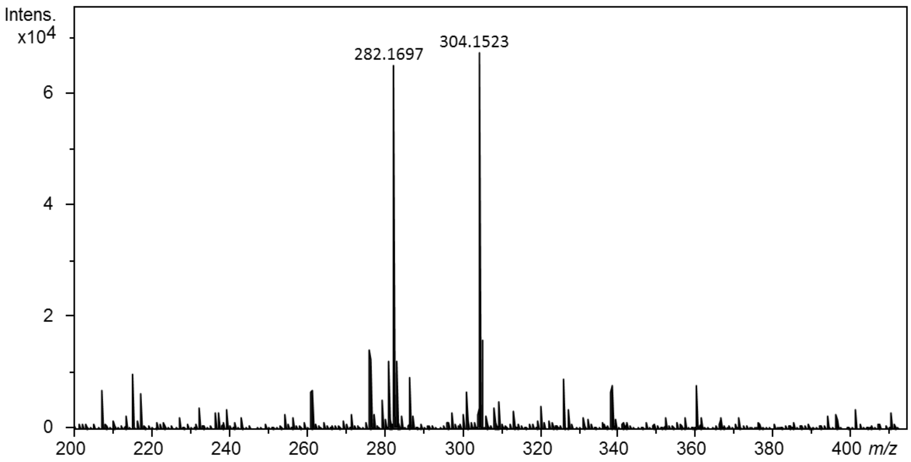

Figure S1. HR-ESI-TOF mass spectrum of **2**.

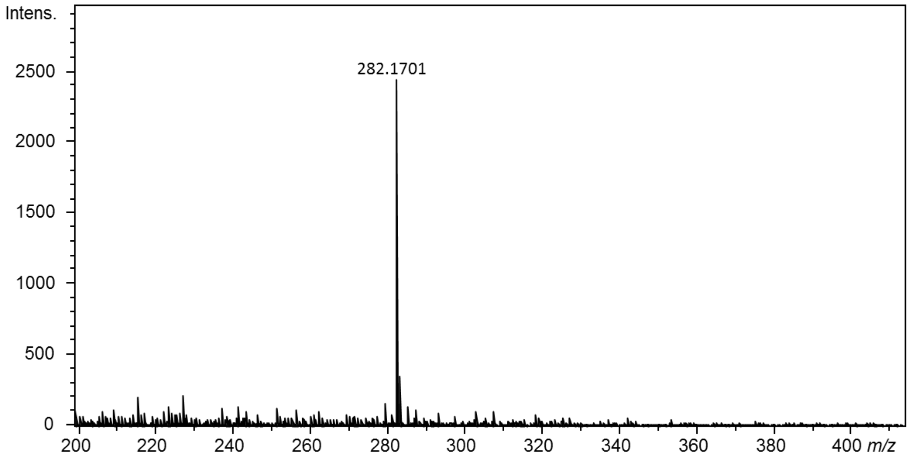

Figure S2. HR-ESI-TOF mass spectrum of **3**.

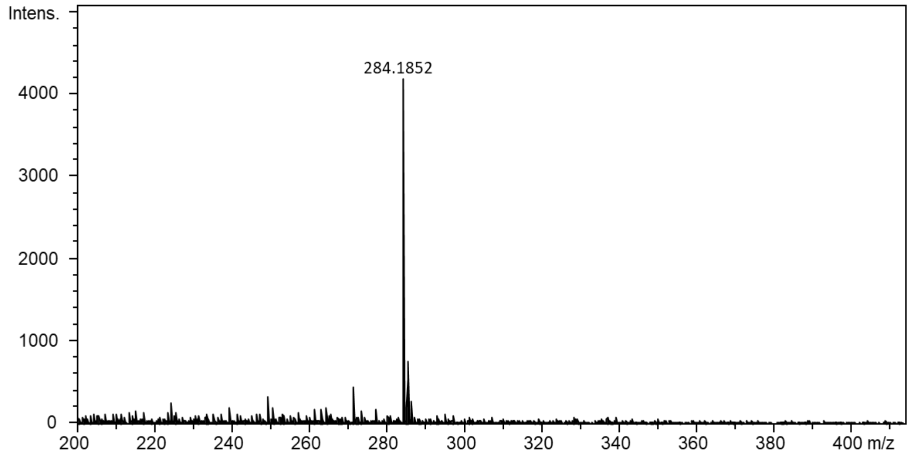

Figure S3. HR-ESI-TOF mass spectrum of **4**.

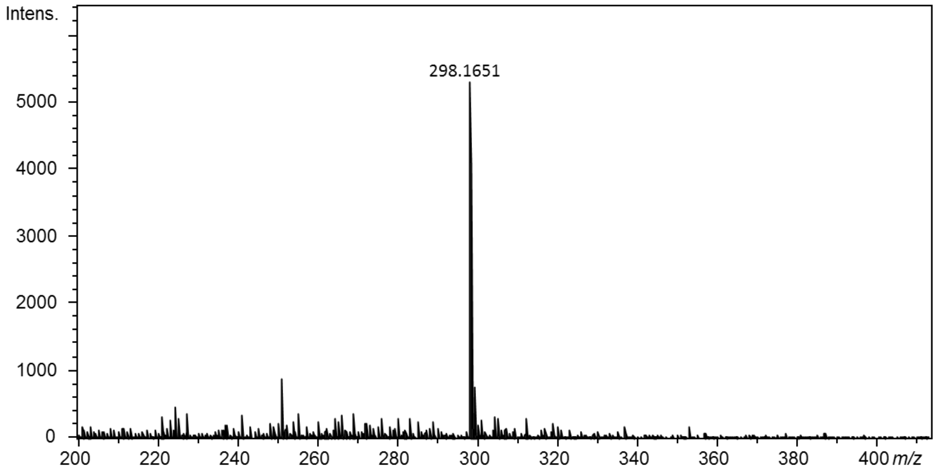

Figure S4. HR-ESI-TOF mass spectrum of **5**.

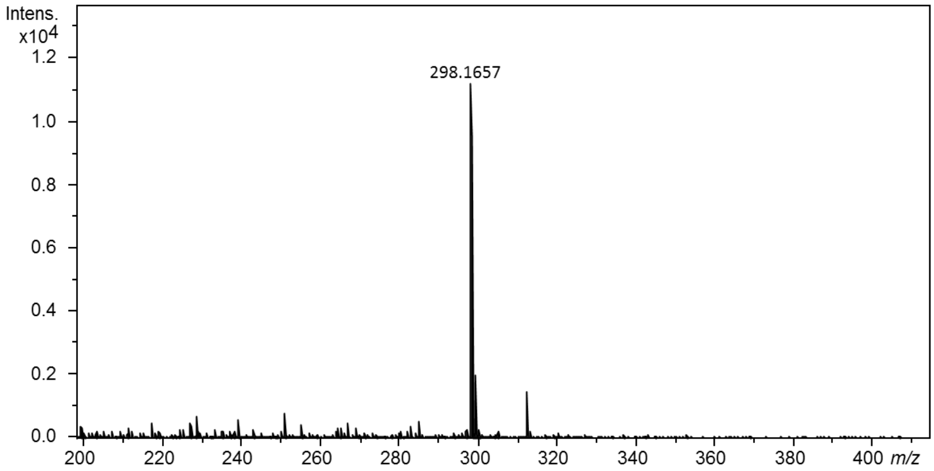

Figure S5. HR-ESI-TOF mass spectrum of **6**.

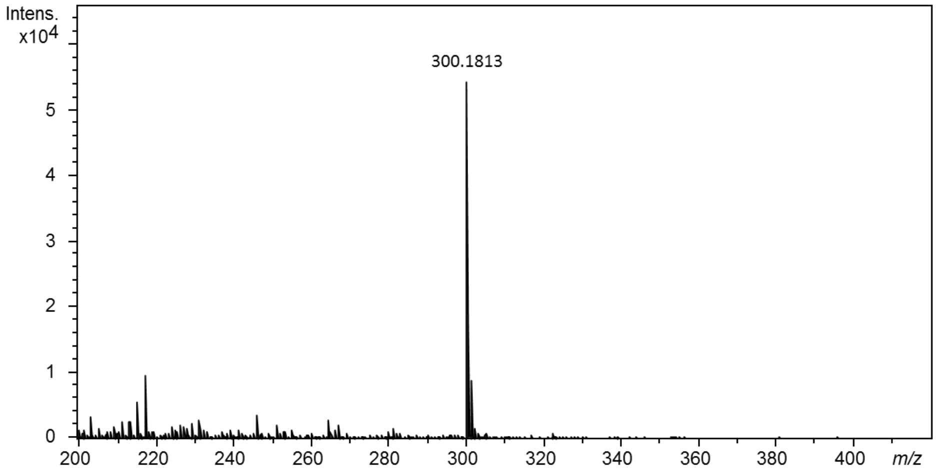

Figure S6. HR-ESI-TOF mass spectrum of **7**.

2. NMR spectra of natural 2-7.

・NMR spectra of 7’-methyl-isodomoic acid A (**2**).

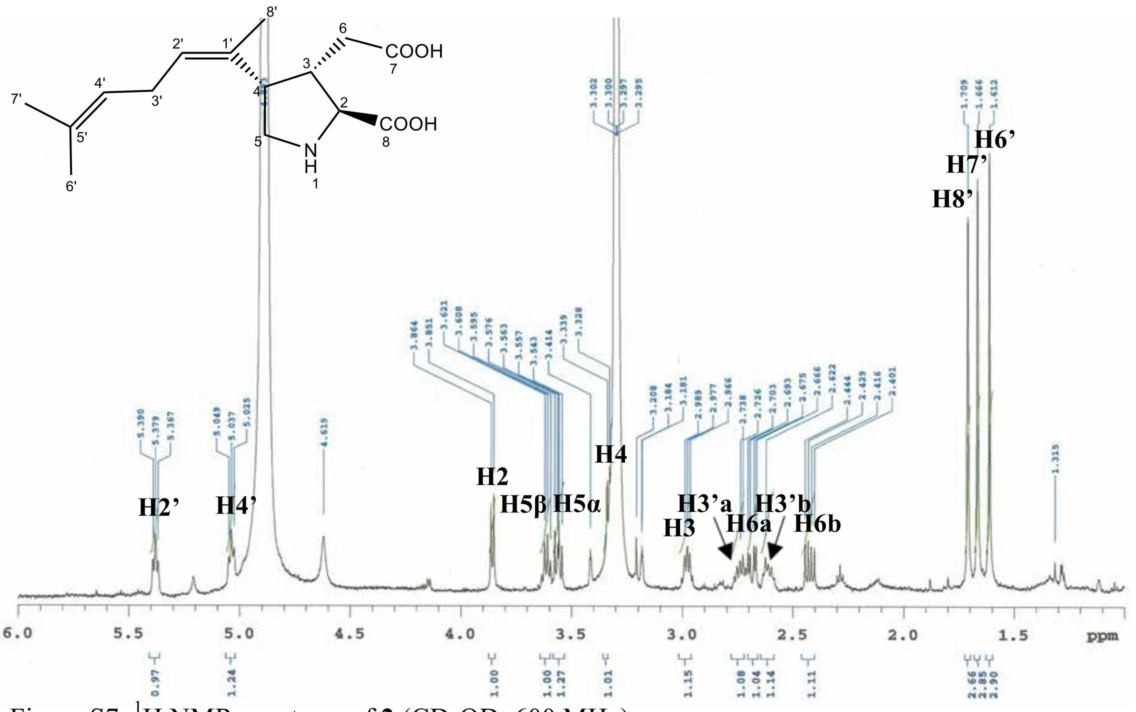

Figure S7. ^{1}H NMR spectrum of **2** (CD_3OD, 600 MHz).

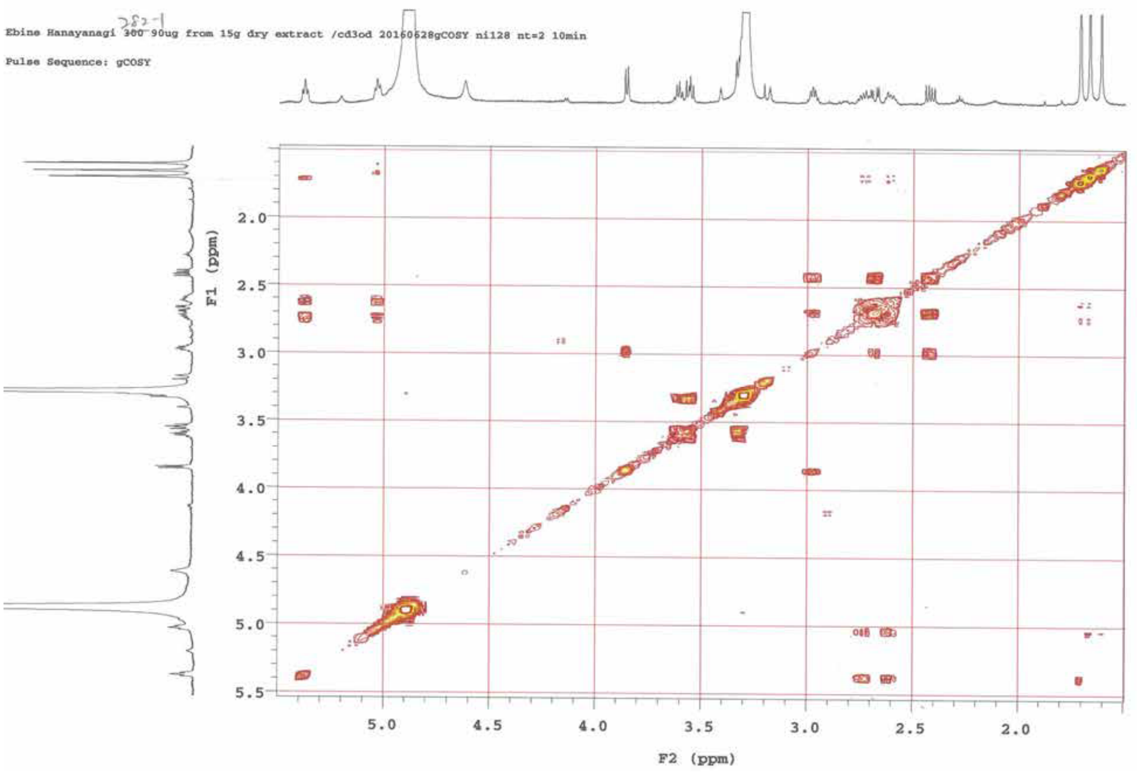

Figure S8. Gradient COSY spectrum of **2** (CD_3OD, 600 MHz).

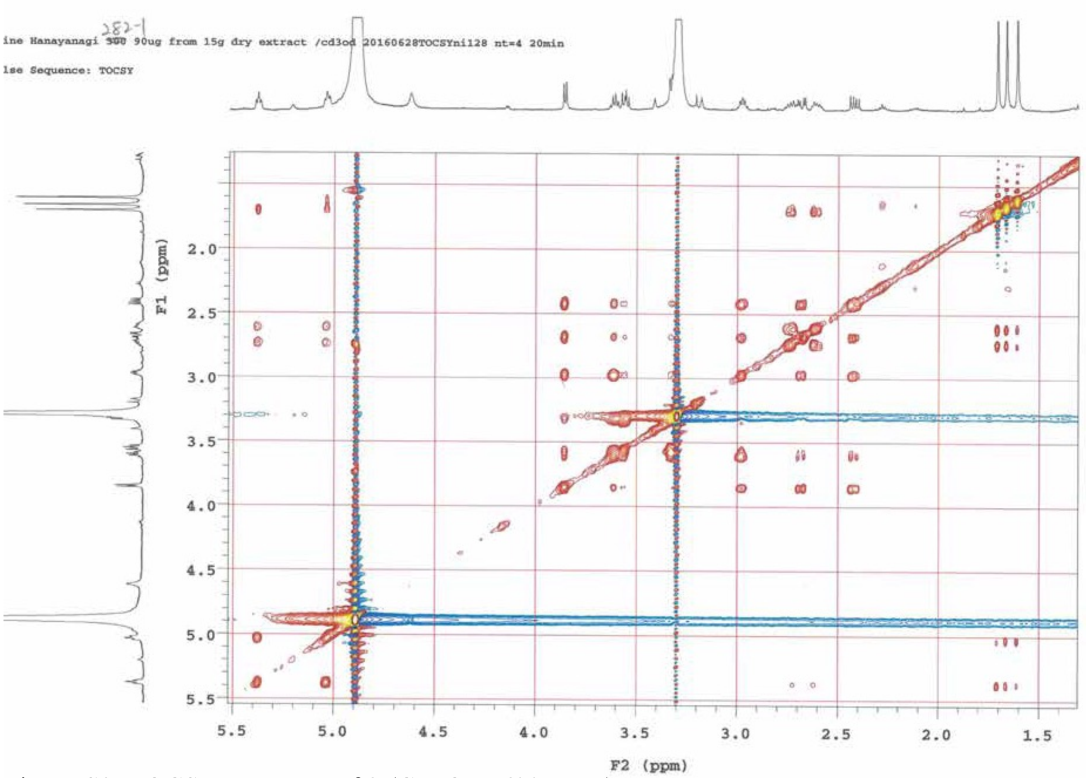

Figure S9. TOCSY spectrum of **2** (CD_3OD, 600 MHz).

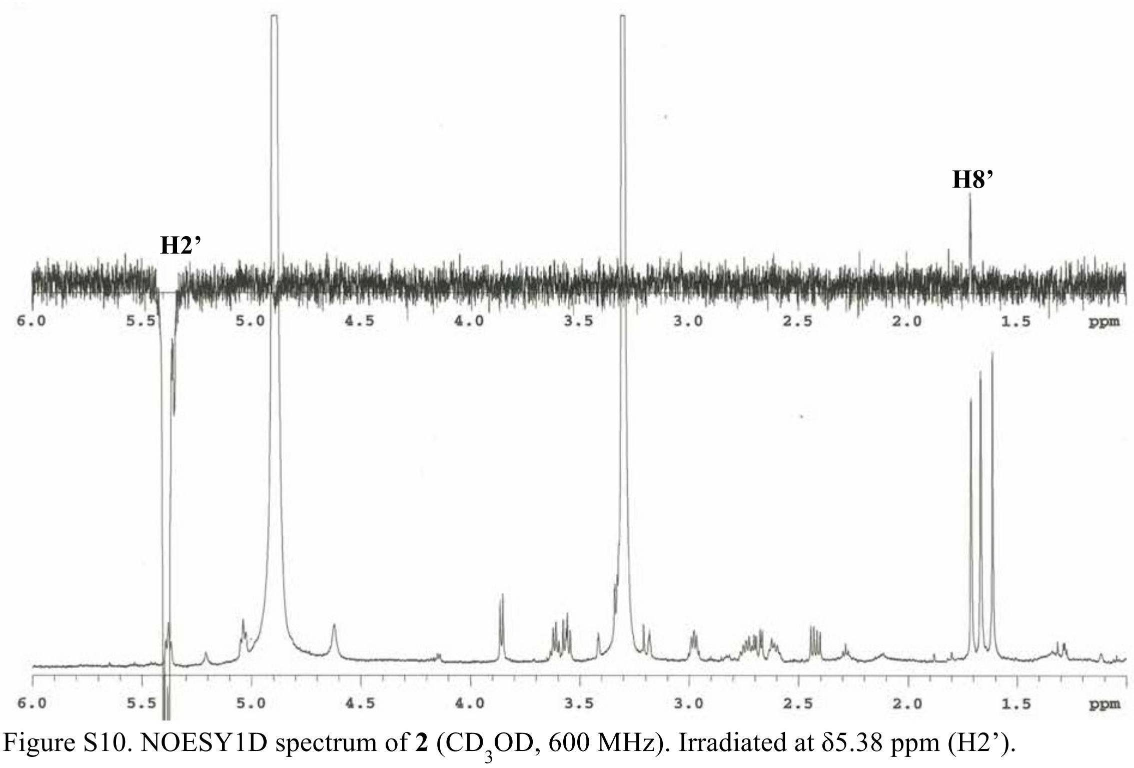

Figure S10. NOESY1D spectrum of **2** (CD_3OD, 600 MHz). Irradiated at δ5.38 ppm (H2’).

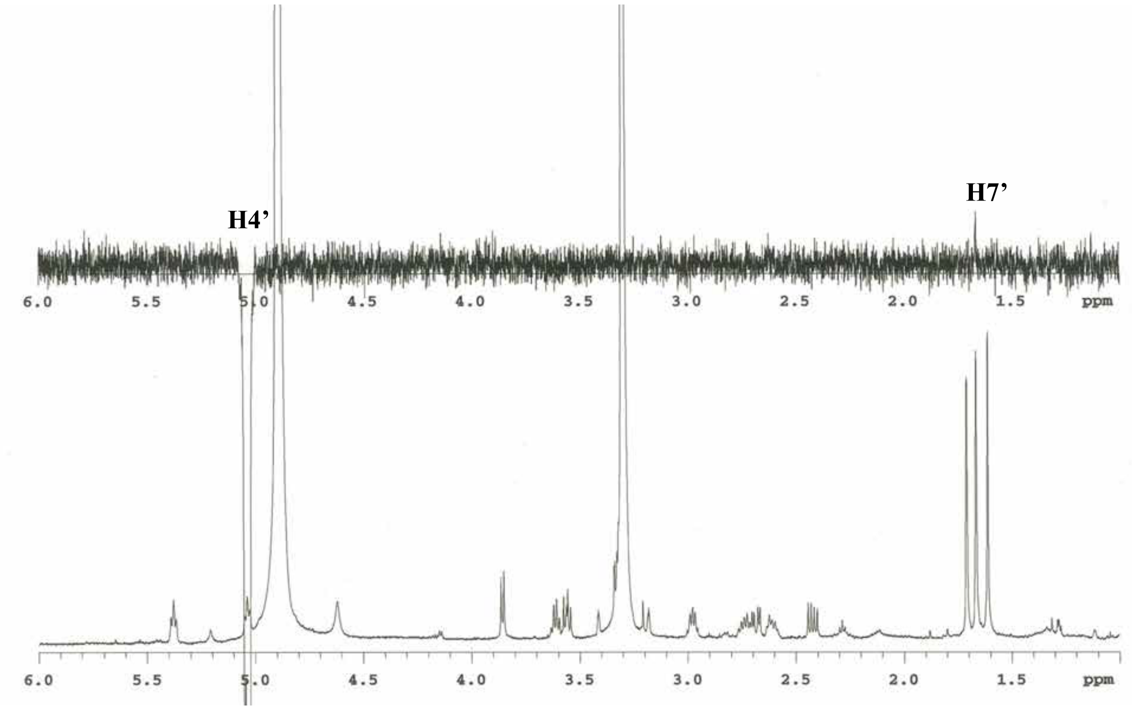

Figure S11. NOESY1D spectrum of **2** (CD_3OD, 600 MHz). Irradiated at δ5.04 ppm (H4’).

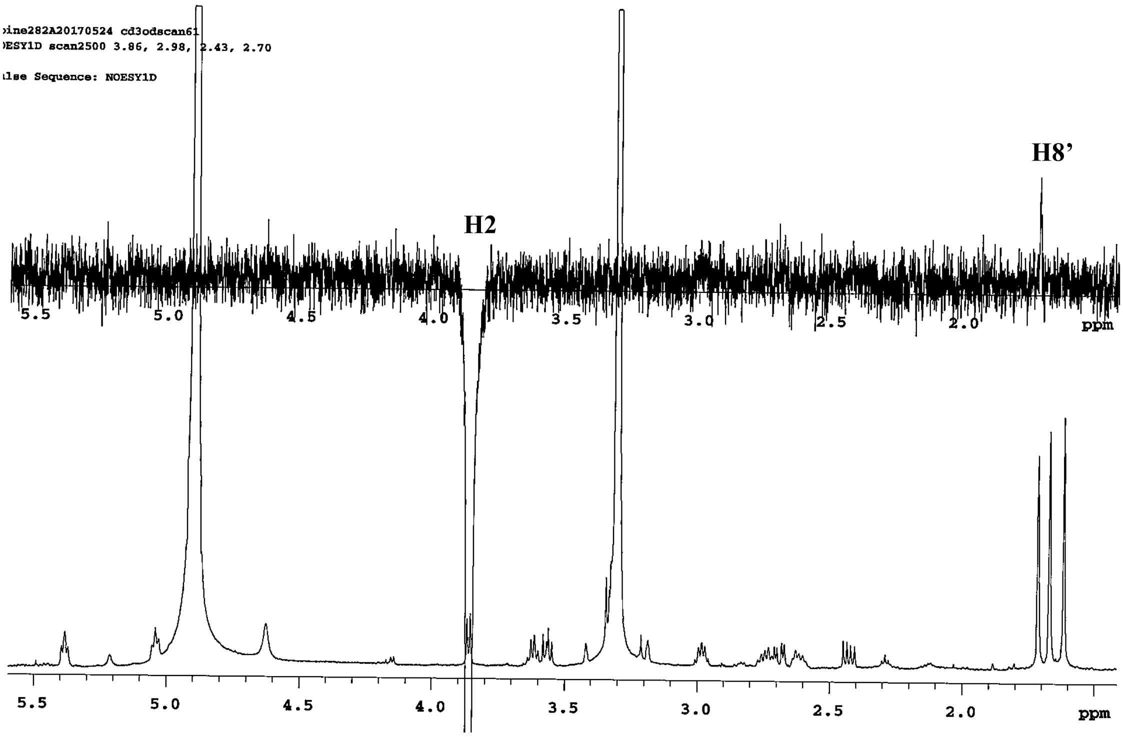

Figure S12. NOESY1D spectrum of **2** (CD_3OD, 600 MHz). Irradiated at δ3.85 ppm (H2).

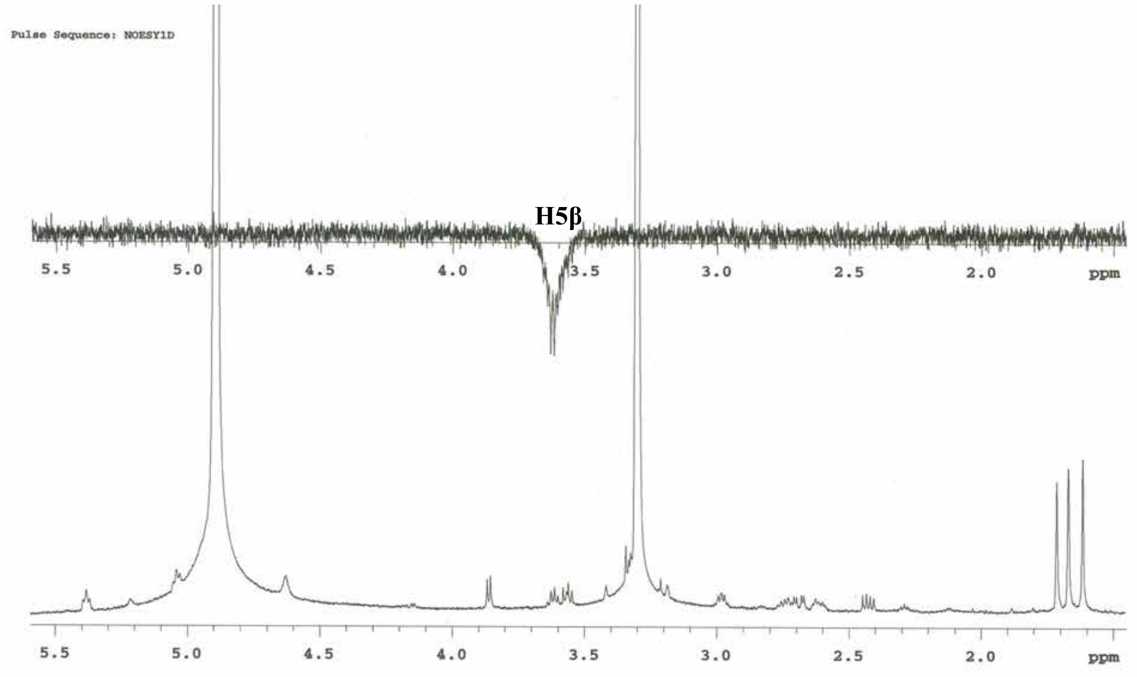

Figure S13. NOESY1D spectrum of **2** (CD_3OD, 600 MHz). Irradiated at δ3.61 ppm (H5β).

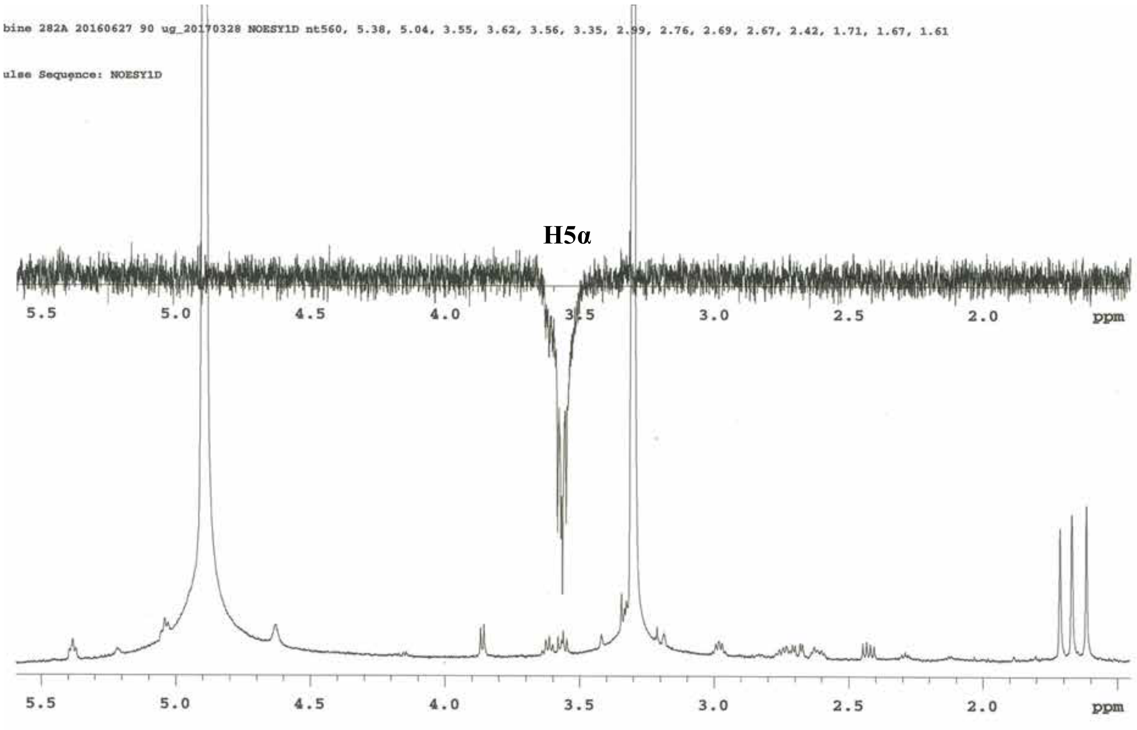

Figure S14. NOESY1D spectrum of **2** (CD_3OD, 600 MHz). Irradiated at δ3.56 ppm (H5α).

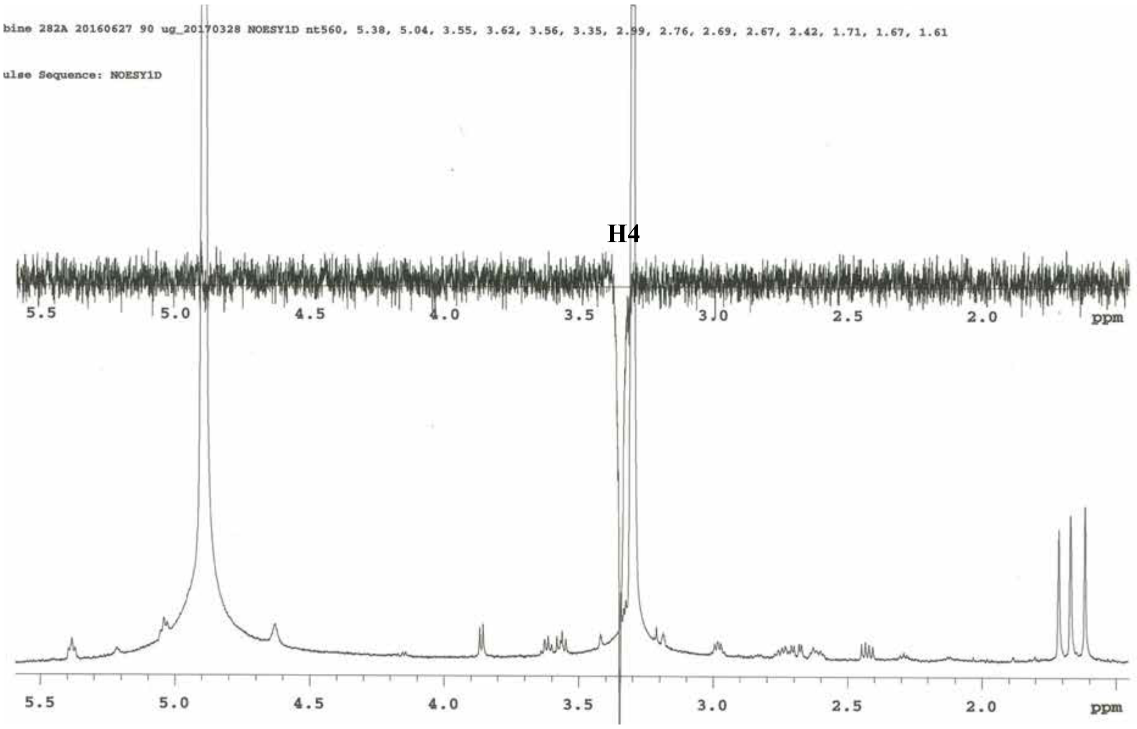

Figure S15. NOESY1D spectrum of **2** (CD_3OD, 600 MHz). Irradiated at δ3.33 ppm (H4).

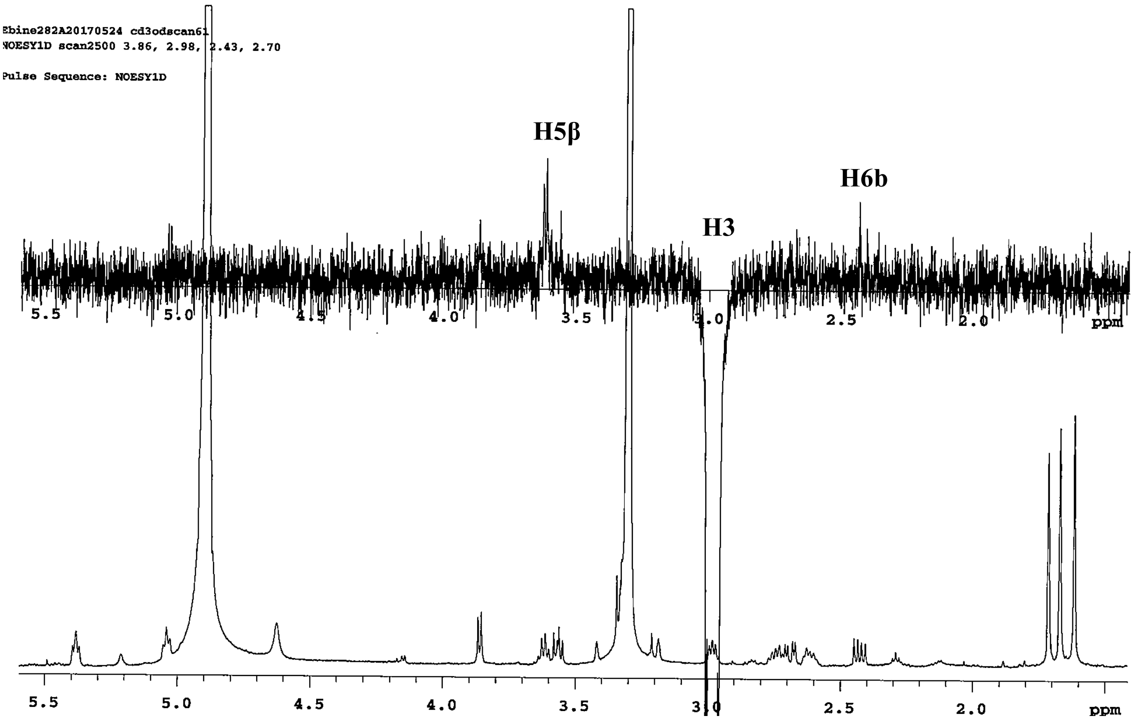

Figure S16. NOESY1D spectrum of **2** (CD_3OD, 600 MHz). Irradiated at δ2.98 ppm (H3).

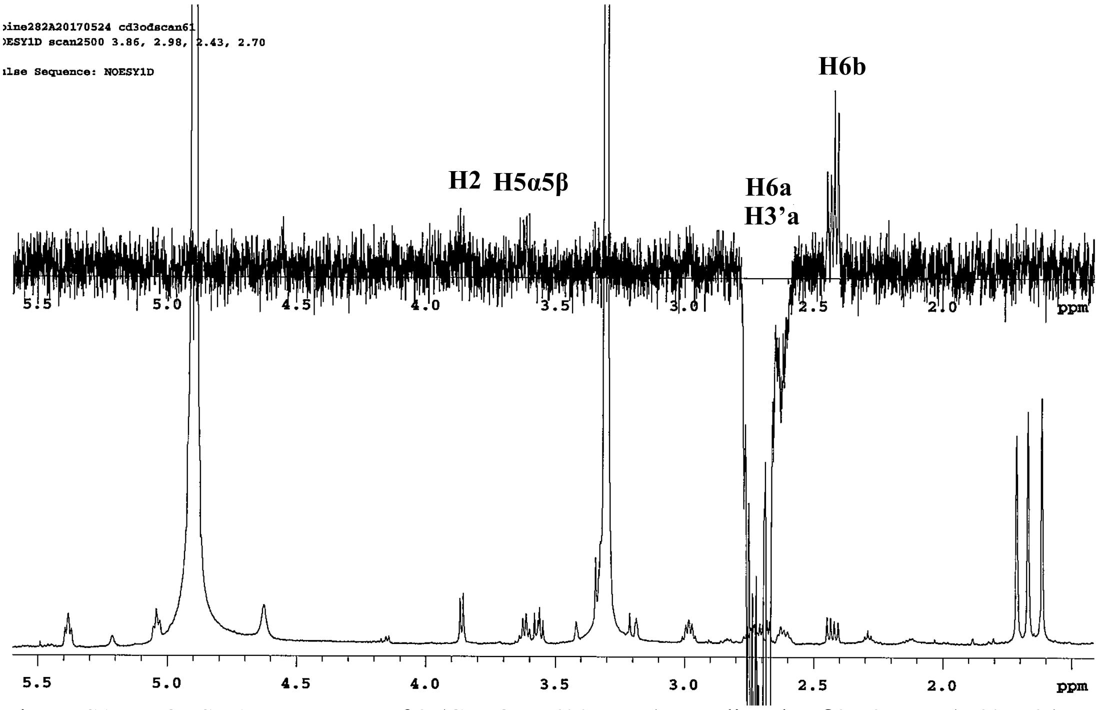

Figure S17. NOESY1D spectrum of **2** (CD_3OD, 600 MHz). Irradiated at δ2.73 ppm (H3'a, 6a).

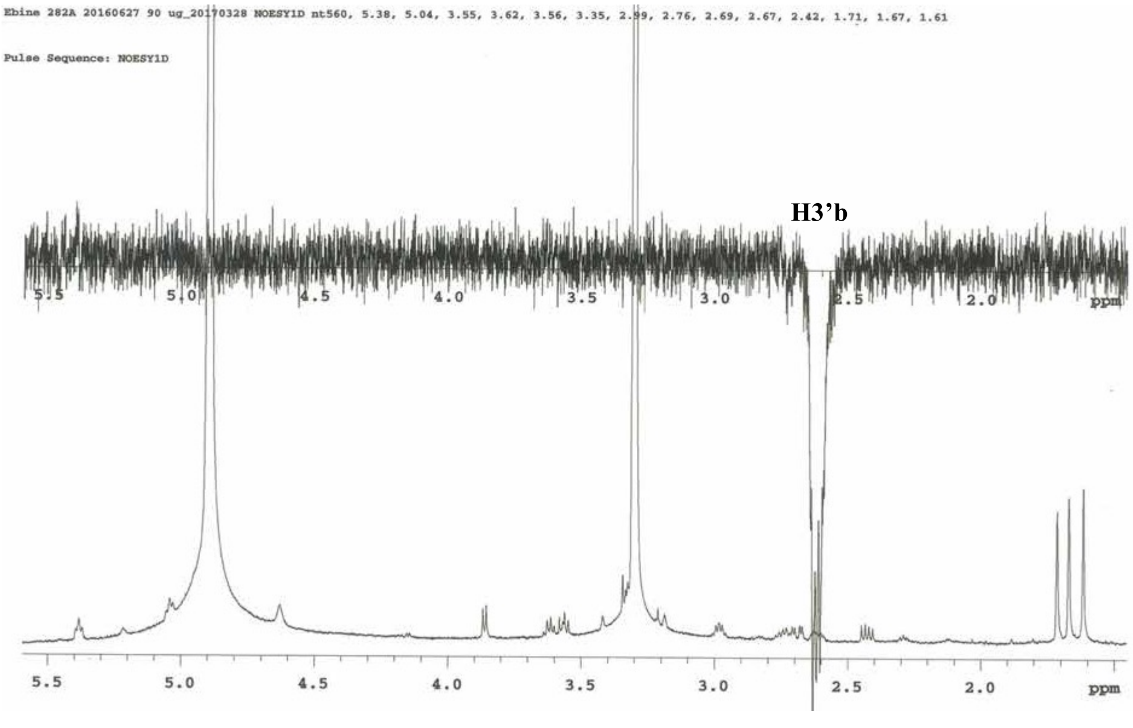

Figure S18. NOESY1D spectrum of **2** (CD_3OD, 600 MHz). Irradiated at δ2.61 ppm (H3'b).

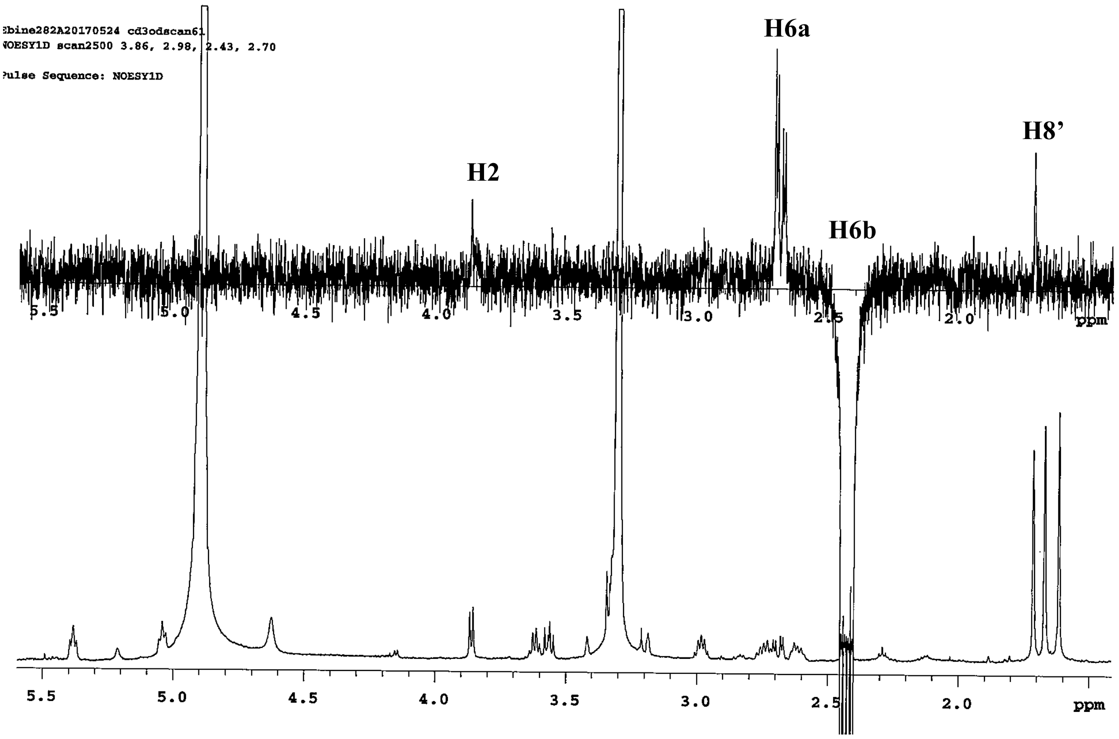

Figure S19. NOESY1D spectrum of **2** (CD_3OD, 600 MHz). Irradiated at δ2.42 ppm (H6b).

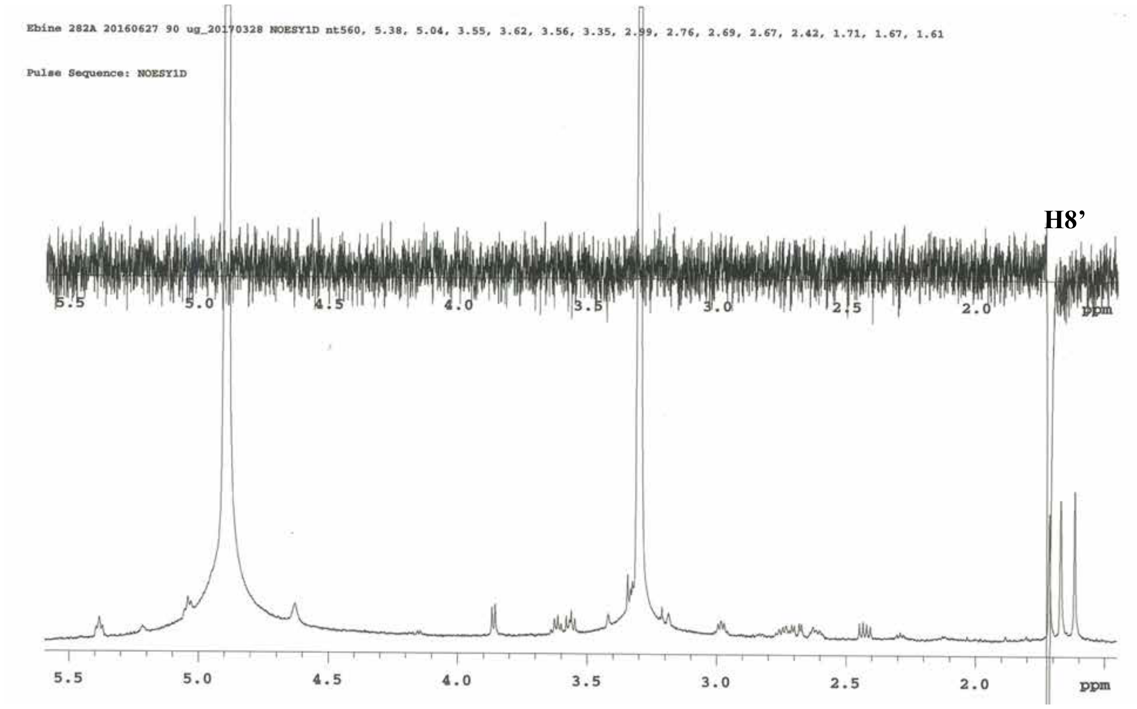

Figure S20. NOESY1D spectrum of **2** (CD_3OD, 600 MHz). Irradiated at δ1.71 ppm (H8’).

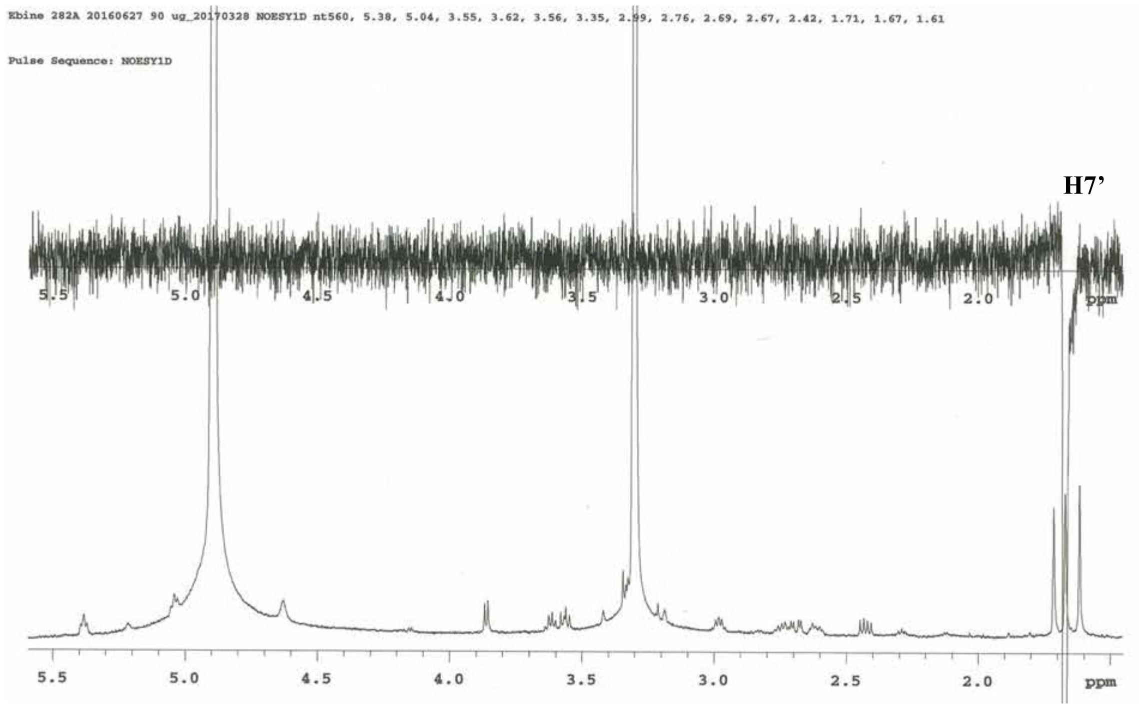

Figure S21. NOESY1D spectrum of **2** (CD_3OD, 600 MHz). Irradiated at δ1.67 ppm (H7').

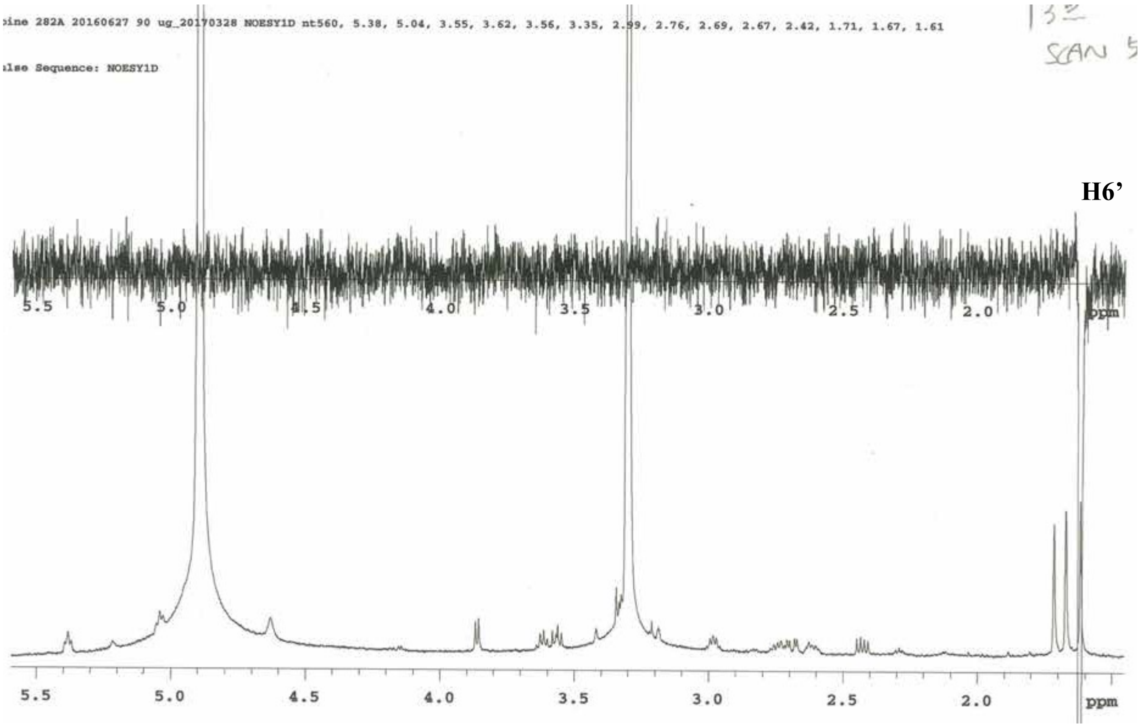

Figure S22. NOESY1D spectrum of **2** (CD_3OD, 600 MHz). Irradiated at δ1.61 ppm (H6').

・NMR spectra of 7’-methyl-isodomoic acid B (**3**).

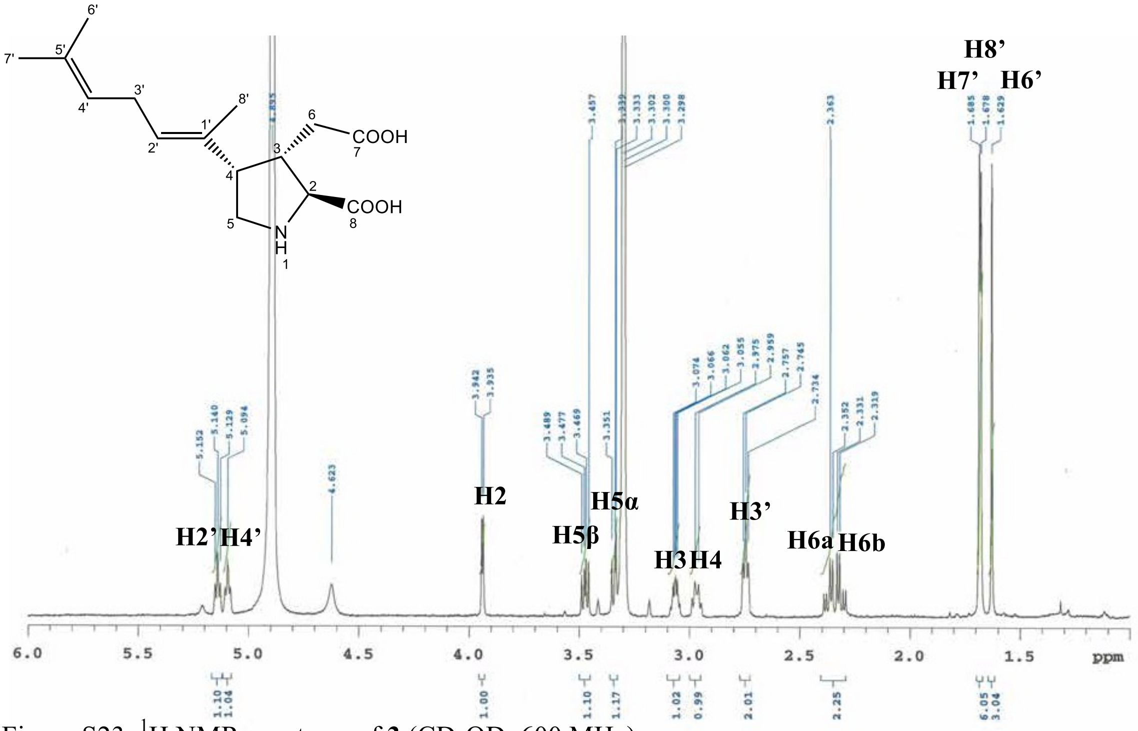

Figure S23. ^{1}H NMR spectrum of **3** (CD_3OD, 600 MHz).

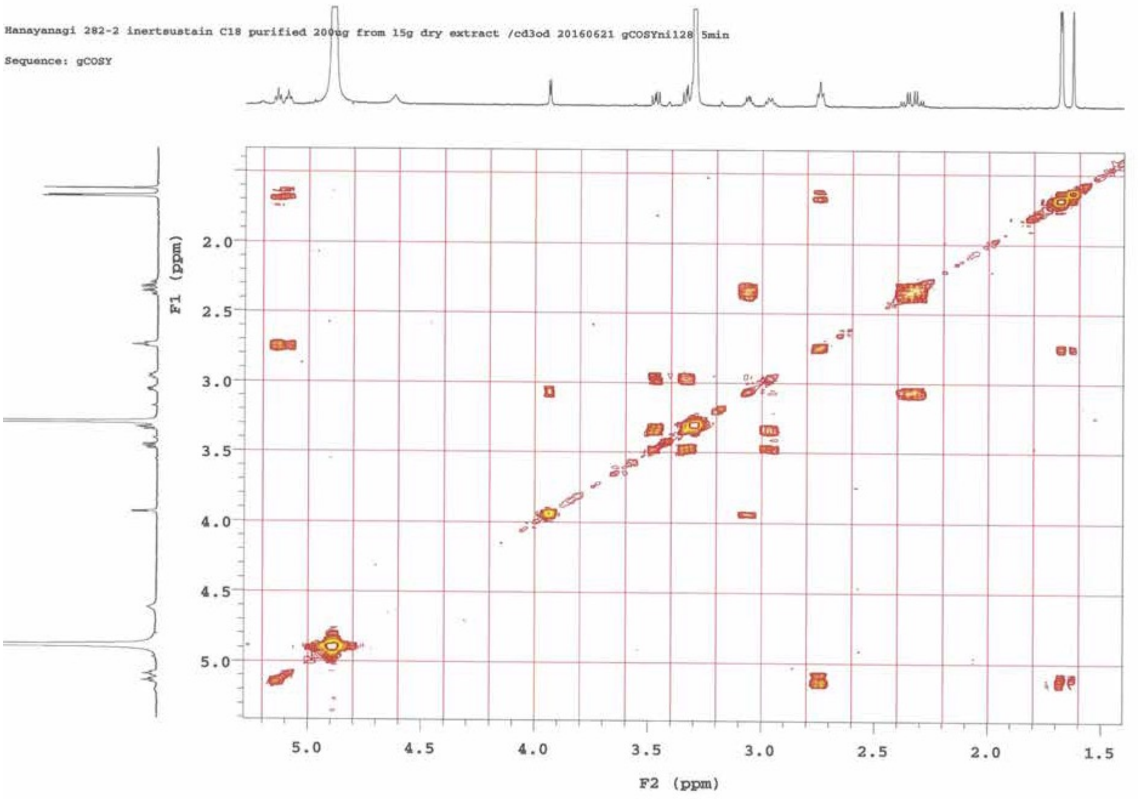

Figure S24. Gradient COSY spectrum of **3** (CD_3OD, 600 MHz).

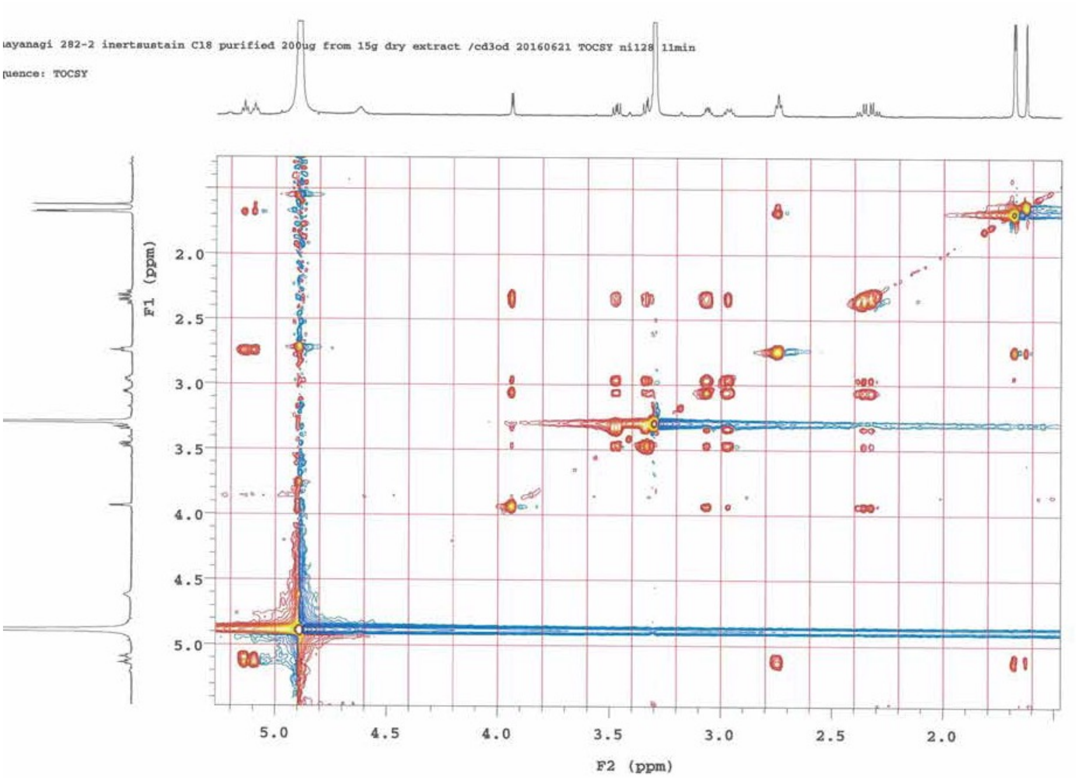

Figure S25. TOCSY spectrum of **3** (CD_3OD, 600 MHz).

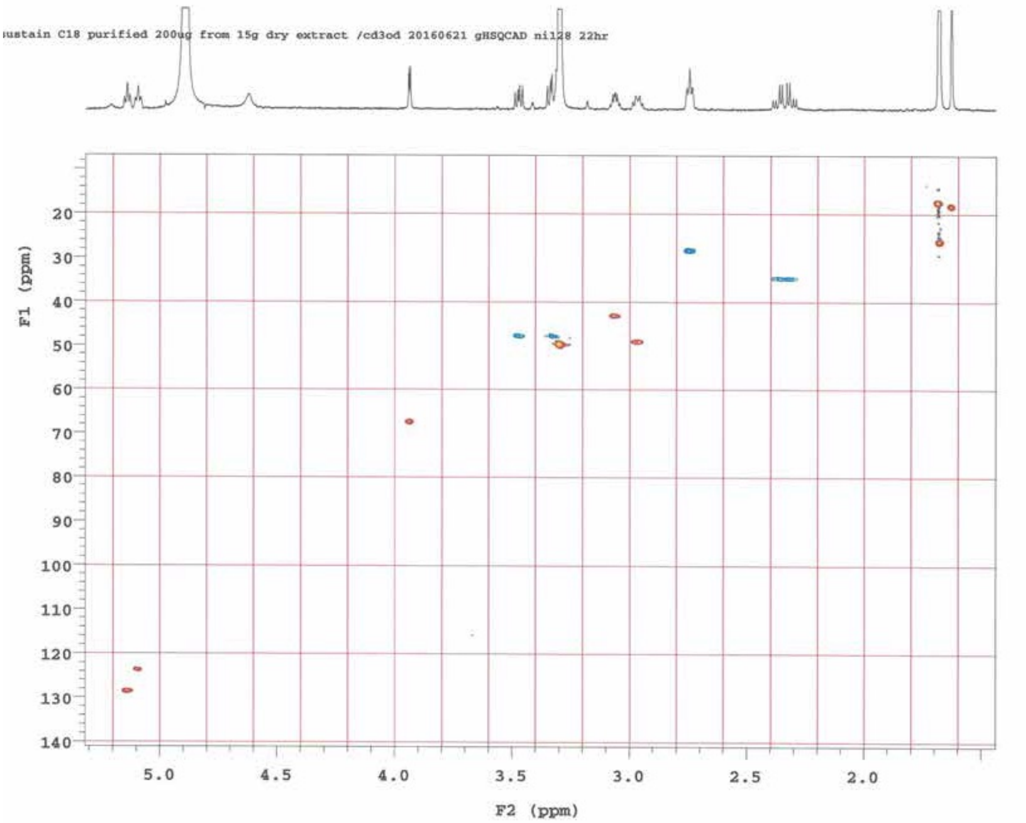

Figure S26. Gradient HSQC spectrum of **3** (CD_3OD, 600 MHz).

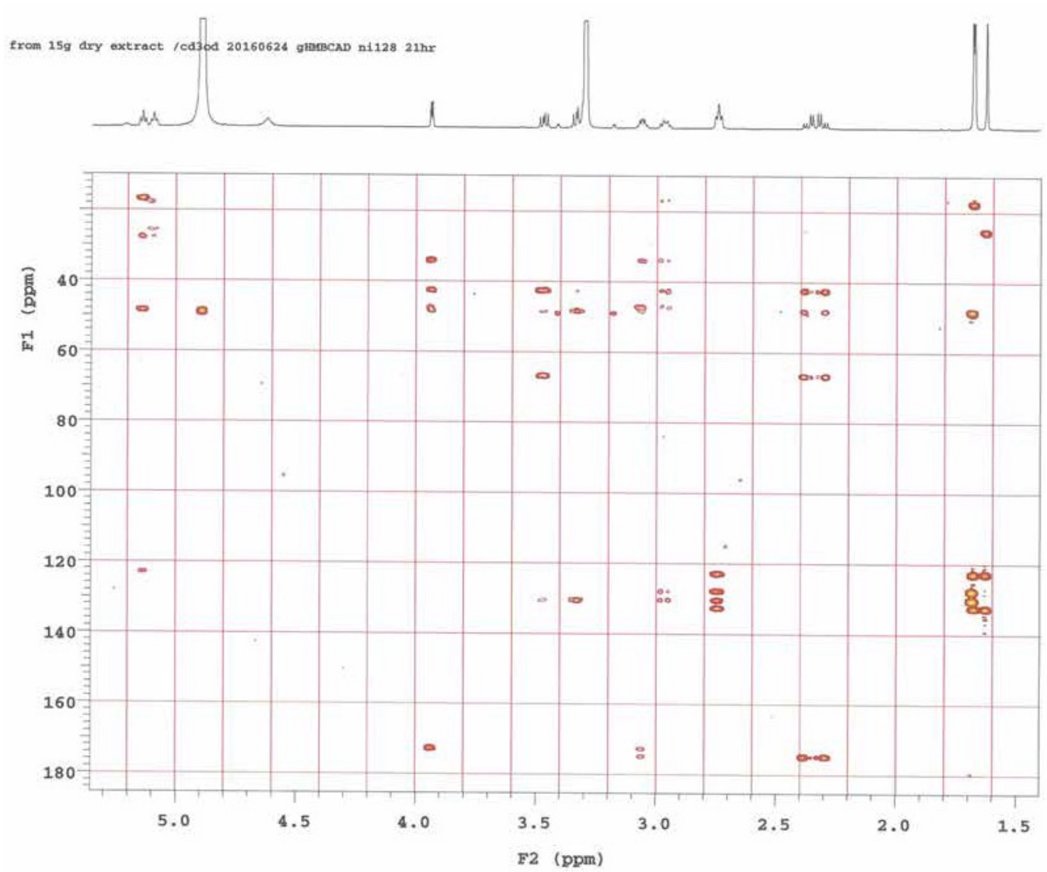

Figure S27. Gradient HMBC spectrum of **3** (CD_3OD, 600 MHz).

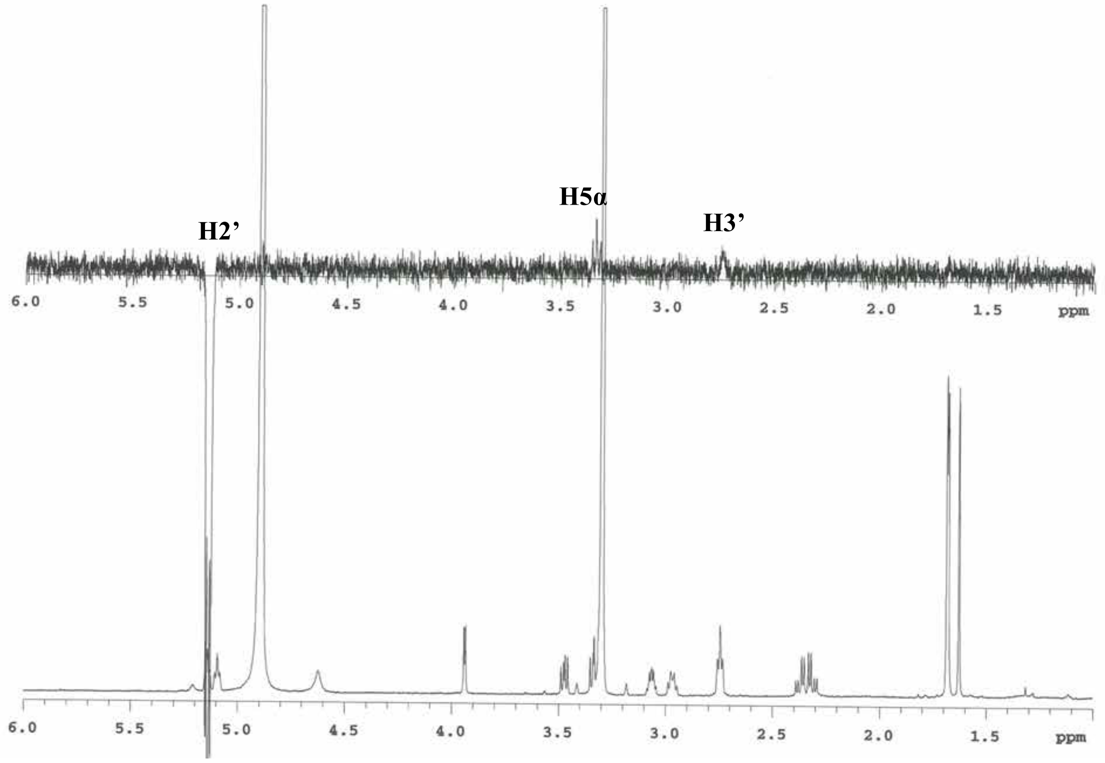

Figure S28. NOESY1D spectrum of **3** (CD_3OD, 600 MHz). Irradiated at δ5.14 ppm (H2’) and DHO.

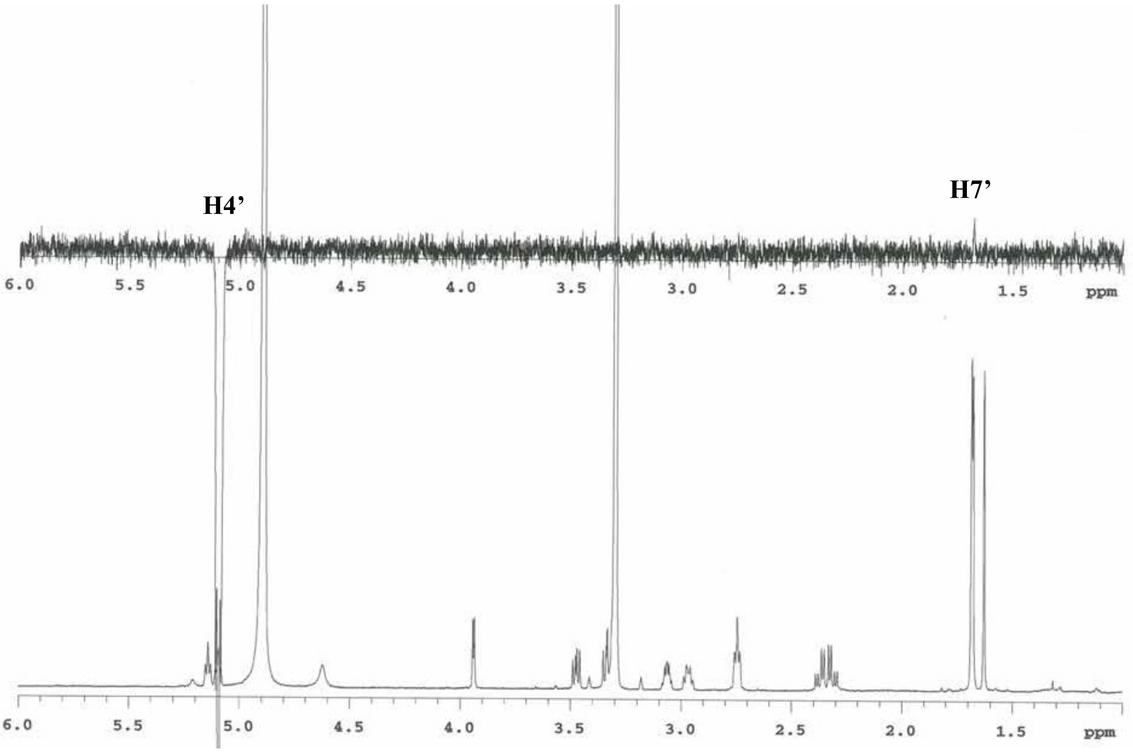

Figure S29. NOESY1D spectrum of **3** (CD_3OD, 600 MHz). Irradiated at δ5.09 ppm (H4’).

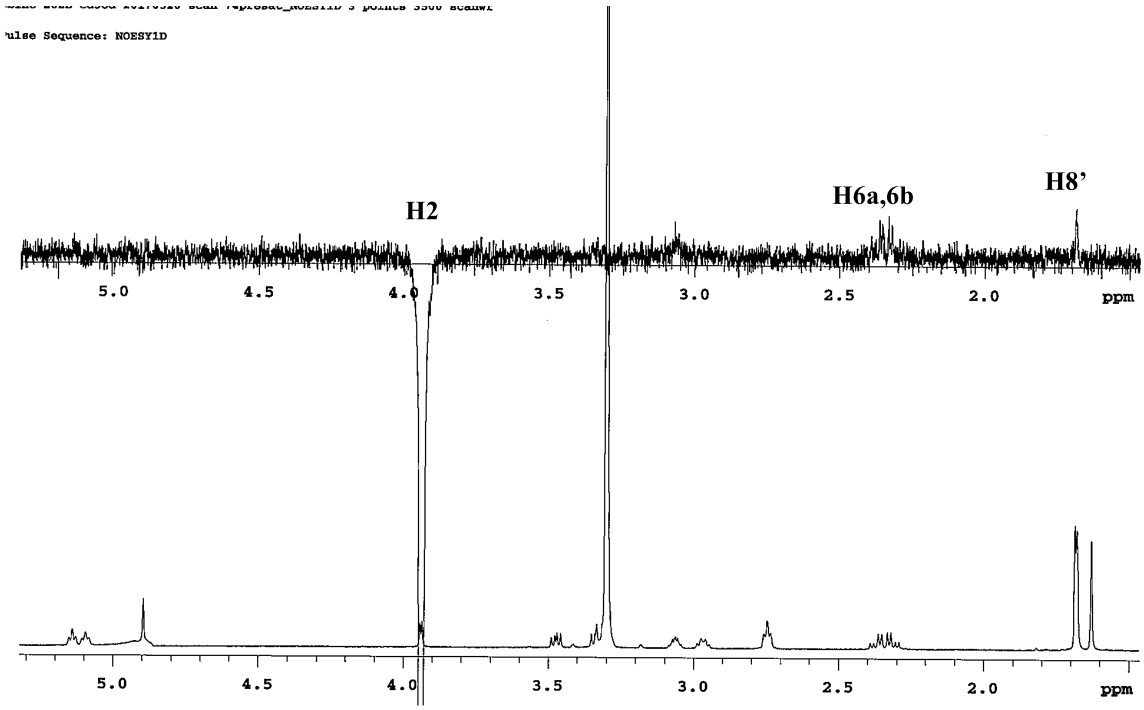

Figure S30. NOESY1D spectrum of **3** (CD_3OD, 600 MHz). Irradiated at δ3.93 ppm (H2).

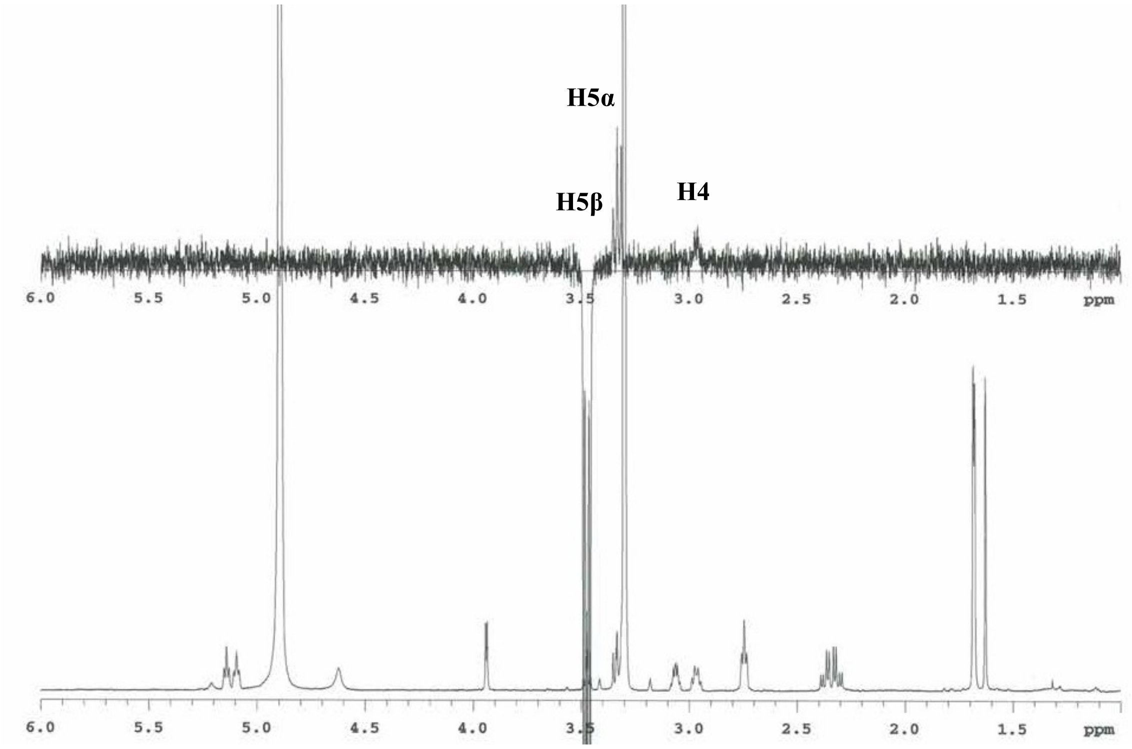

Figure S31. NOESY1D spectrum of **3** (CD_3OD, 600 MHz). Irradiated at δ3.47 ppm (H5β).

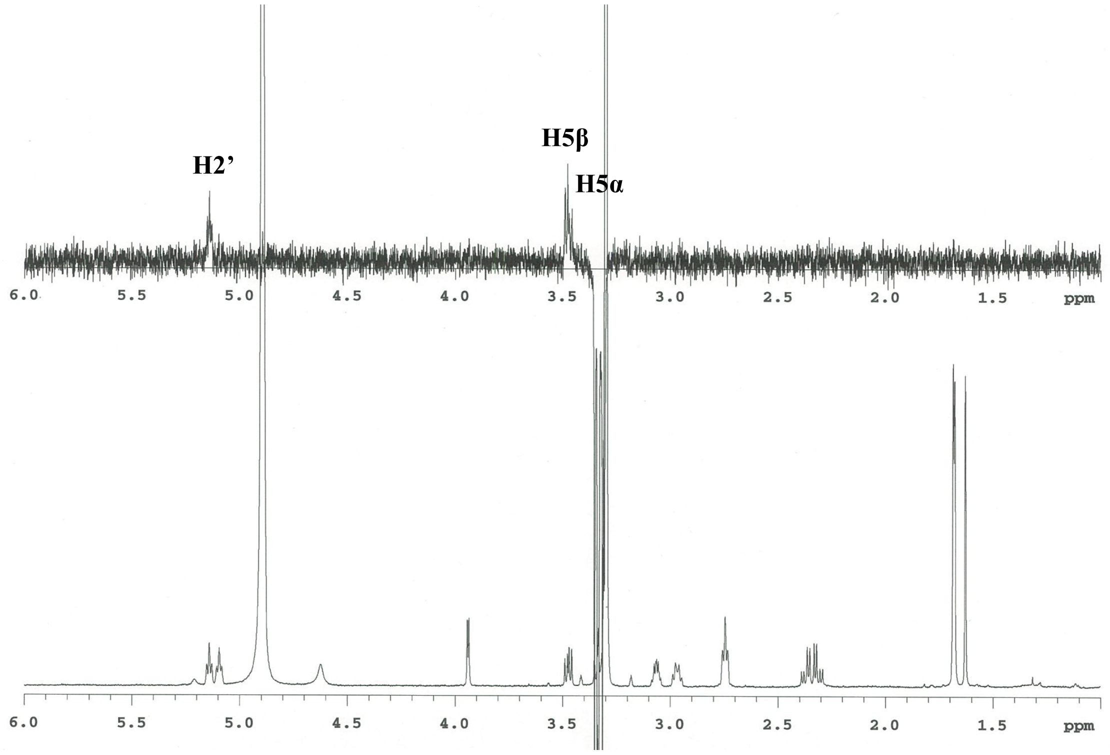

Figure S32. NOESY1D spectrum of **3** (CD_3OD, 600 MHz). Irradiated at δ3.33 ppm (H5α).

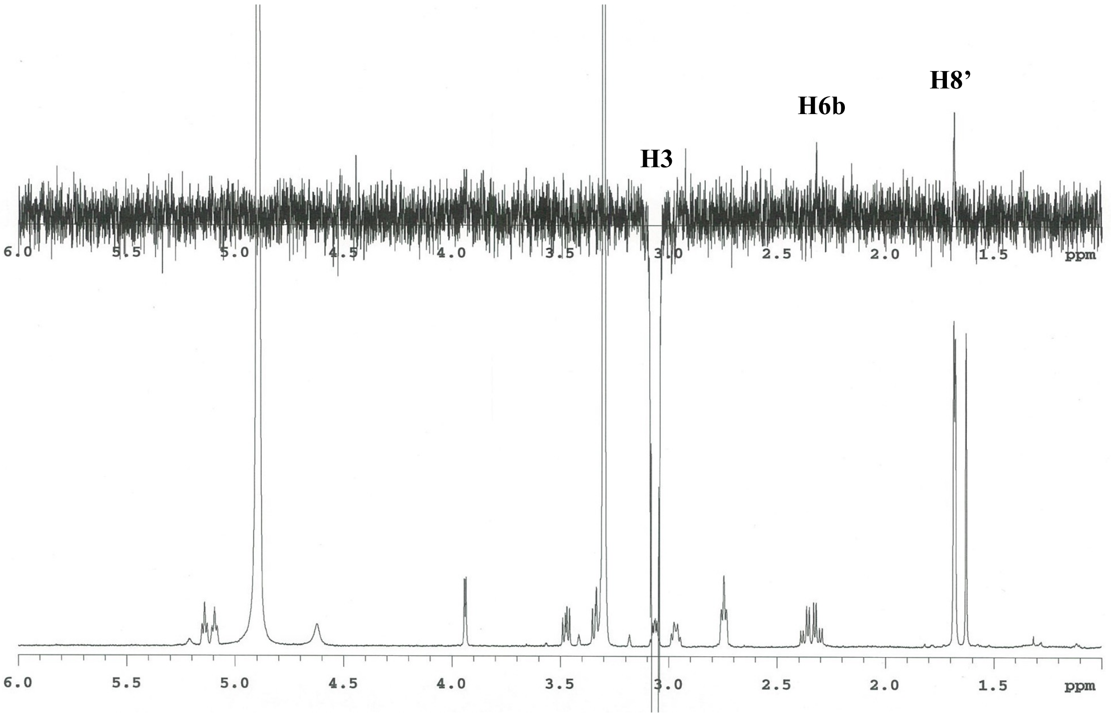

Figure S33. NOESY1D spectrum of **3** (CD_3OD, 600 MHz). Irradiated at δ3.06 ppm (H3).

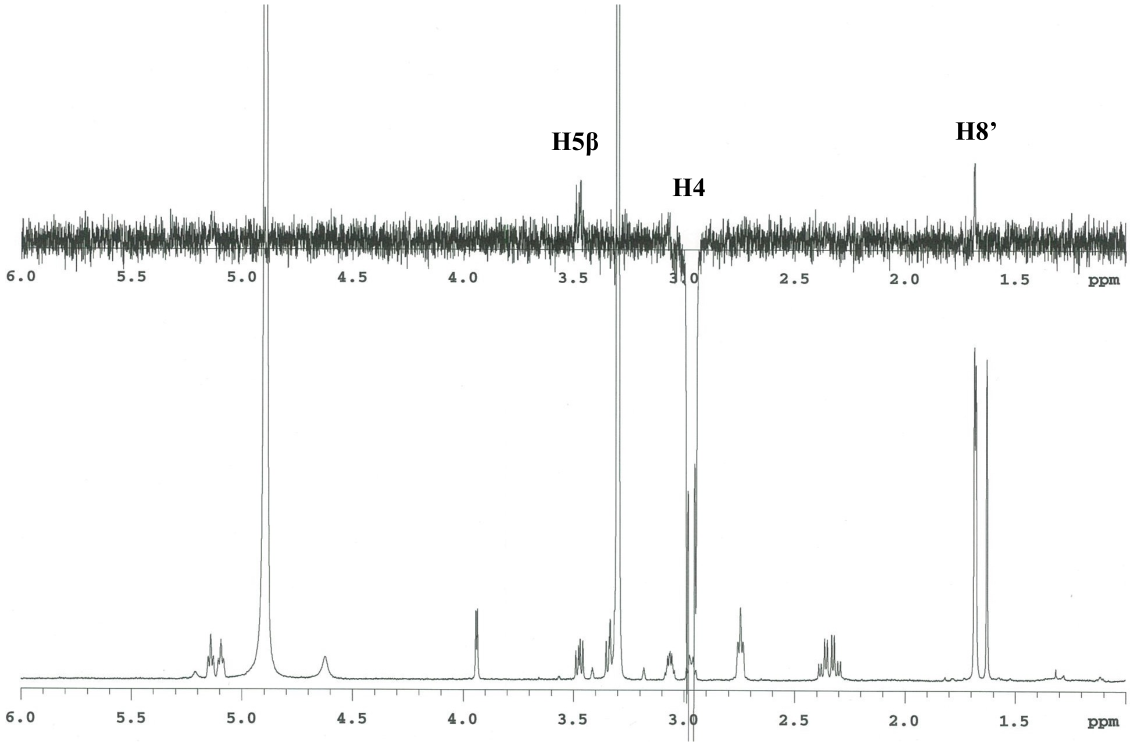

Figure S34. NOESY1D spectrum of **3** (CD_3OD, 600 MHz). Irradiated at δ2.97 ppm (H4).

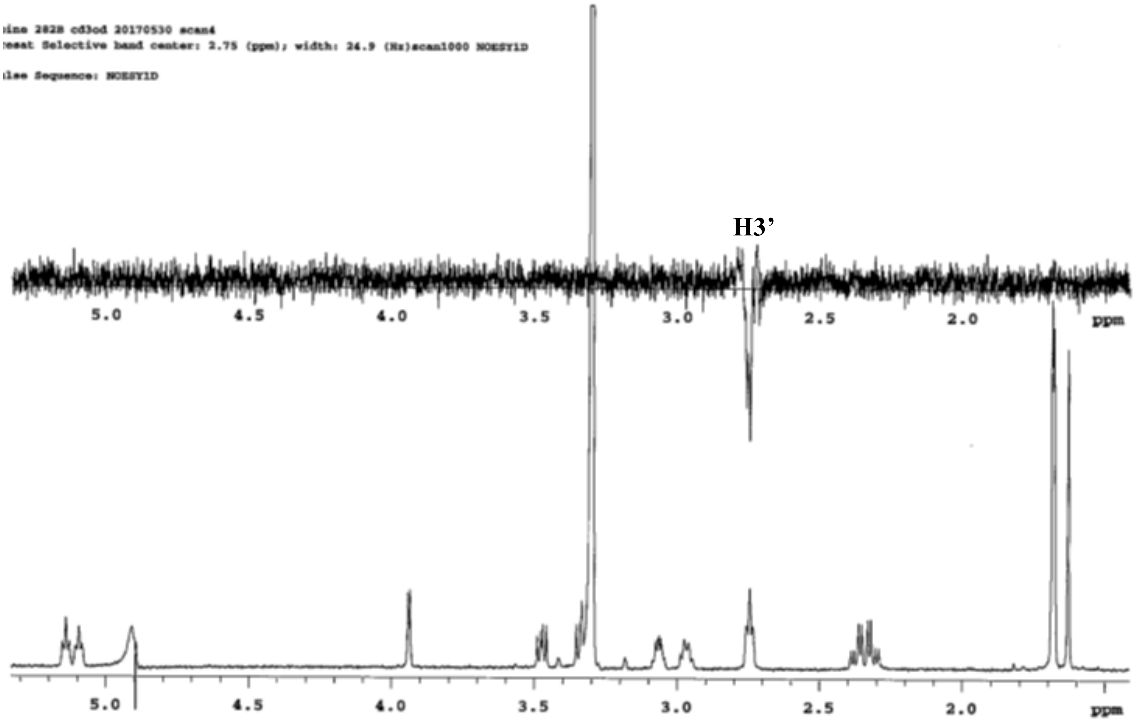

Figure S35. NOESY1D spectrum of **3** (CD_3OD, 600 MHz). Irradiated at δ2.73 ppm (H3') and HDO.

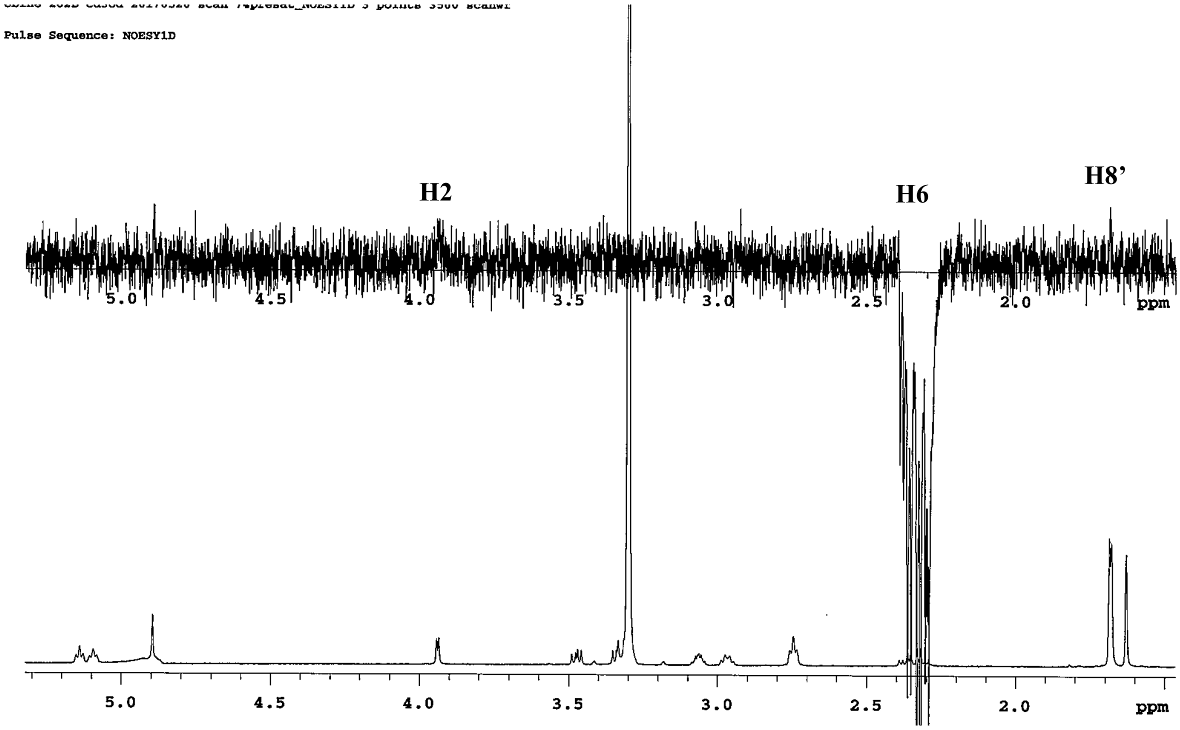

Figure S36. NOESY1D spectrum of **3** (CD_3OD, 600 MHz). Irradiated at δ2.33 ppm (H6) and HDO.

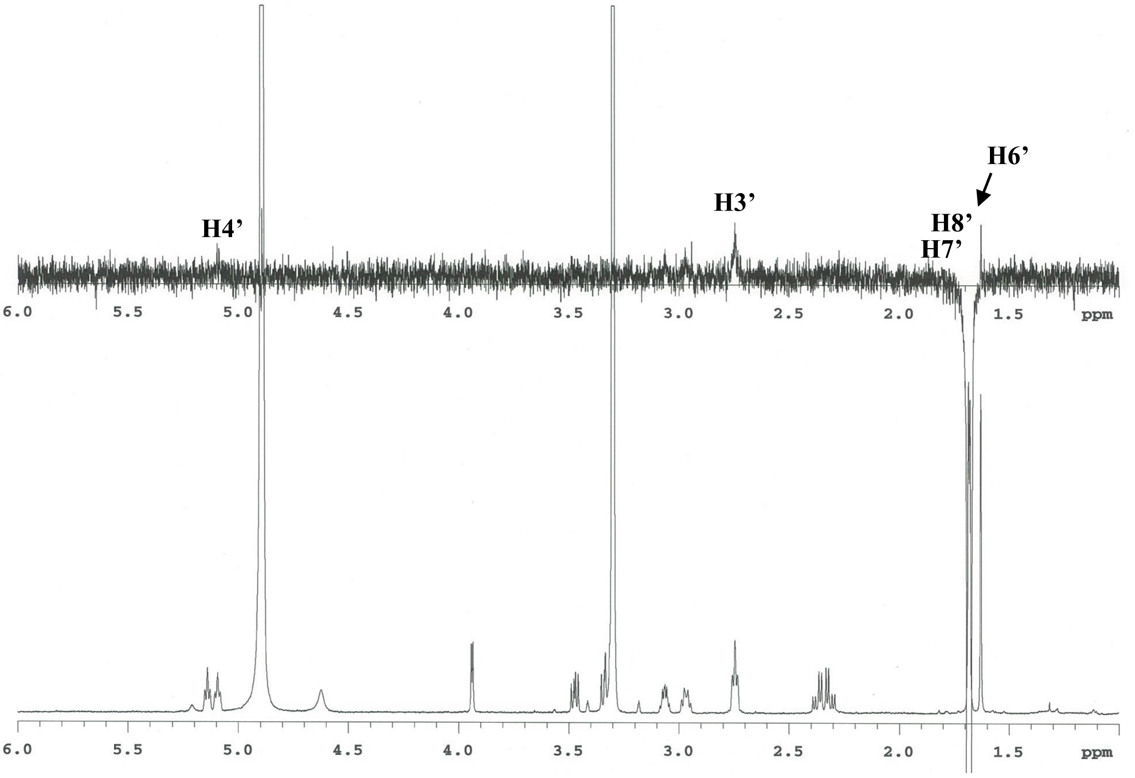

Figure S37. NOESY1D spectrum of **3** (CD_3OD, 600 MHz). Irradiated at δ1.68 ppm (H7', H8').

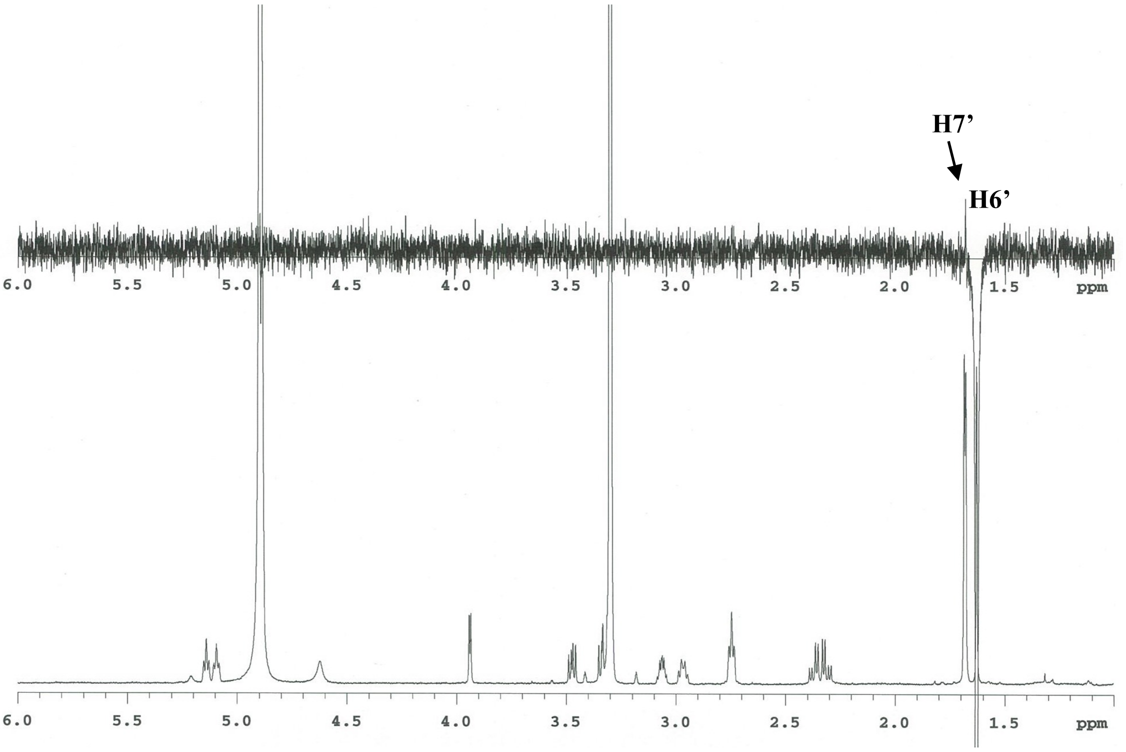

Figure S38. NOESY1D spectrum of **3** (CD_3OD, 600 MHz). Irradiated at δ1.63 ppm (H6').

・NMR spectra of *N*-geranyl-L-glutamic acid (**4**).

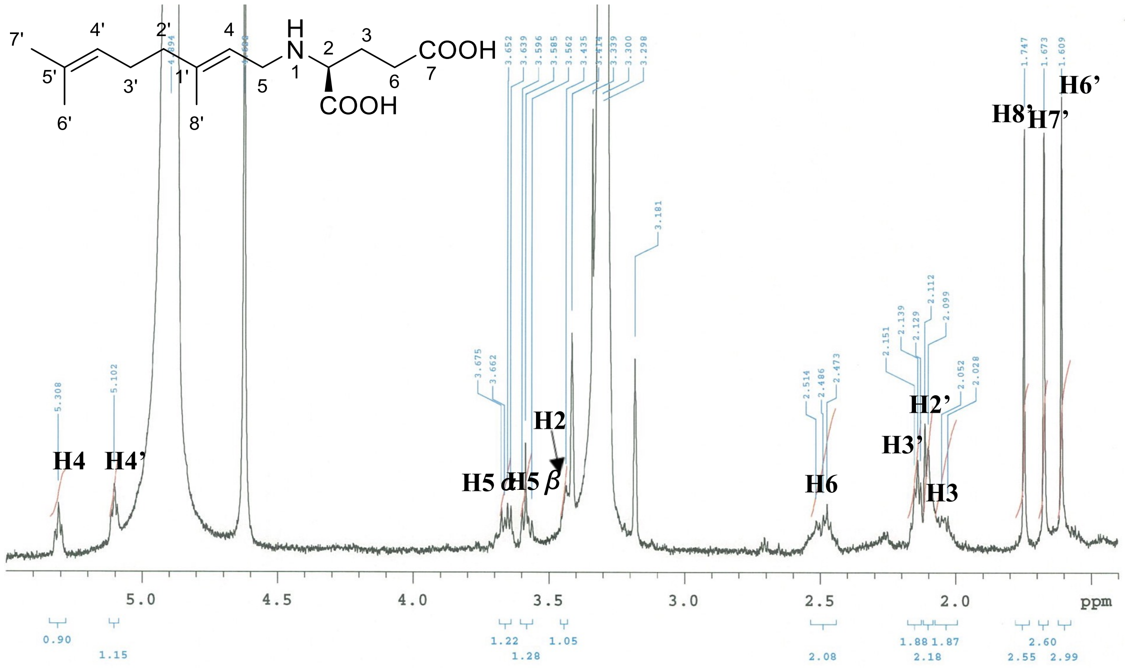

Figure S39. ^{1}H NMR spectrum of **4** (CD_3OD, 600 MHz).

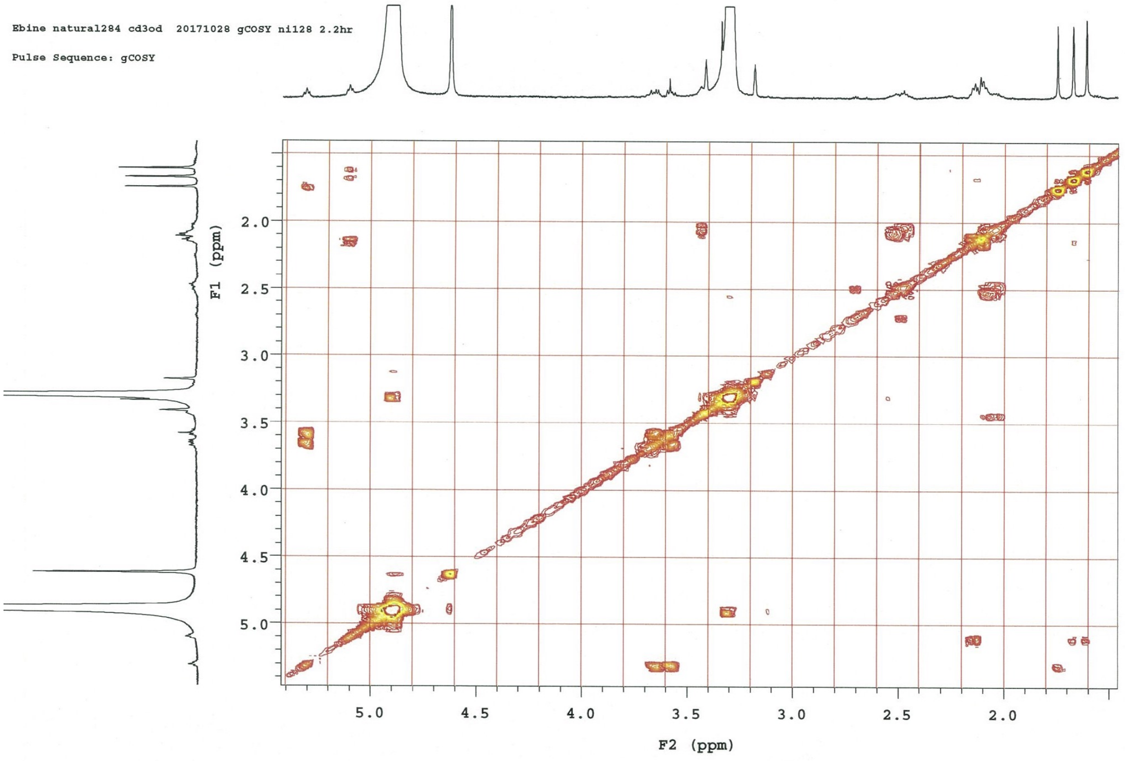

Figure S40. Gradient COSY spectrum of **4** (CD_3OD, 600 MHz).

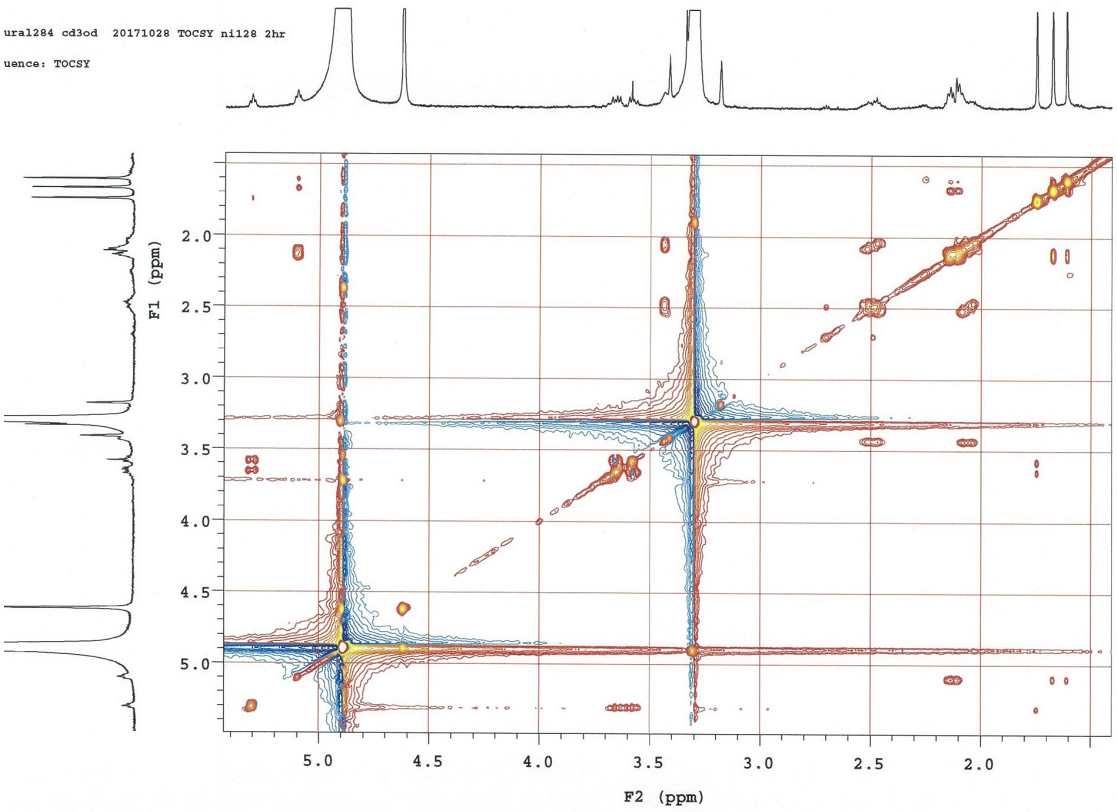

Figure S41. TOCSY spectrum of **4** (CD_3OD, 600 MHz).

・NMR spectra of 7’-hydroxymethyl-isodomoic acid A (**5**).

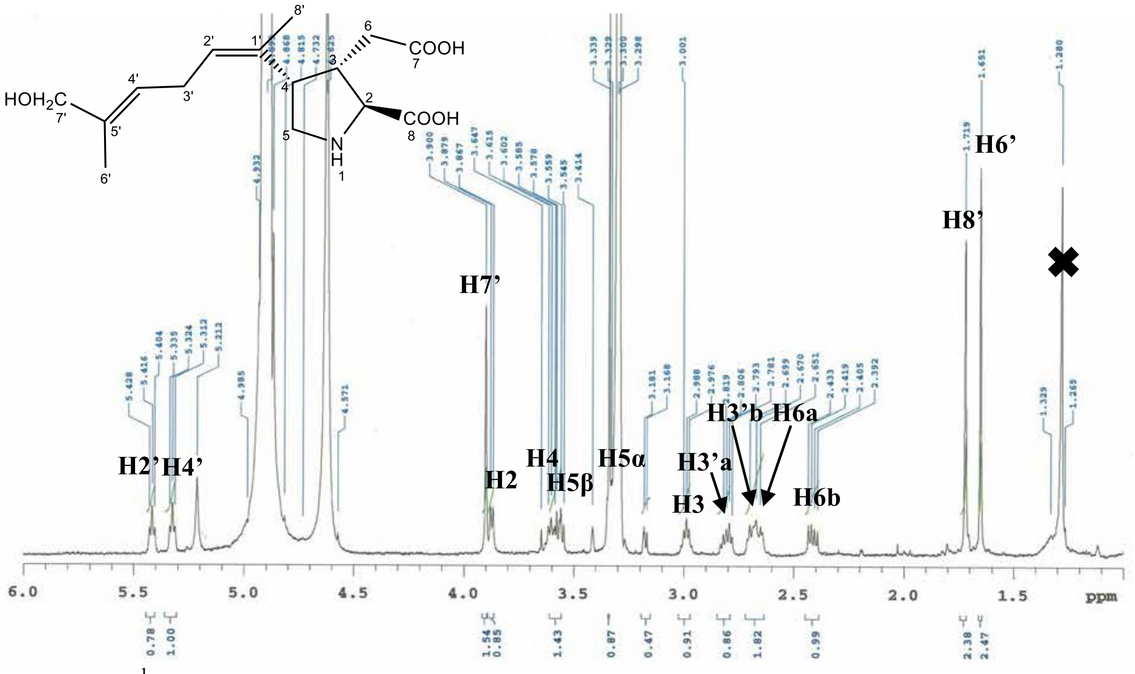

Figure S42. ^{1}H NMR spectrum of **5** (CD_3OD, 600 MHz).

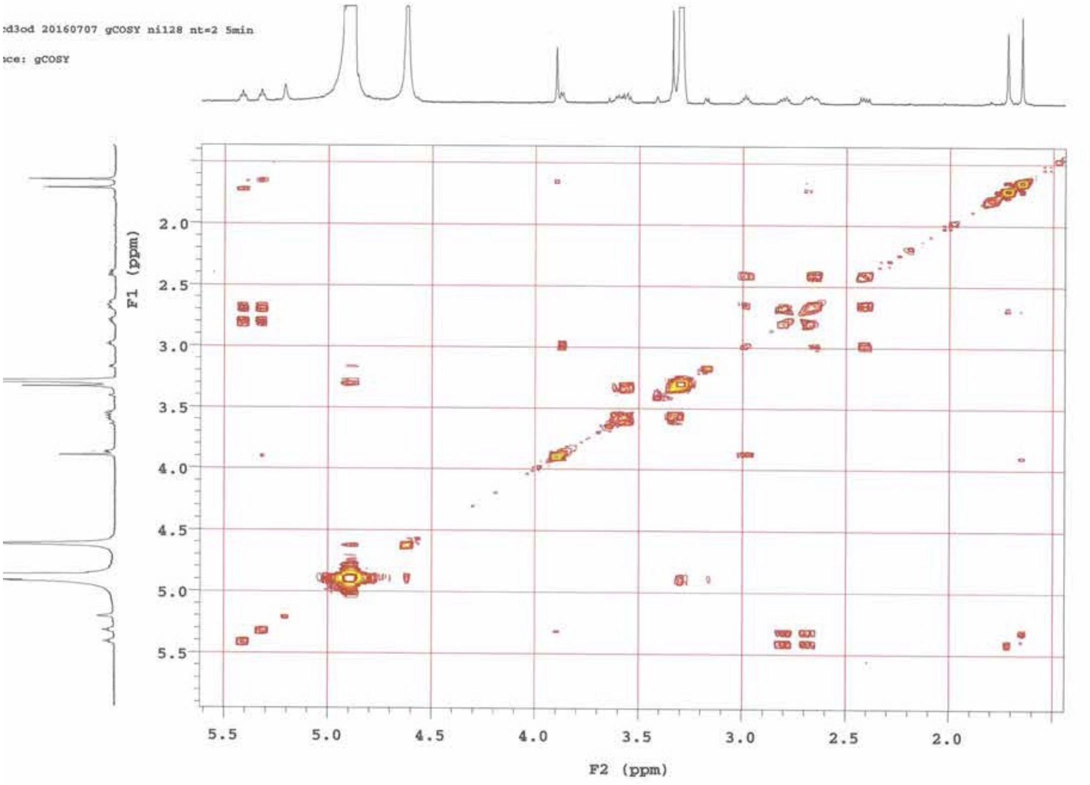

Figure S43. Gradient COSY spectrum of **5** (CD_3OD, 600 MHz).

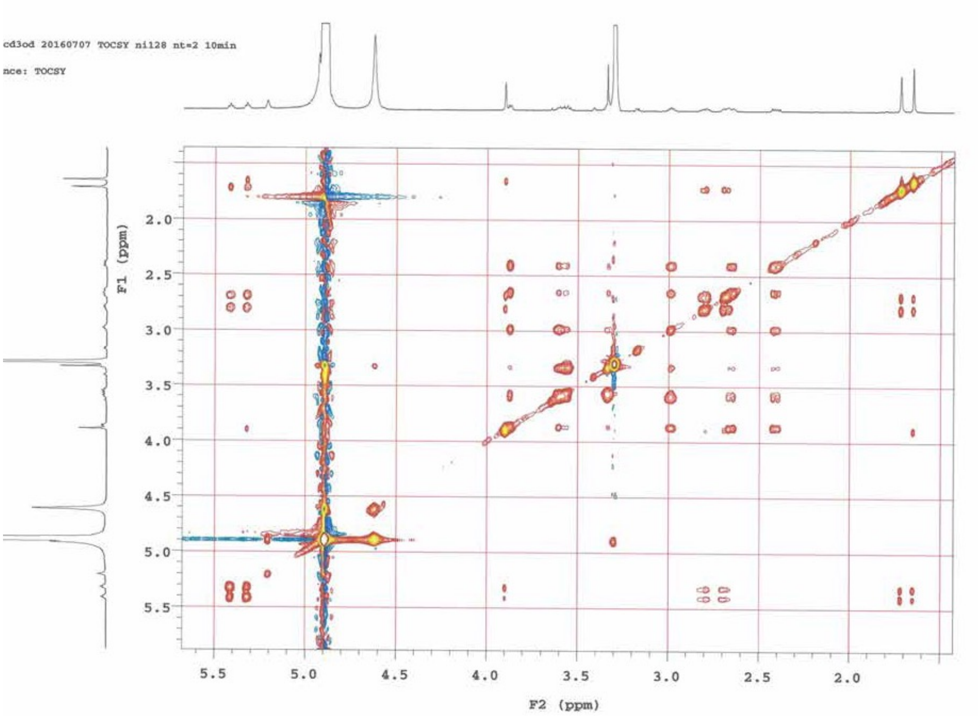

Figure S44. TOCSY spectrum of **5** (CD_3OD, 600 MHz).

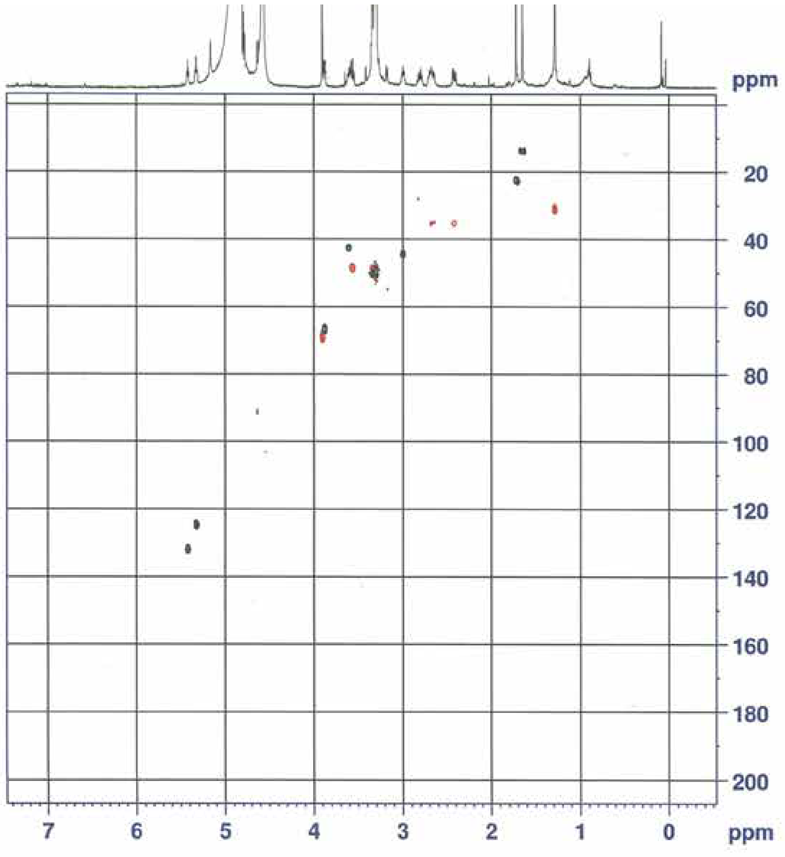

Figure S45. Gradient HSQC spectrum of **5** (CD_3OD, 600 MHz, CryoProbe).

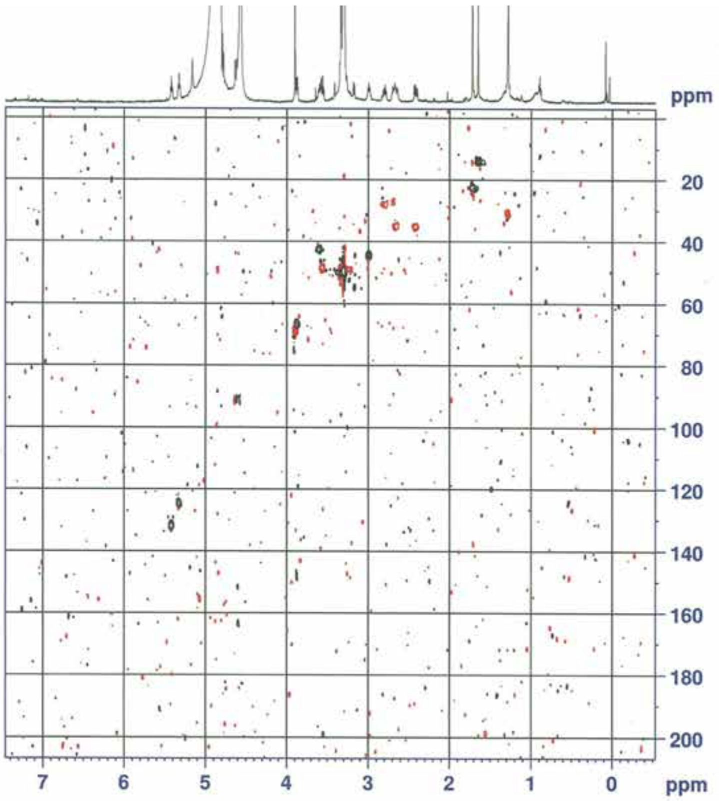

Figure S46. Gradient HSQC spectrum of **5** (magnified) (CD_3OD, 600 MHz, CryoProbe).

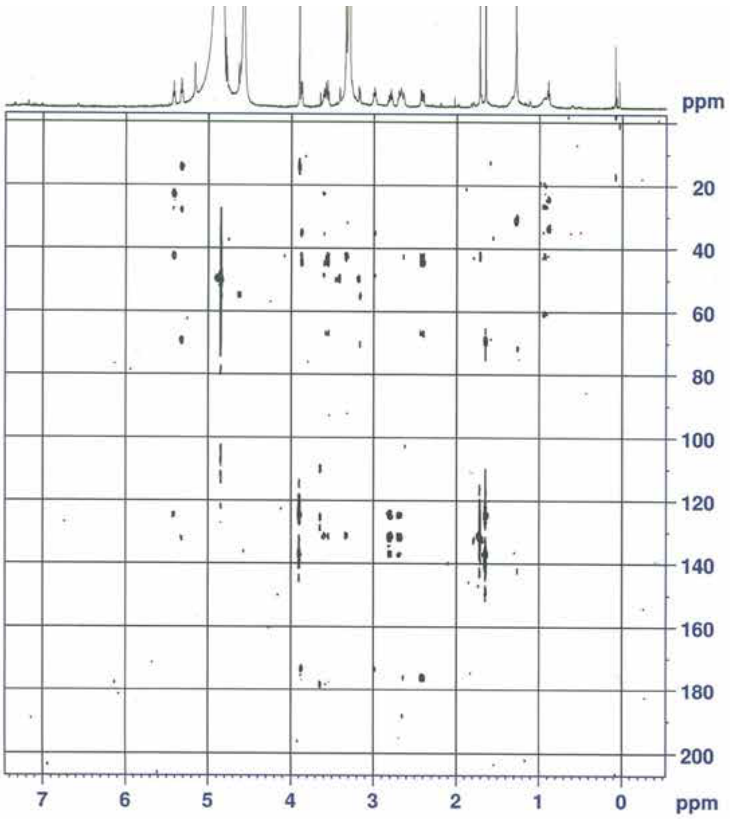

Figure S47. Gradient HMBC spectrum of **5** (CD_3OD, 600 MHz, CryoProbe).

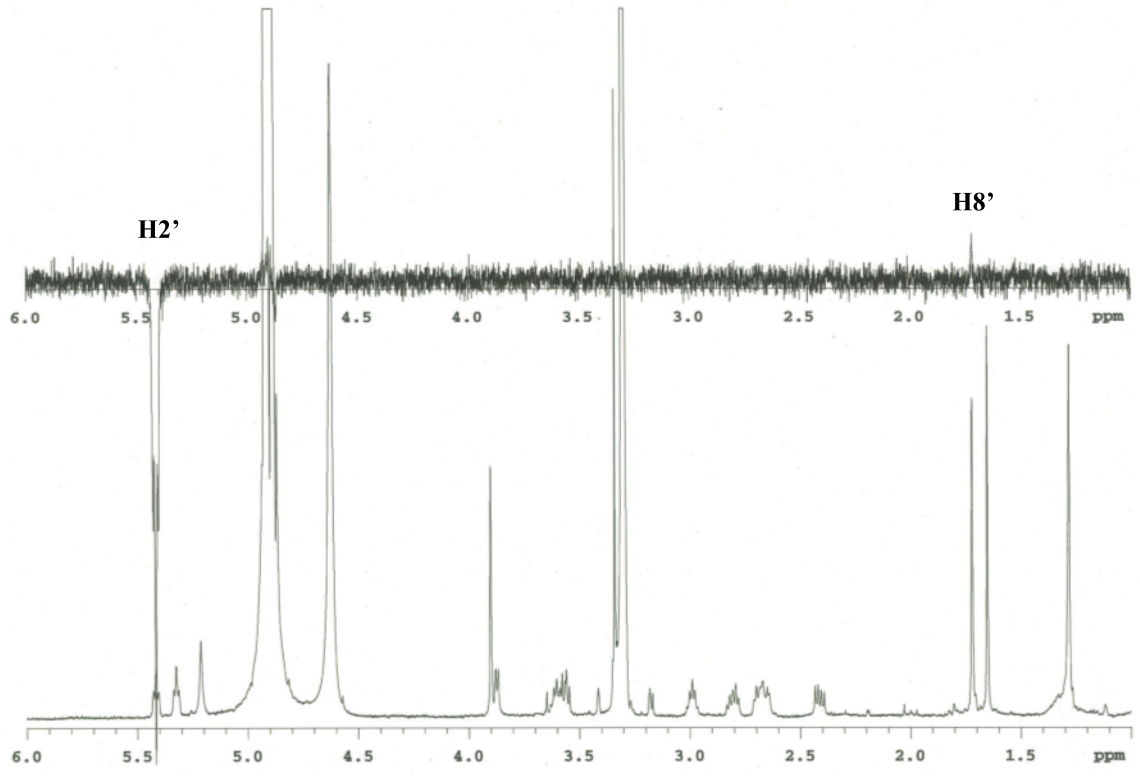

Figure S48. NOESY1D spectrum of **5** (CD_3OD, 600 MHz). Irradiated at δ5.41 ppm (H2').

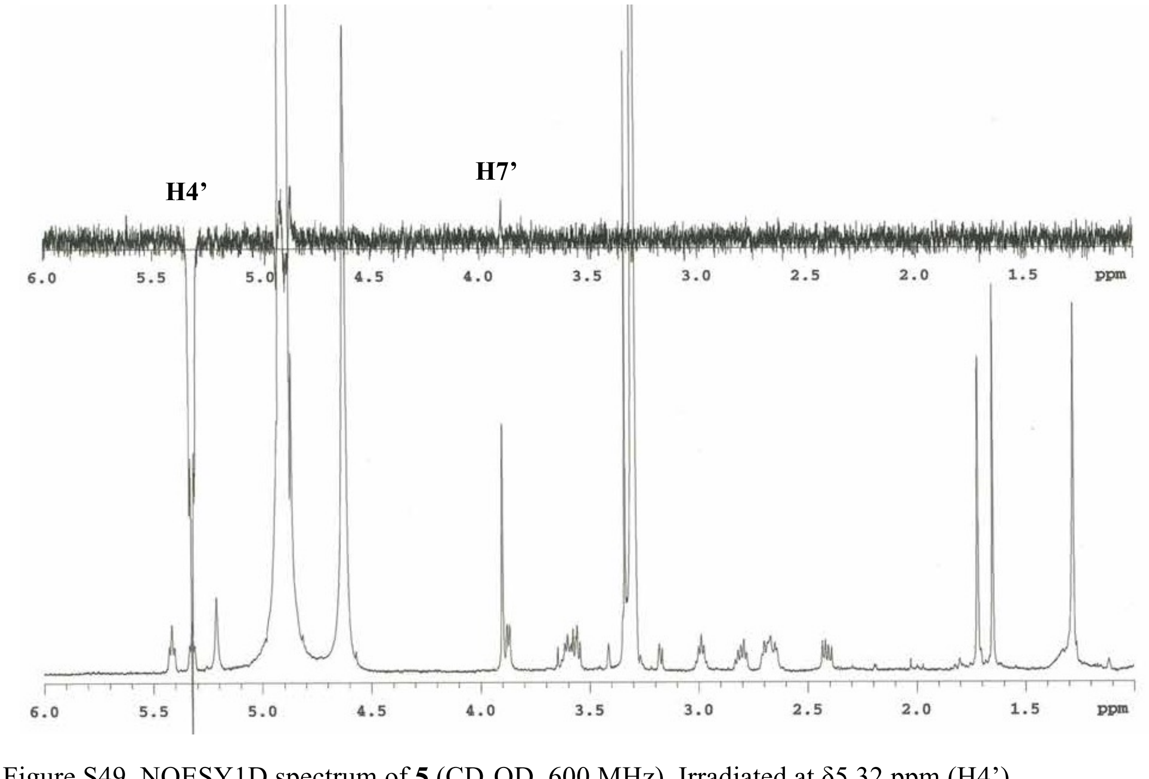

Figure S49. NOESY1D spectrum of **5** (CD_3OD, 600 MHz). Irradiated at δ5.32 ppm (H4').

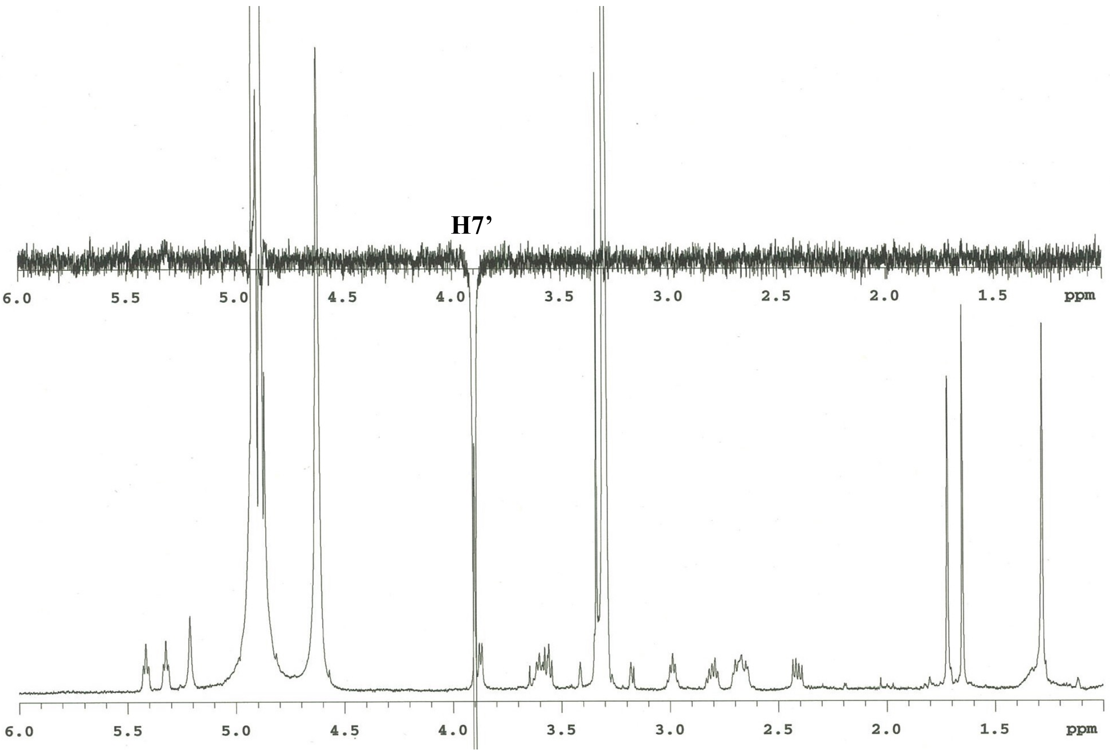

Figure S50. NOESY1D spectrum of **5** (CD_3OD, 600 MHz). Irradiated at δ3.90 ppm (H7’).

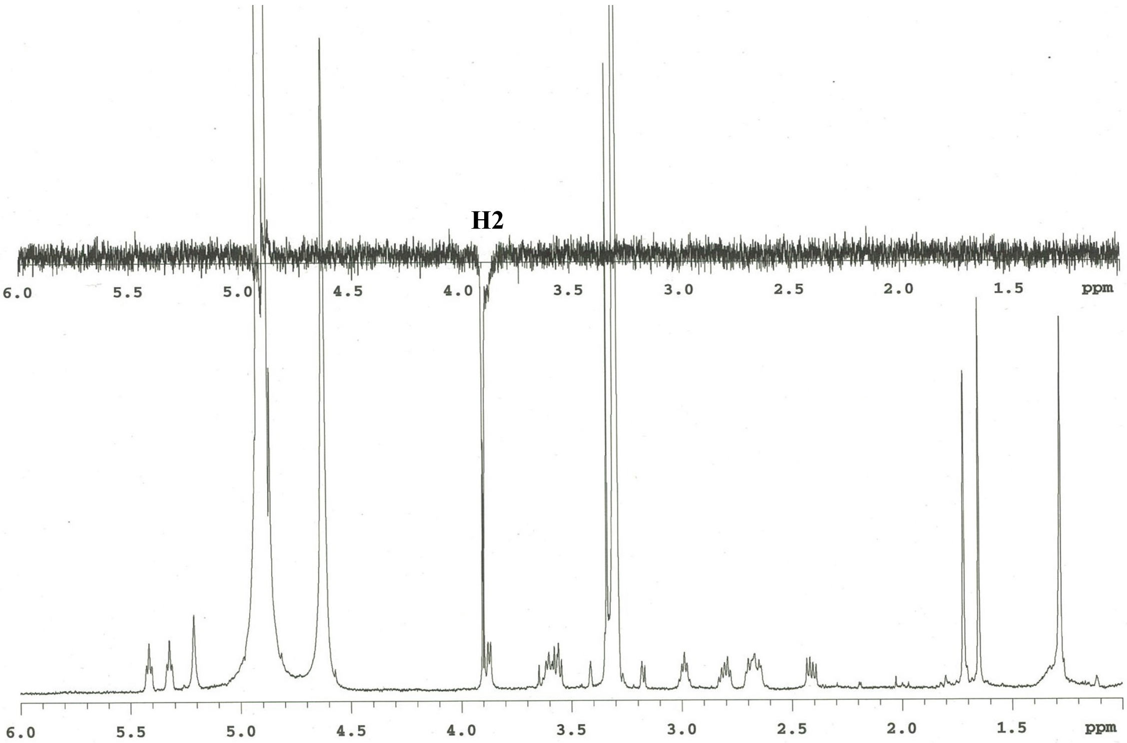

Figure S51. NOESY1D spectrum of **5** (CD_3OD, 600 MHz). Irradiated at δ3.89 ppm (H2).

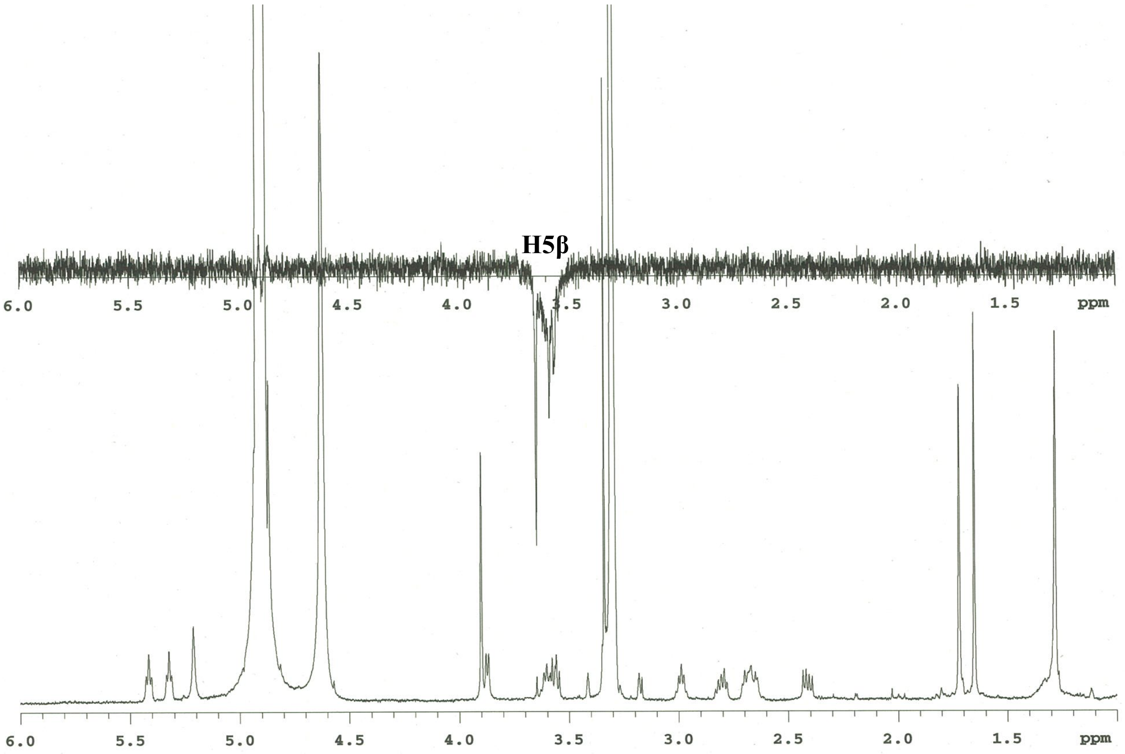

Figure S52. NOESY1D spectrum of **5** (CD_3OD, 600 MHz). Irradiated at δ3.65 ppm (H5β).

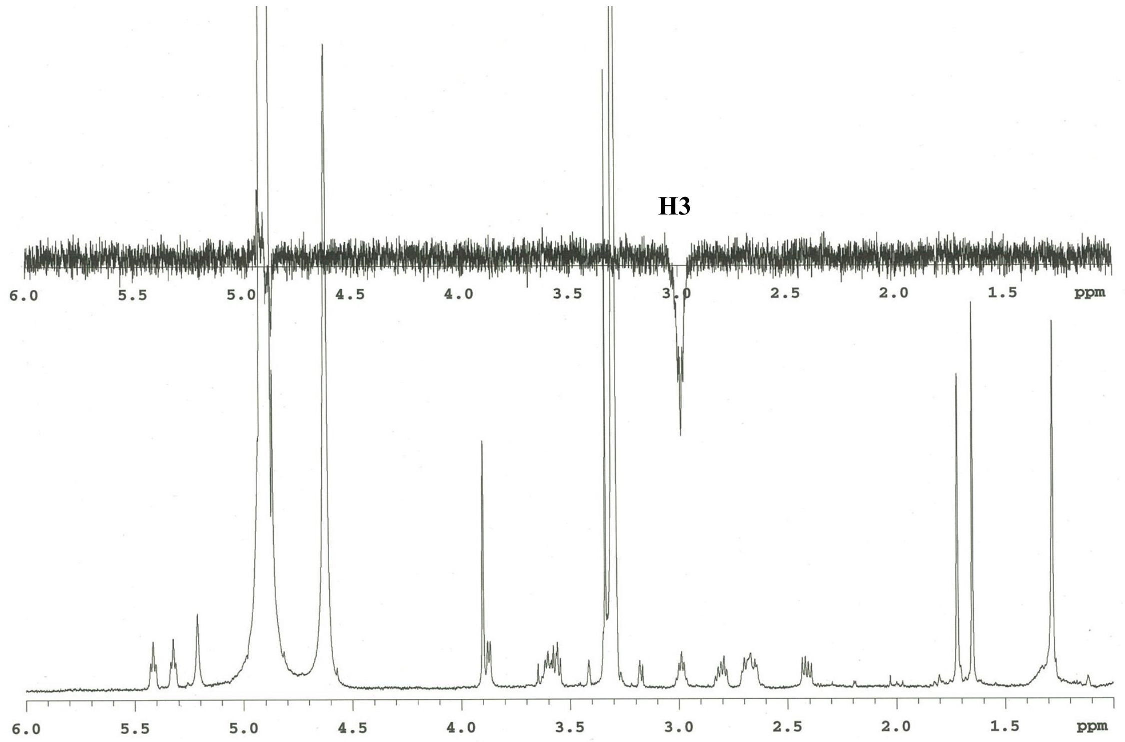

Figure S53. NOESY1D spectrum of **5** (CD_3OD, 600 MHz). Irradiated at δ2.99 ppm (H3).

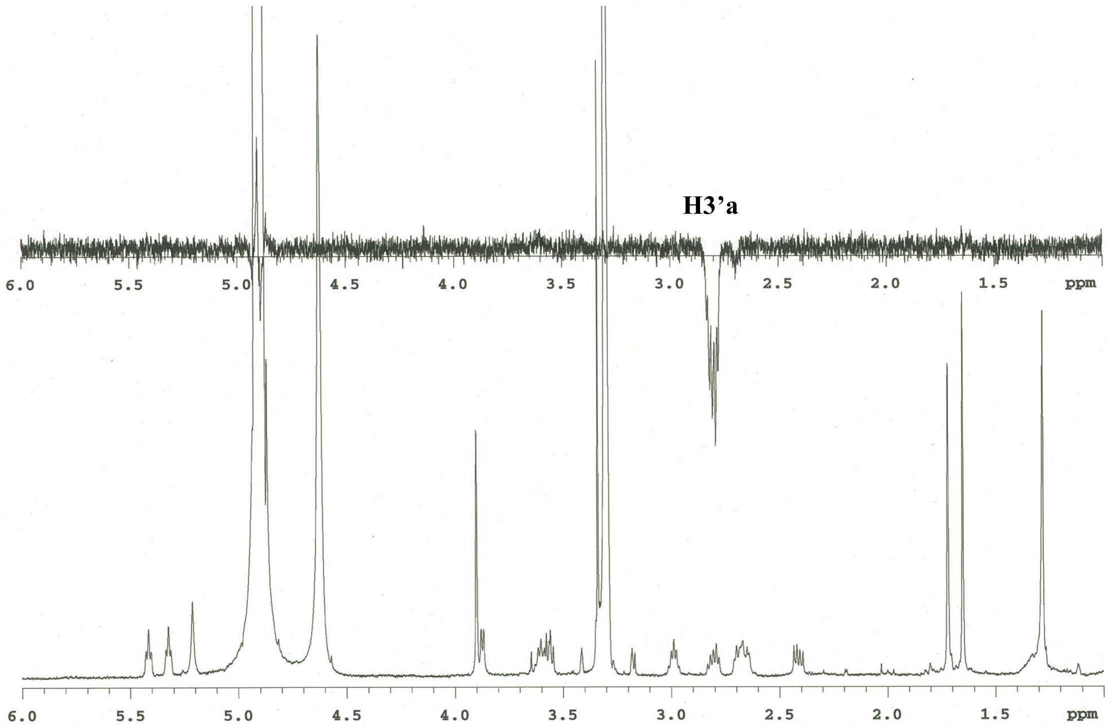

Figure S54. NOESY1D spectrum of **5** (CD_3OD, 600 MHz). Irradiated at δ2.79 ppm (H3'a).

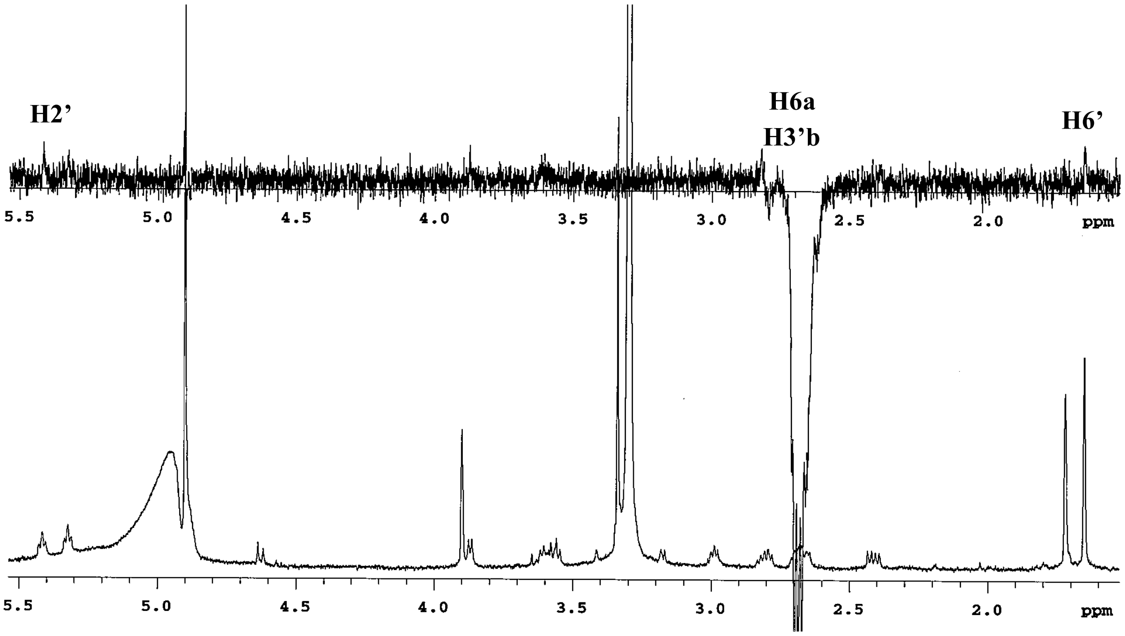

Figure S55. NOESY1D spectrum of **5** (CD_3OD, 600 MHz). Irradiated at δ2.67 ppm (H3'b, H6a) and HDO.

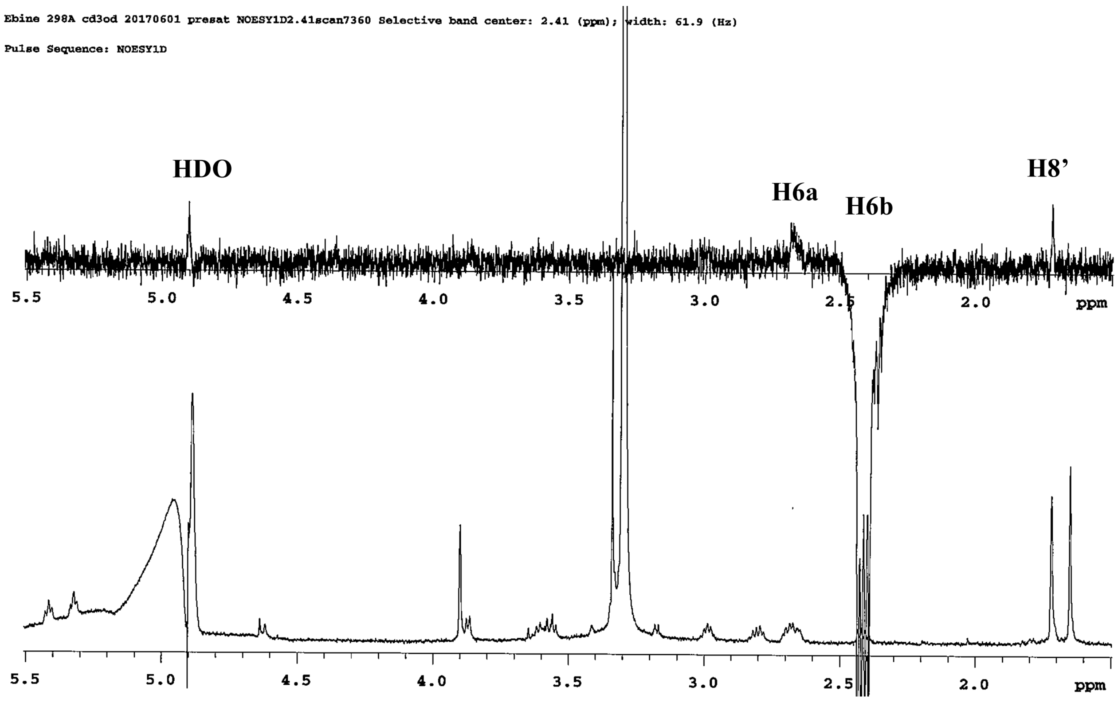

Figure S56. NOESY1D spectrum of **5** (CD_3OD, 600 MHz). Irradiated at δ2.42 ppm (H6b) and HDO.

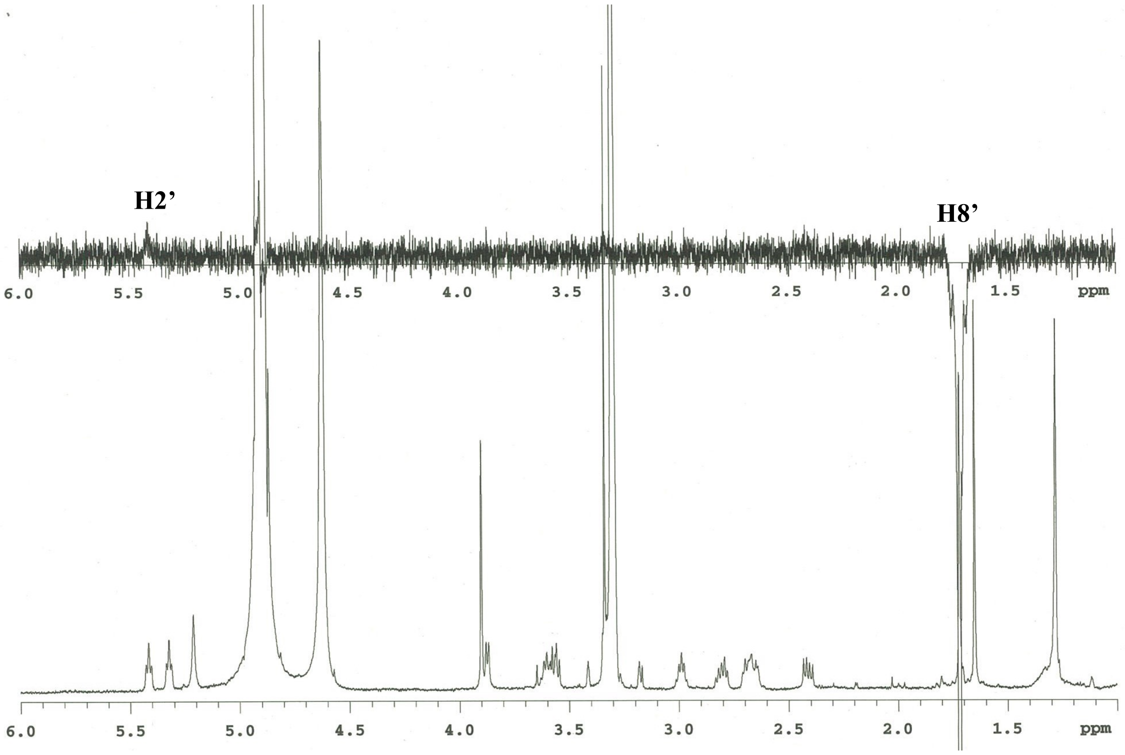

Figure S57. NOESY1D spectrum of **5** (CD_3OD, 600 MHz). Irradiated at δ1.72 ppm (H8').

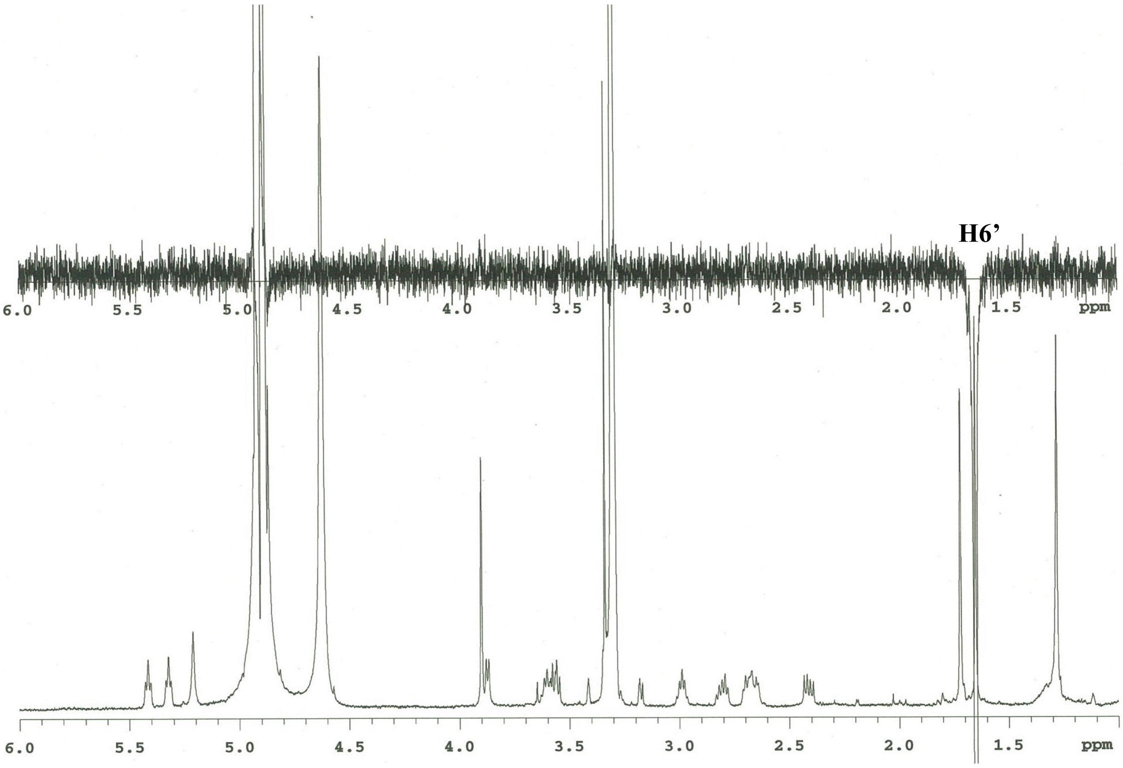

Figure S58. NOESY1D spectrum of **5** (CD_3OD, 600 MHz). Irradiated at δ1.65 ppm (H6').
