# Supplementary material for: Six domoic acid related compounds from the red alga, Chondria armata, and domoic acid biosynthesis by the diatom, Pseudo-nitzschia multiseries
Source: Sci Rep. 2018 Jan 10;8:356. doi: 10.1038/s41598-017-18651-w (PMC5762911; doi:10.1038/s41598-017-18651-w)
Supplement: Supplementary file 2 — Supplementary Information Part2 (2/2) [file 41598_2017_18651_MOESM2_ESM.pdf]

Supplementary Information Part 2 (2/2) page 31-53 (Figures S59-S99)

Six domoic acid related compounds from the red alga, *Chondria armata,* and domoic acid biosynthesis by the diatom, *Pseudo-nitzschia multiseries*

Yukari Maeno[1], Yuichi Kotaki[2], Ryuta Terada[3], Yuko Cho[1], Keiichi Konoki[1] and Mari Yotsu-Yamashita[1,]*

[1]Graduate School of Agricultural Science, Tohoku University, 468-1 Aramaki-Aza-Aoba, Aoba-ku, Sendai 980-0845, Japan

[2]Fukushima College, 1-1 Chigoike Miyashiro, Fukushima 960-0181, Japan

[3]United Graduate School of Agricultural Science, Kagoshima University, 1-21-24, Korimoto, Kagoshima 890-0065, Japan

・NMR spectra of 7’-hydroxymethyl-isodomoic acid B (**6**)

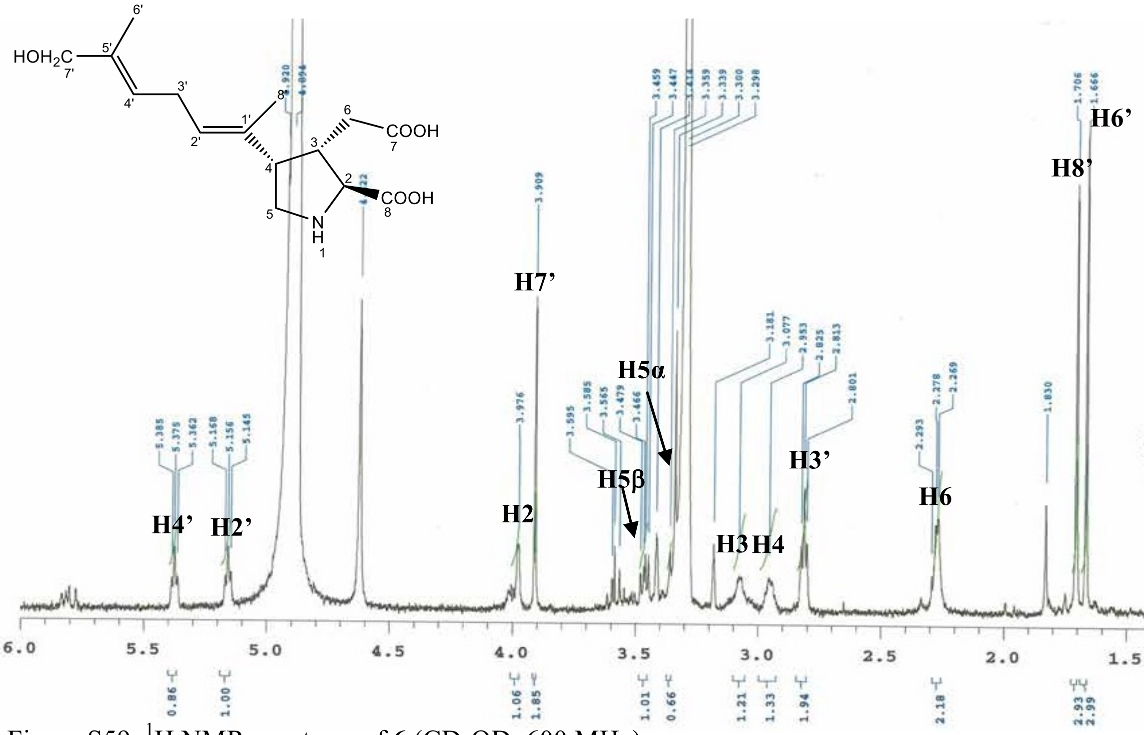

Figure S59. ^{1}H NMR spectrum of **6** (CD_3OD, 600 MHz).

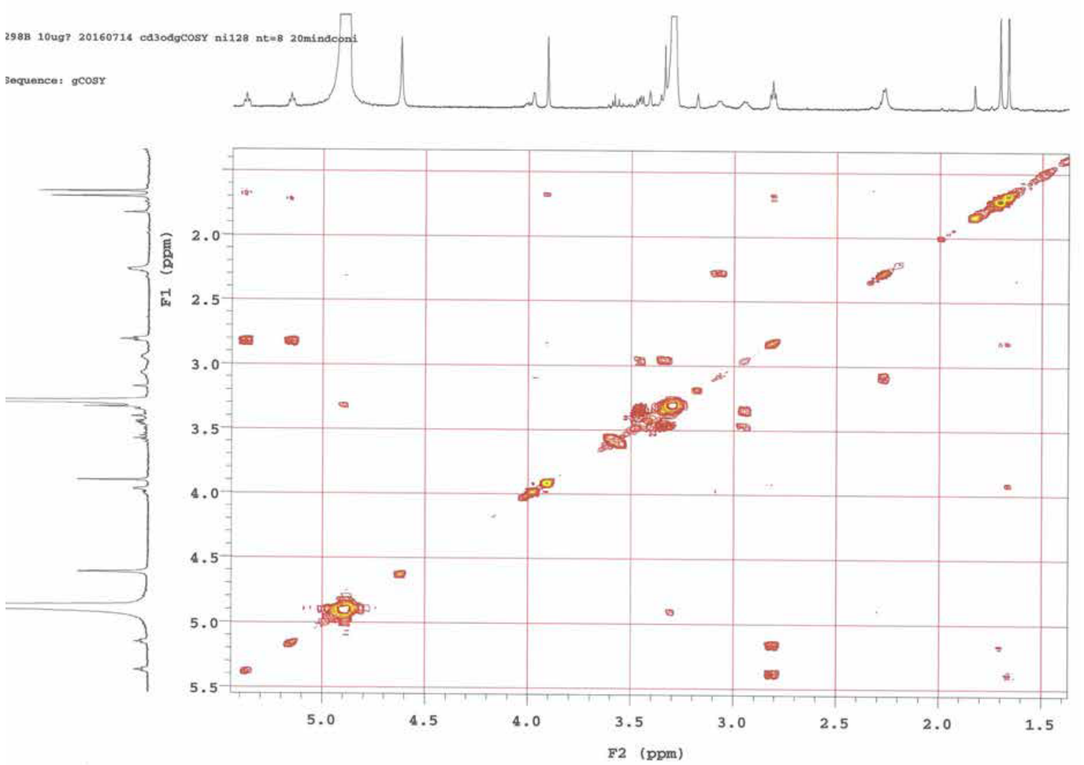

Figure S60. Gradient COSY spectrum of **6** (CD_3OD, 600 MHz).

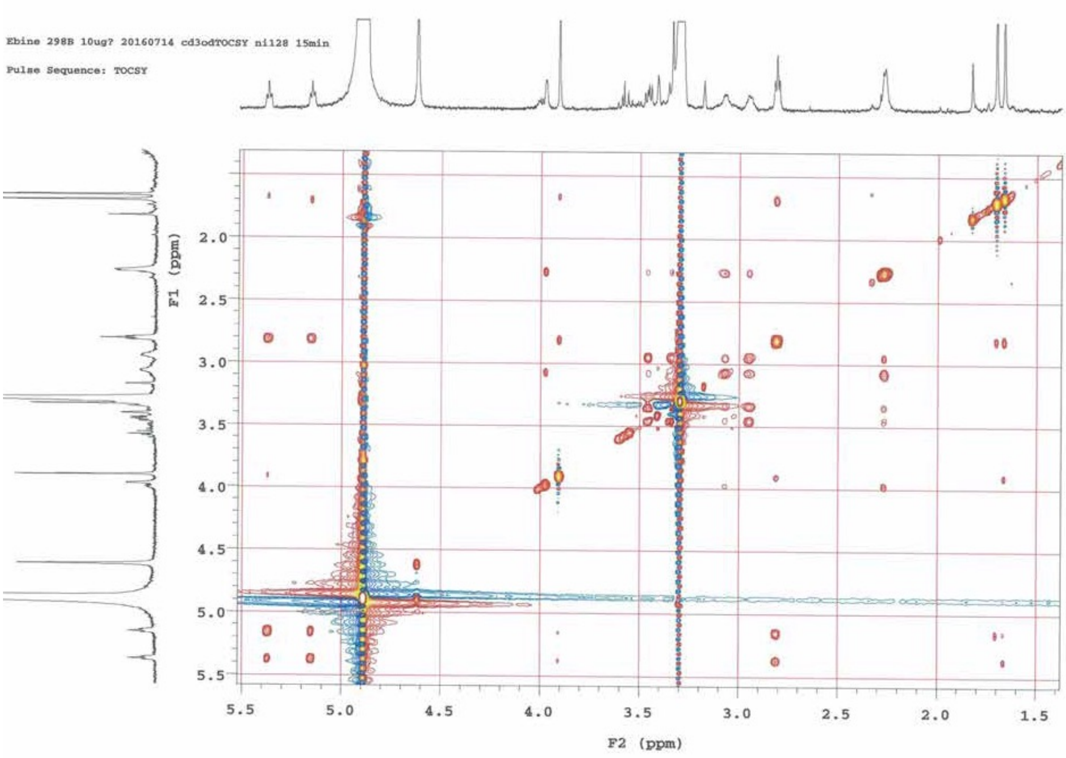

Figure S61. TOCSY spectrum of **6** (CD_3OD, 600 MHz).

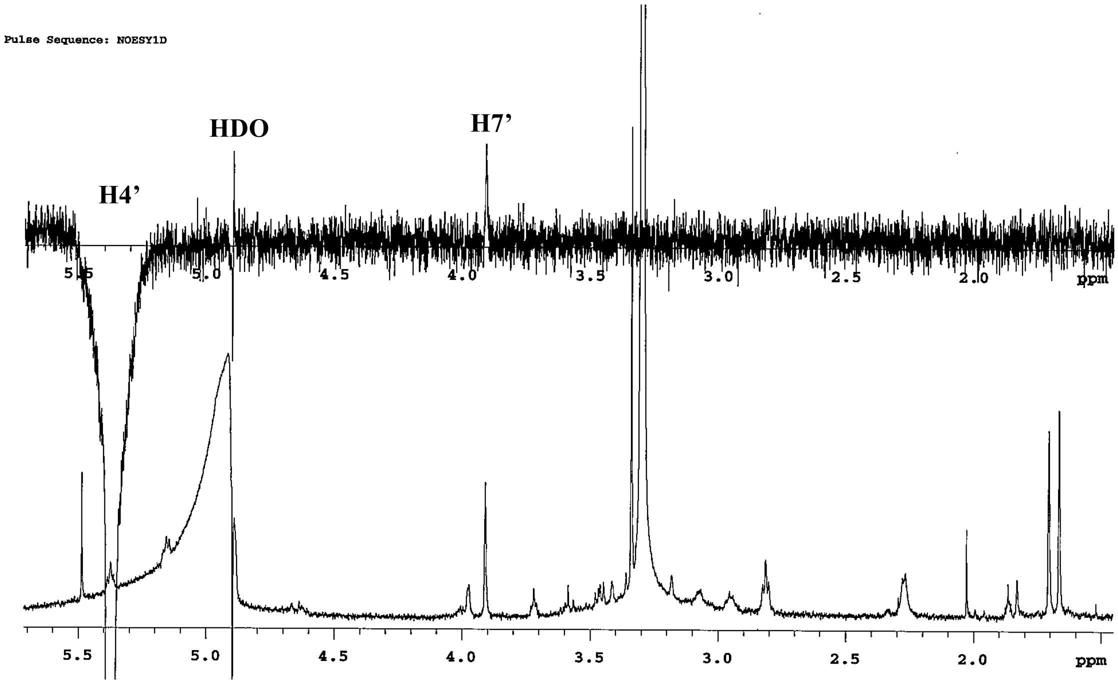

Figure S62. NOESY1D spectrum of **6** (CD_3OD, 600 MHz). Irradiated at δ5.38 ppm (H4’) and HDO.

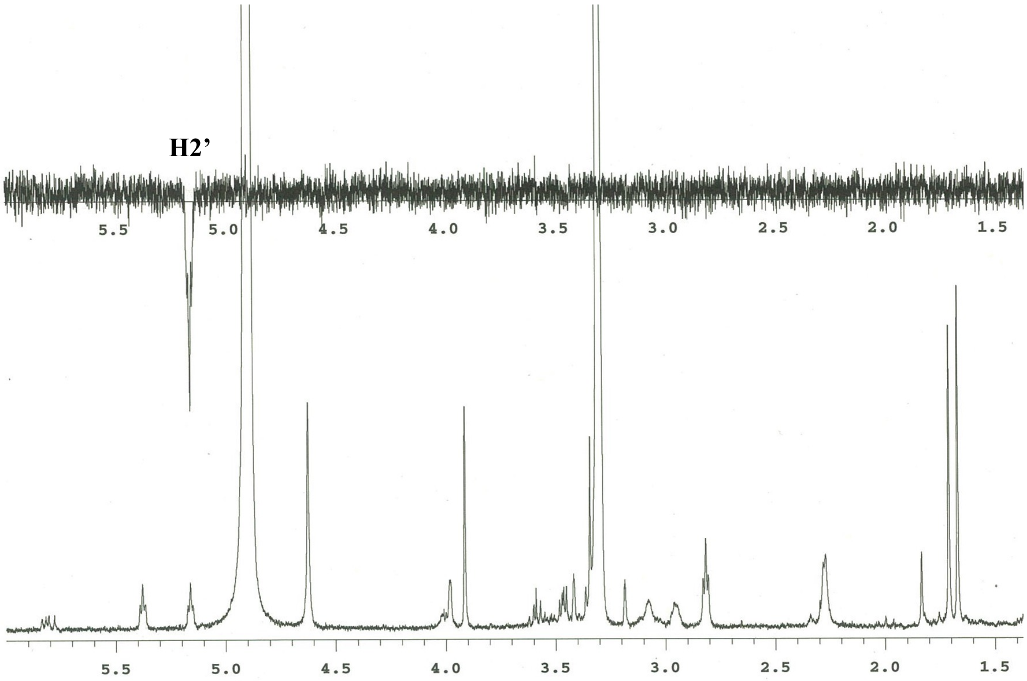

Figure S63. NOESY1D spectrum of **6** (CD_3OD, 600 MHz). Irradiated at δ5.16 ppm (H2’).

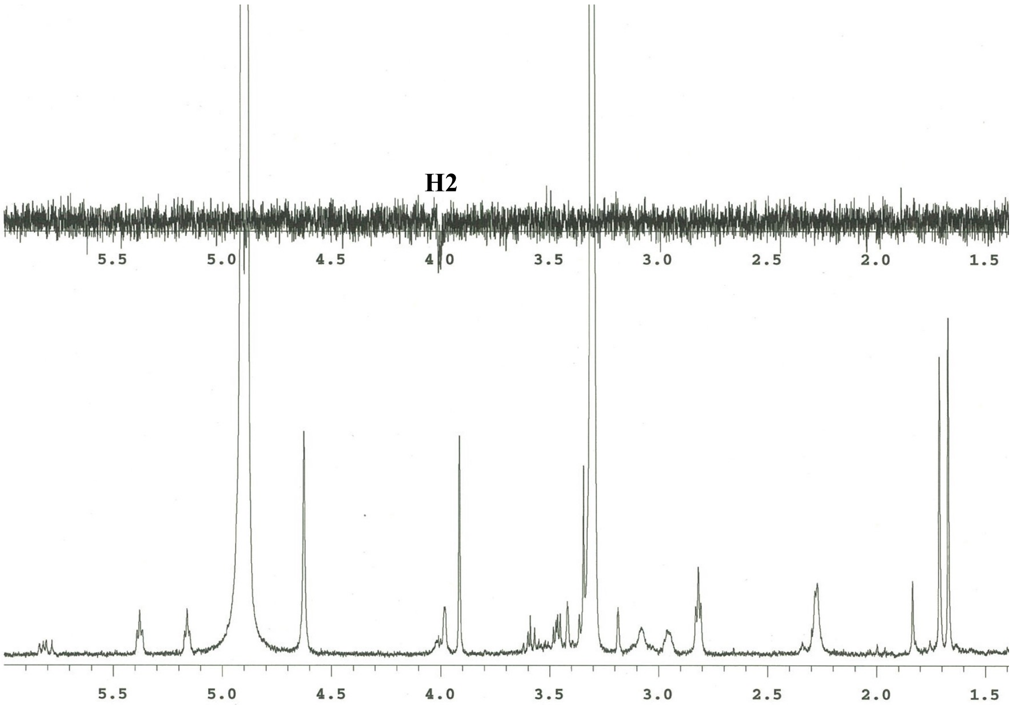

Figure S64. NOESY1D spectrum of **6** (CD_3OD, 600 MHz). Irradiated at δ3.91 ppm (H2).

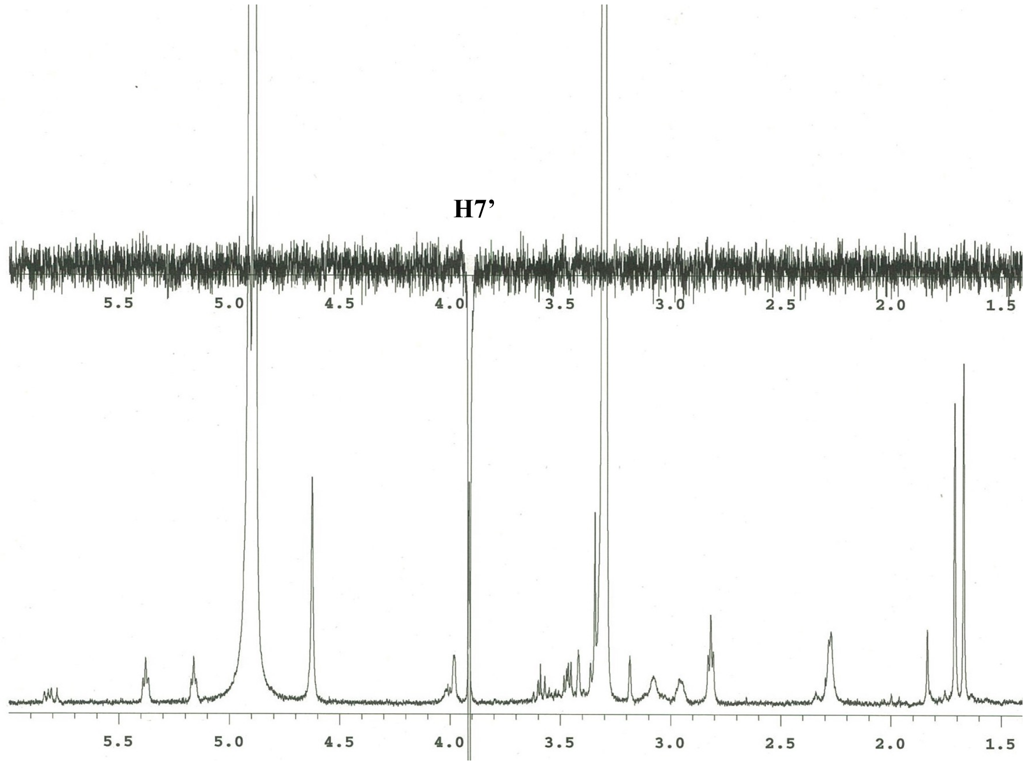

Figure S65. NOESY1D spectrum of **6** (CD_3OD, 600 MHz). Irradiated at δ3.91 ppm (H7').

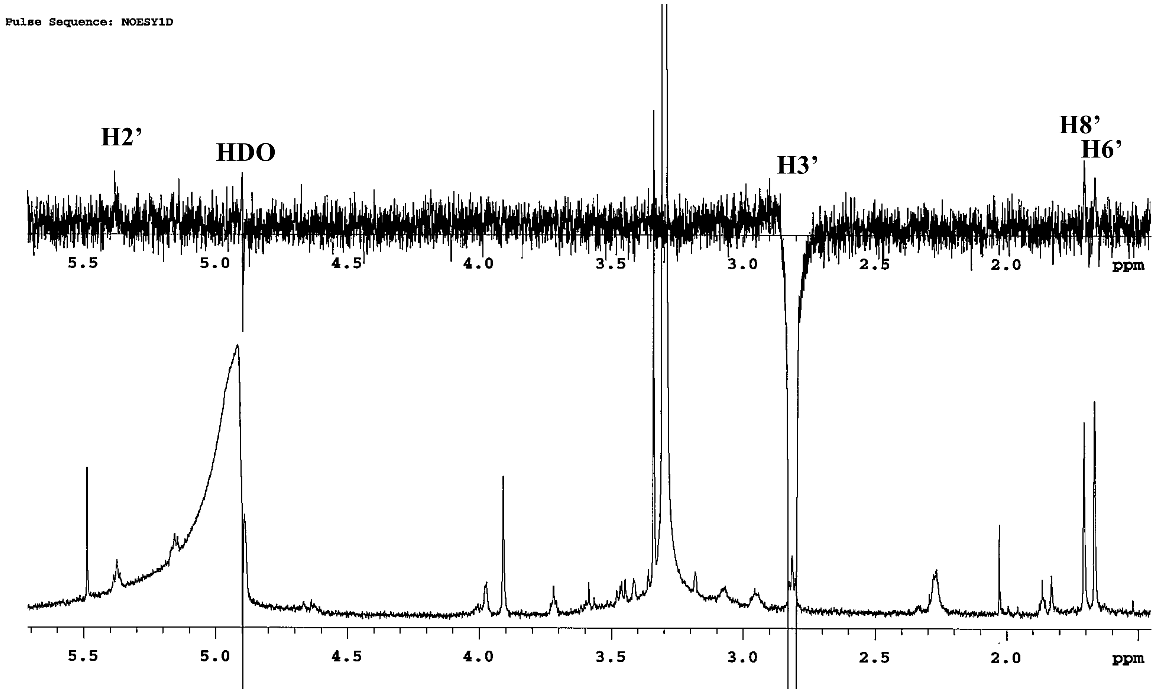

Figure S66. NOESY1D spectrum of **6** (CD_3OD, 600 MHz). Irradiated at δ2.82 ppm (H3') and HDO.

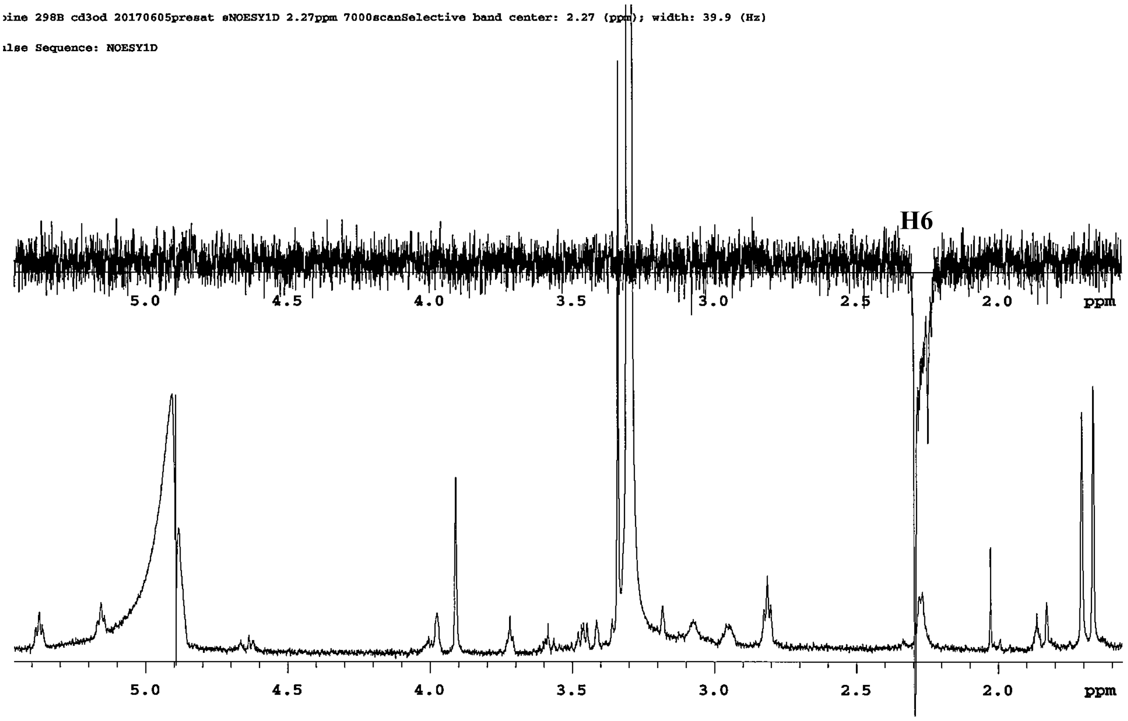

Figure S67. NOESY1D spectrum of **6** (CD_3OD, 600 MHz). Irradiated at δ2.28 ppm (H6) and HDO.

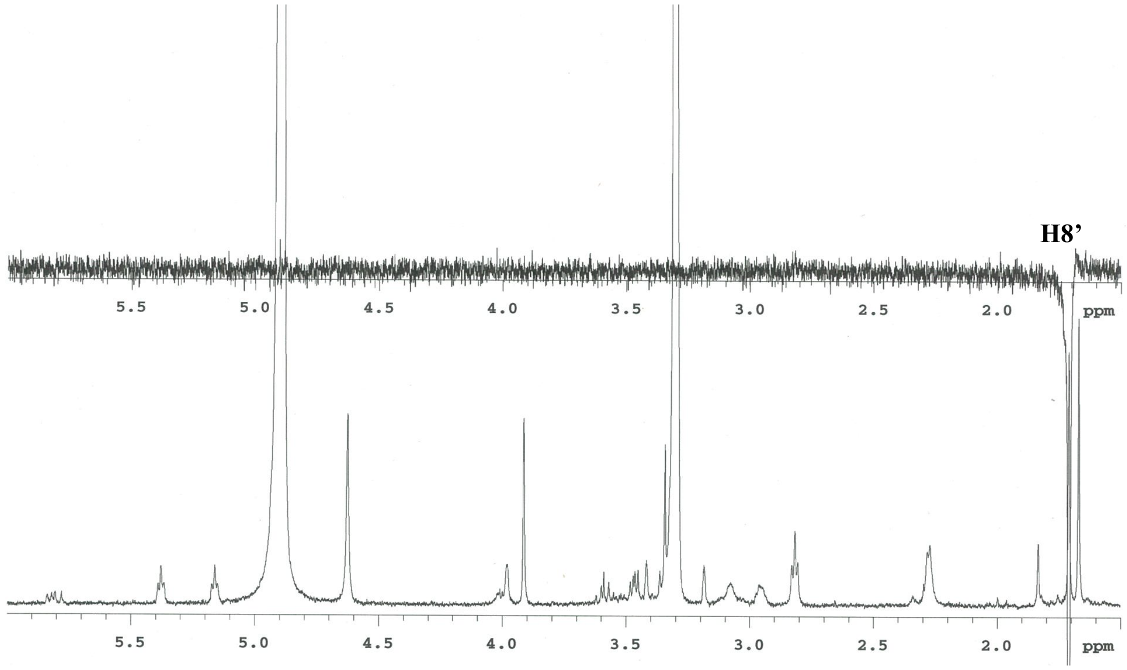

Figure S68. NOESY1D spectrum of **6** (CD_3OD, 600 MHz). Irradiated at δ1.71 ppm (H8').

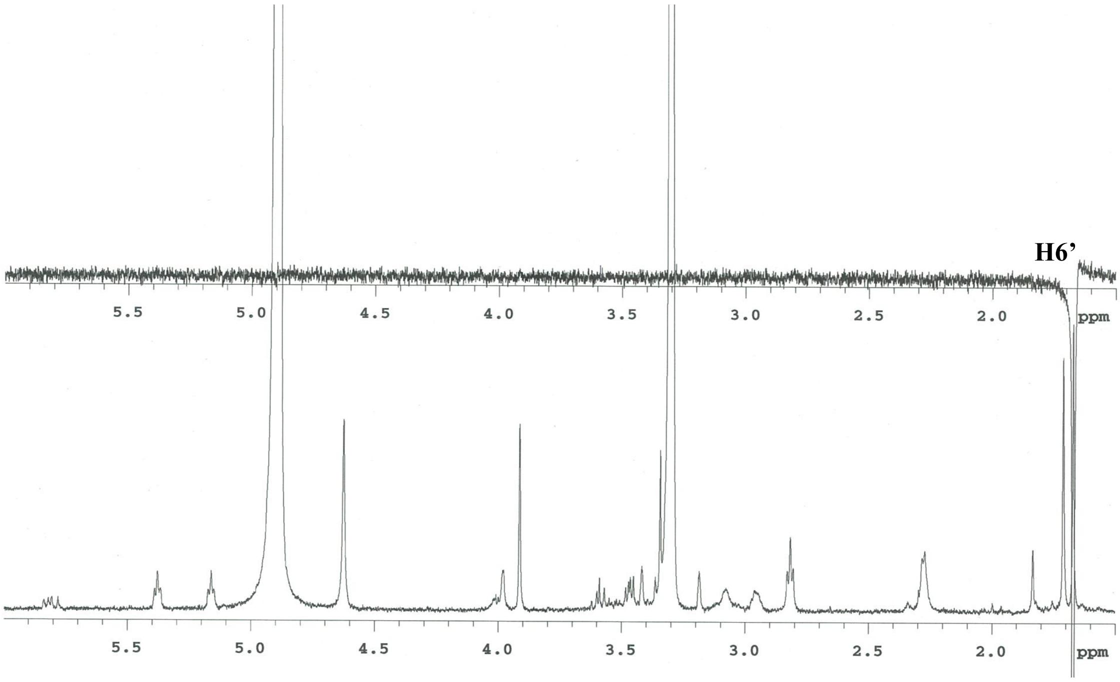

Figure S69. NOESY1D spectrum of **6** (CD_3OD, 600 MHz). Irradiated at δ1.67 ppm (H6’).

・NMR spectra of *N*-geranyl-3(*R*)-hydroxy-L-glutamic acid (**7**).

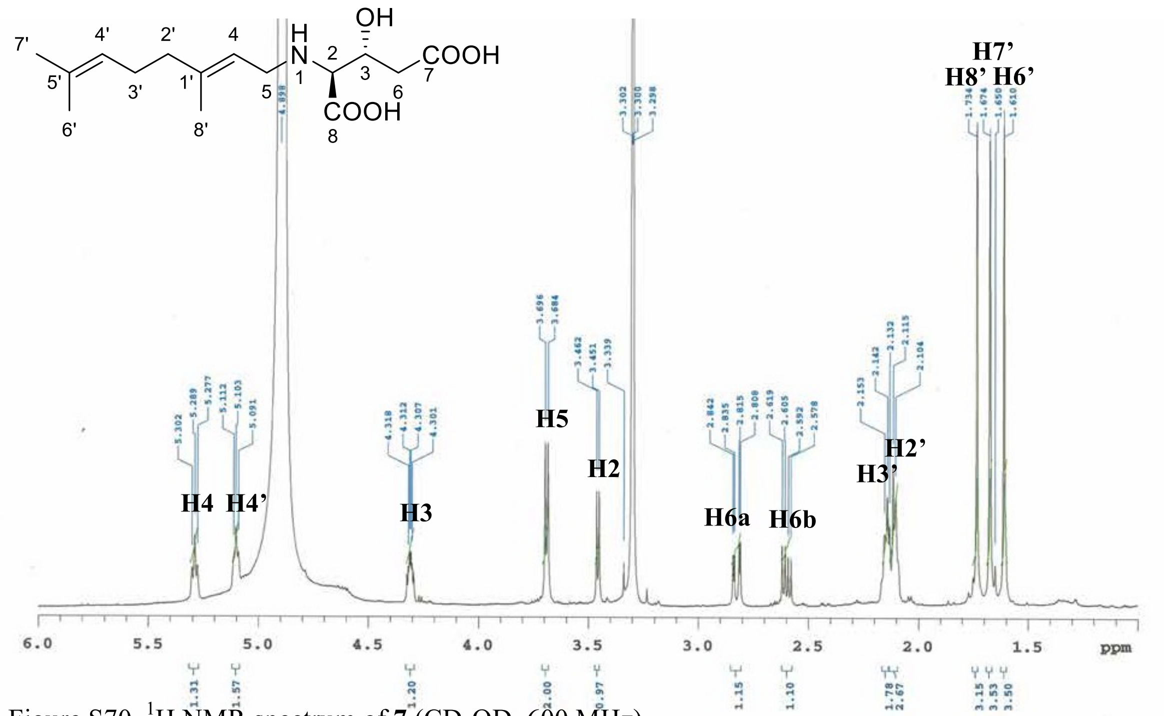

Figure S70. ^{1}H NMR spectrum of **7** (CD_3OD, 600 MHz).

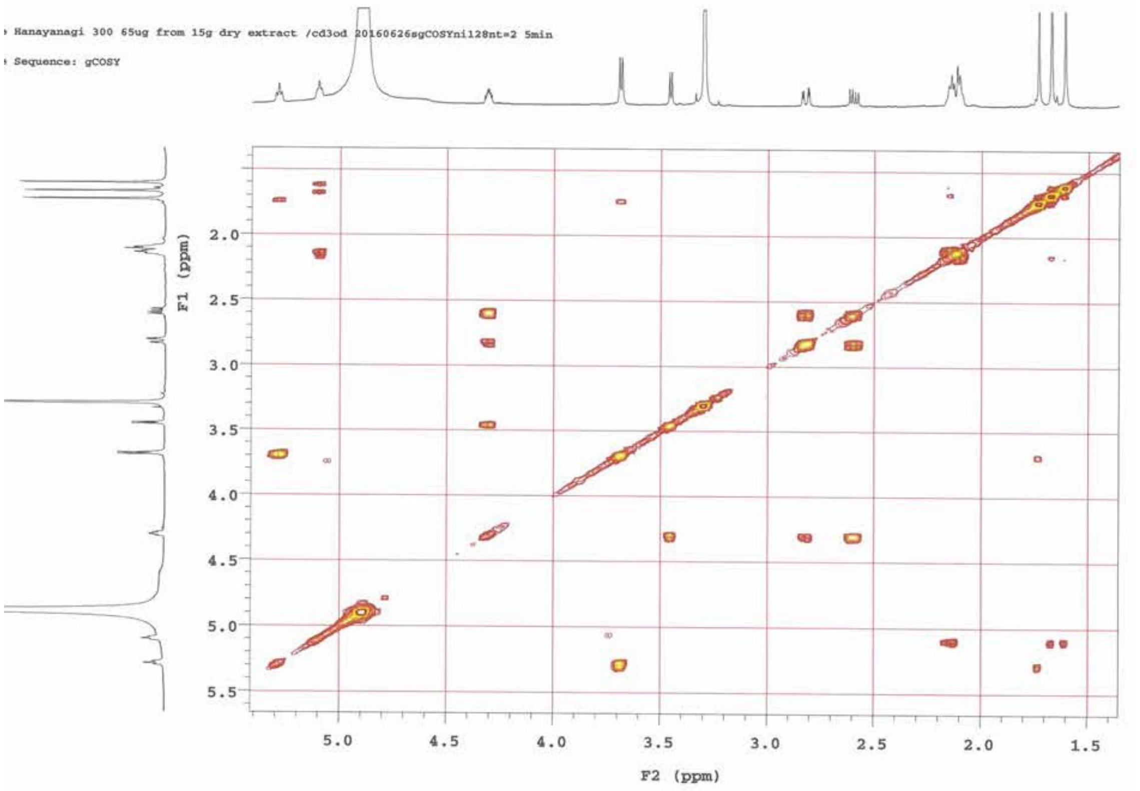

Figure S71. Gradient COSY spectrum of **7** (CD_3OD, 600 MHz).

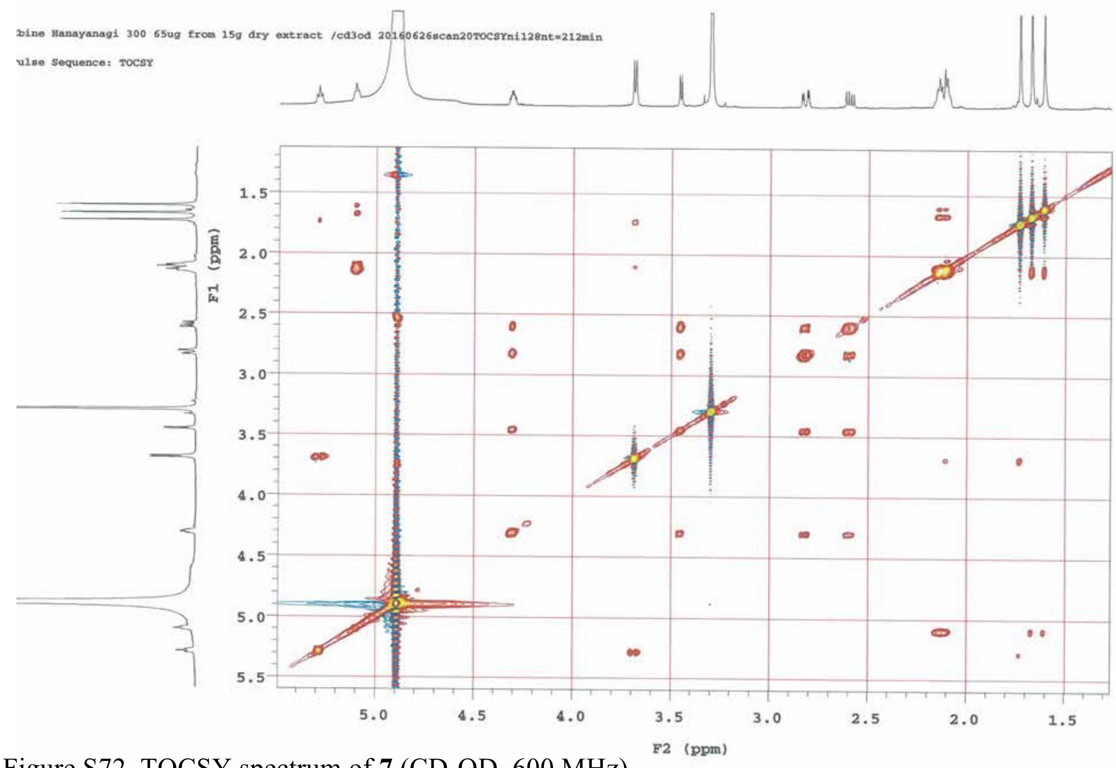

Figure S72. TOCSY spectrum of **7** (CD_3OD, 600 MHz).

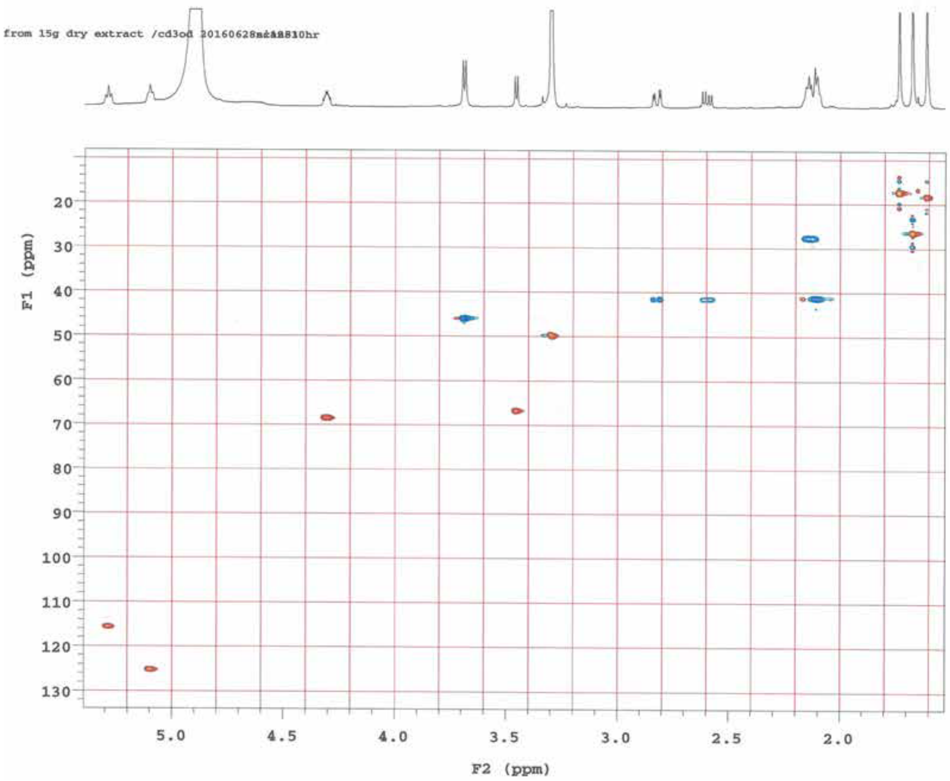

Figure S73. Gradient HSQC spectrum of **7** (CD_3OD, 600 MHz).

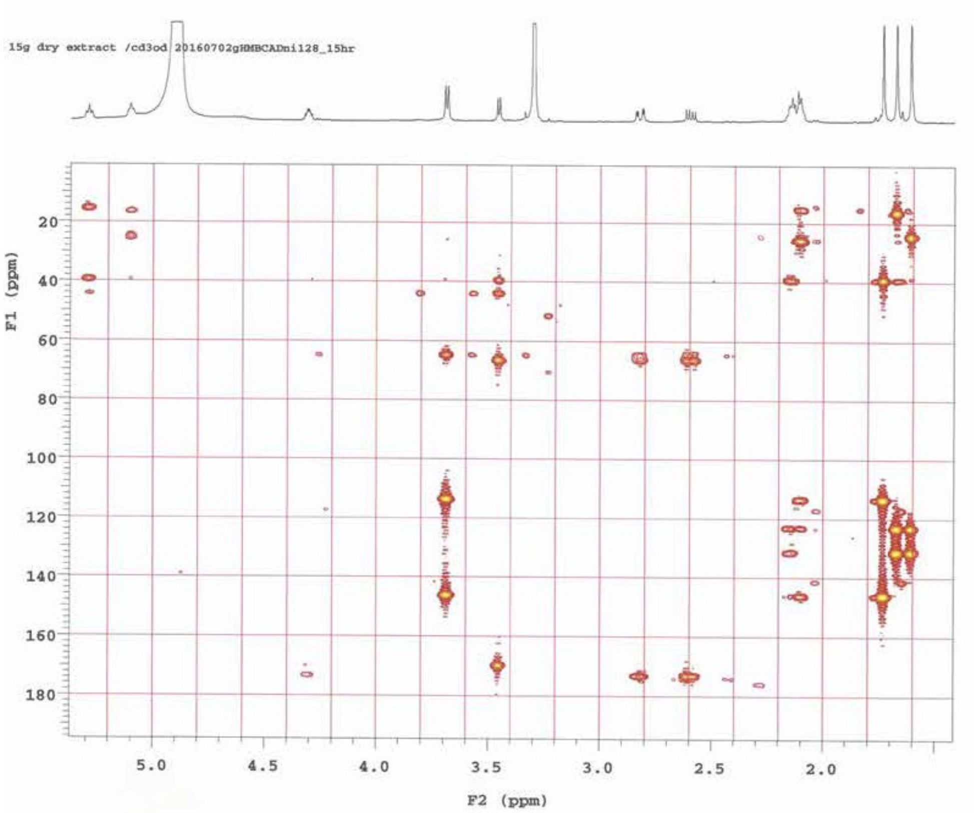

Figure S74. Gradient HMBC spectrum of **7** (CD_3OD, 600 MHz).

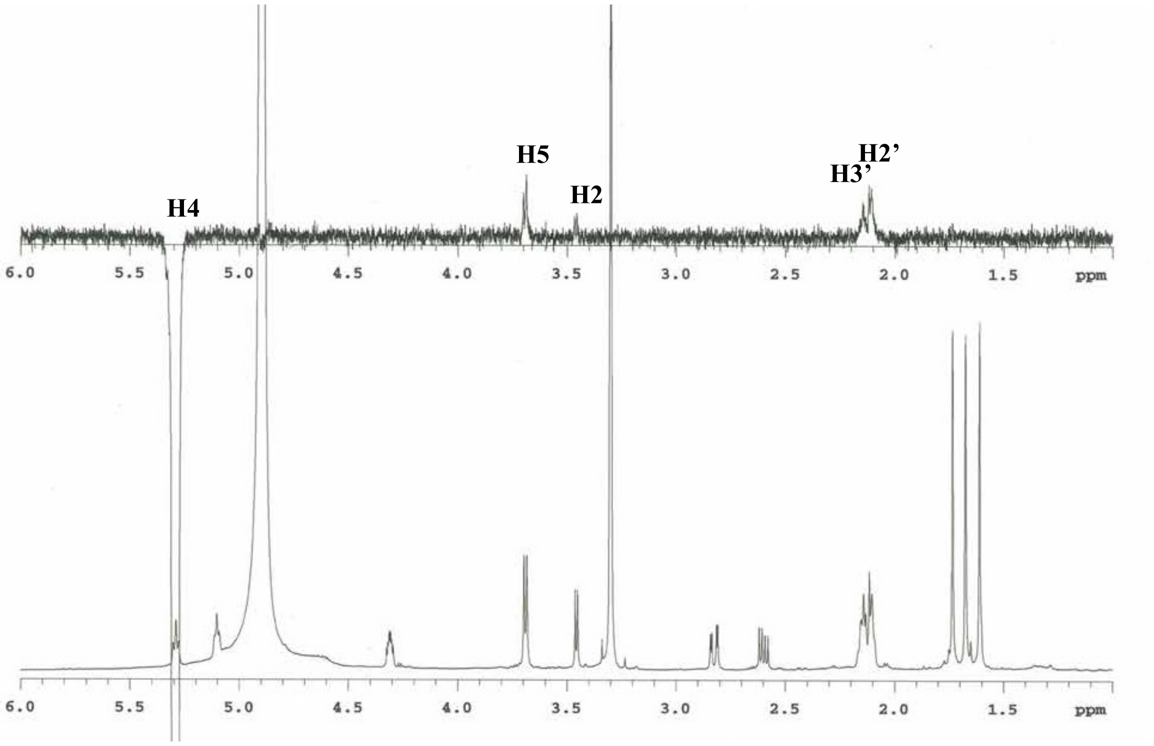

Figure S75. NOESY1D spectrum of **7** (CD_3OD, 600 MHz). Irradiated at δ5.14 ppm (H4).

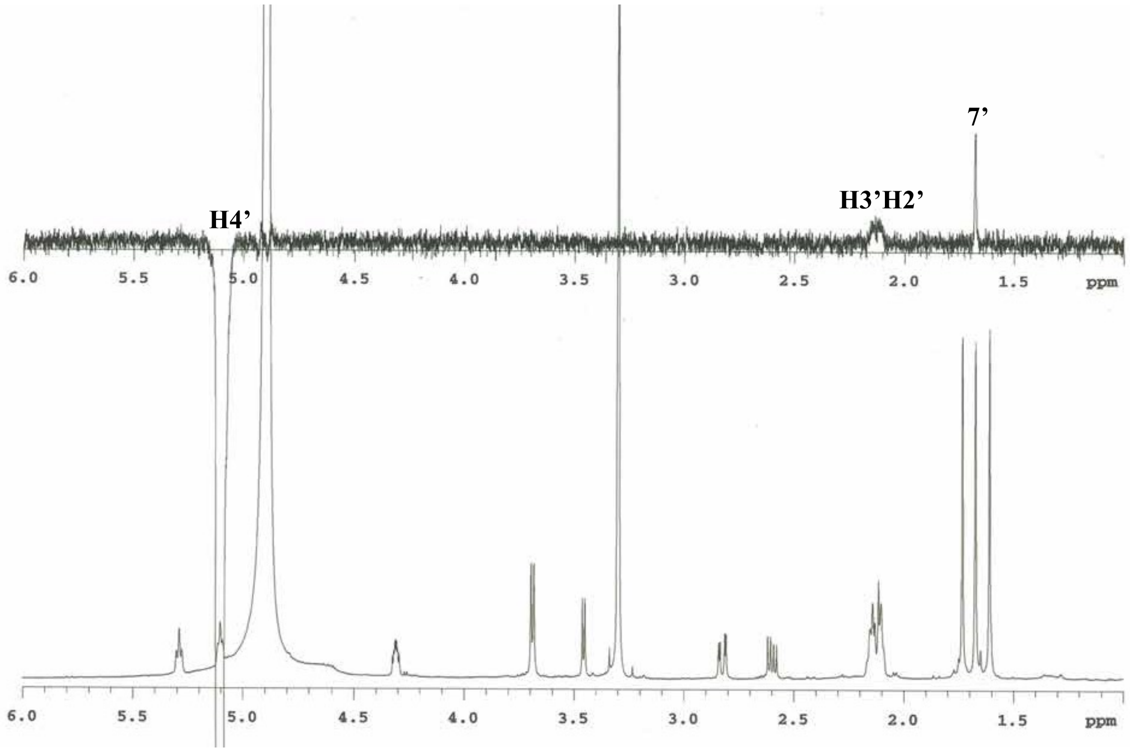

Figure S76. NOESY1D spectrum of **7** (CD_3OD, 600 MHz). Irradiated at δ5.14 ppm (H4’).

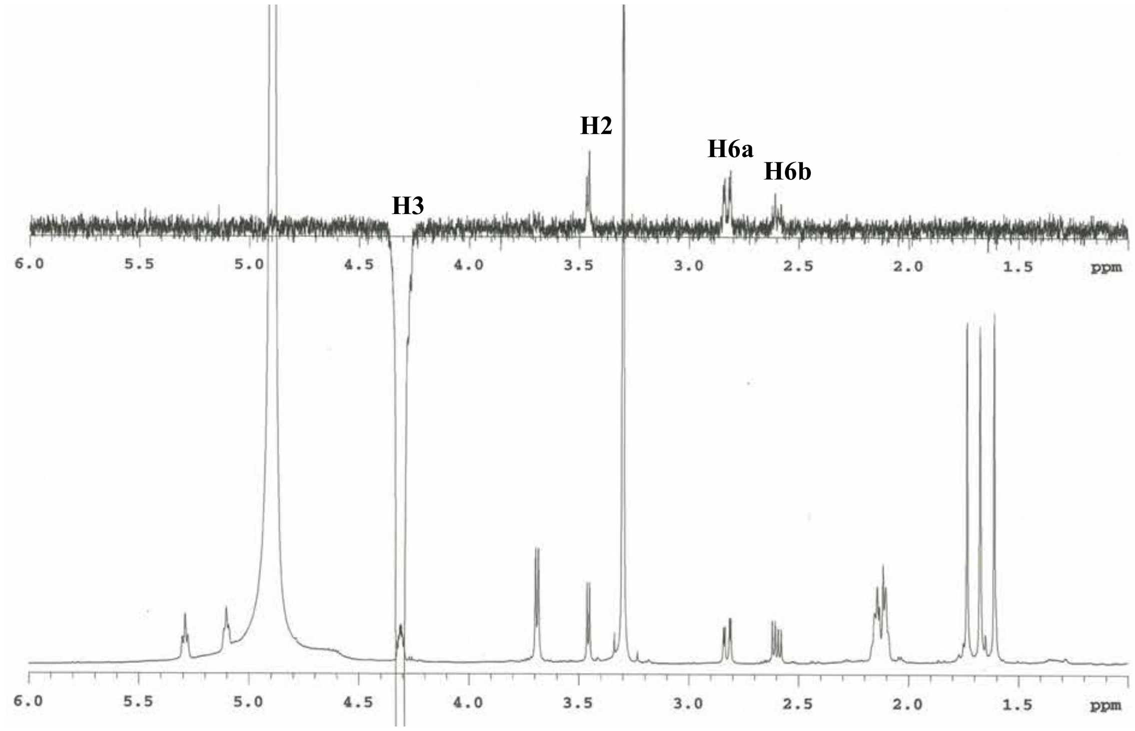

Figure S77. NOESY1D spectrum of **7** (CD_3OD, 600 MHz). Irradiated at δ5.14 ppm (H3).

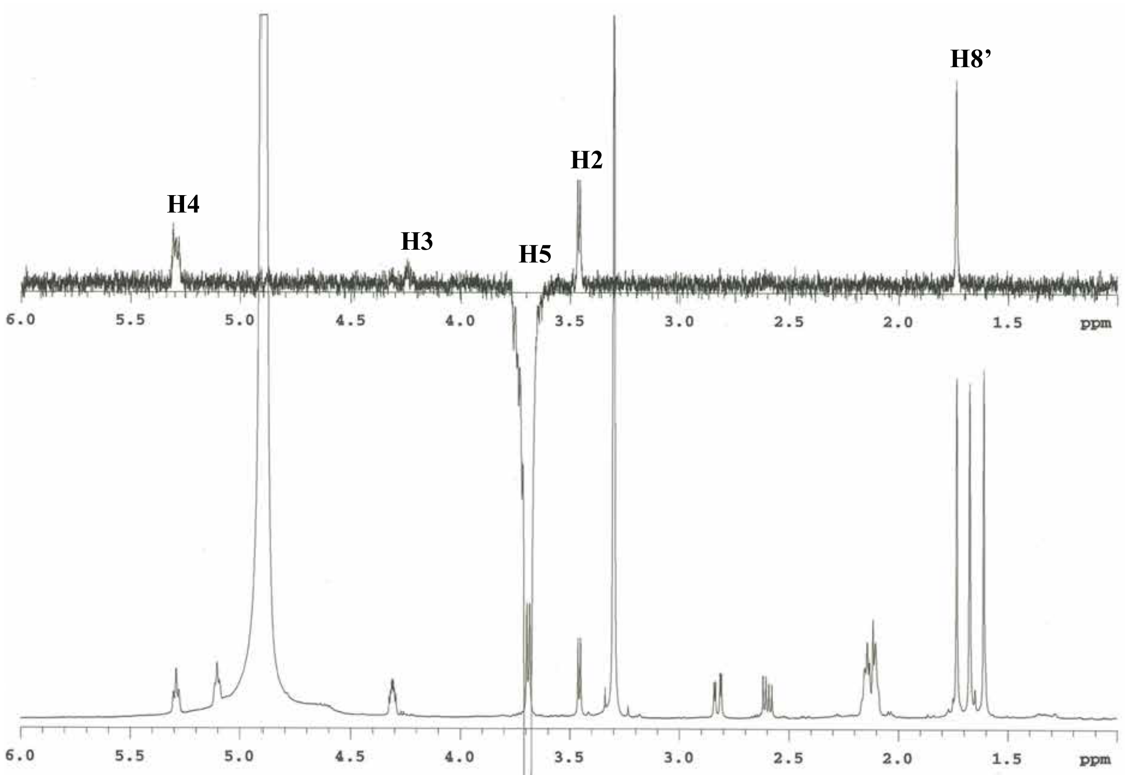

Figure S78. NOESY1D spectrum of **7** (CD_3OD, 600 MHz). Irradiated at δ5.14 ppm (H5).

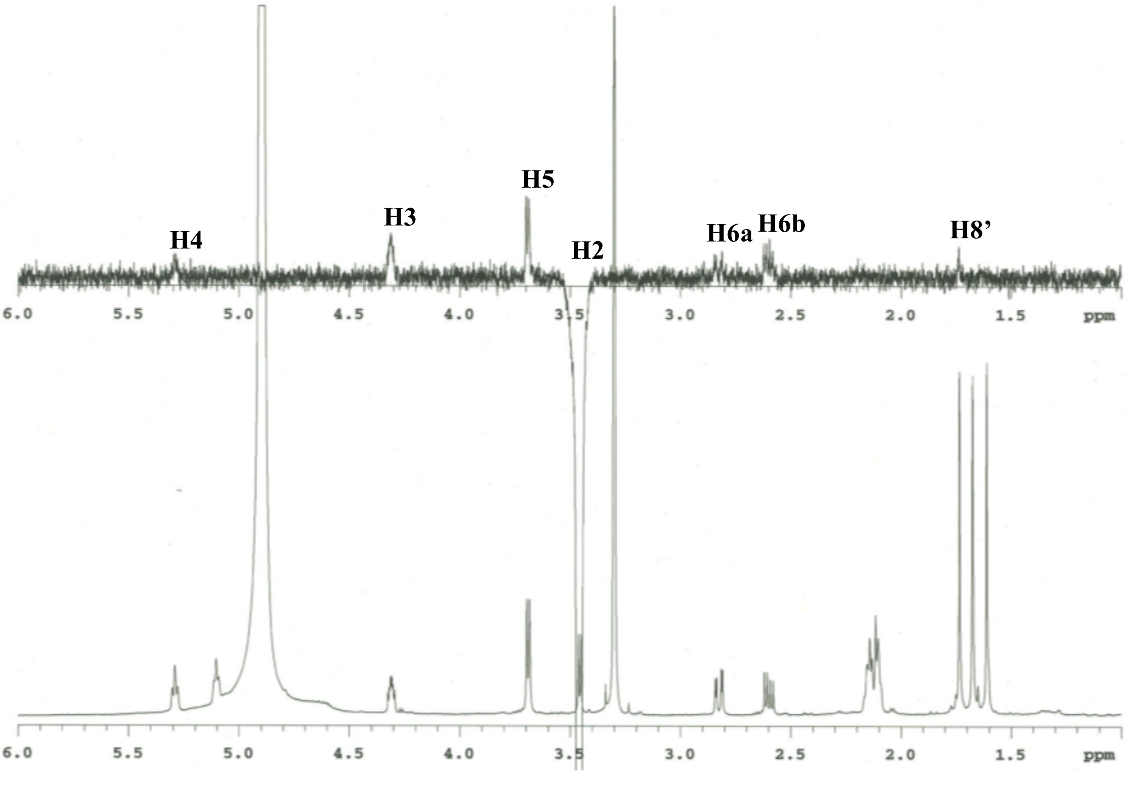

Figure S79. NOESY1D spectrum of **7** (CD_3OD, 600 MHz). Irradiated at δ5.14 ppm (H2).

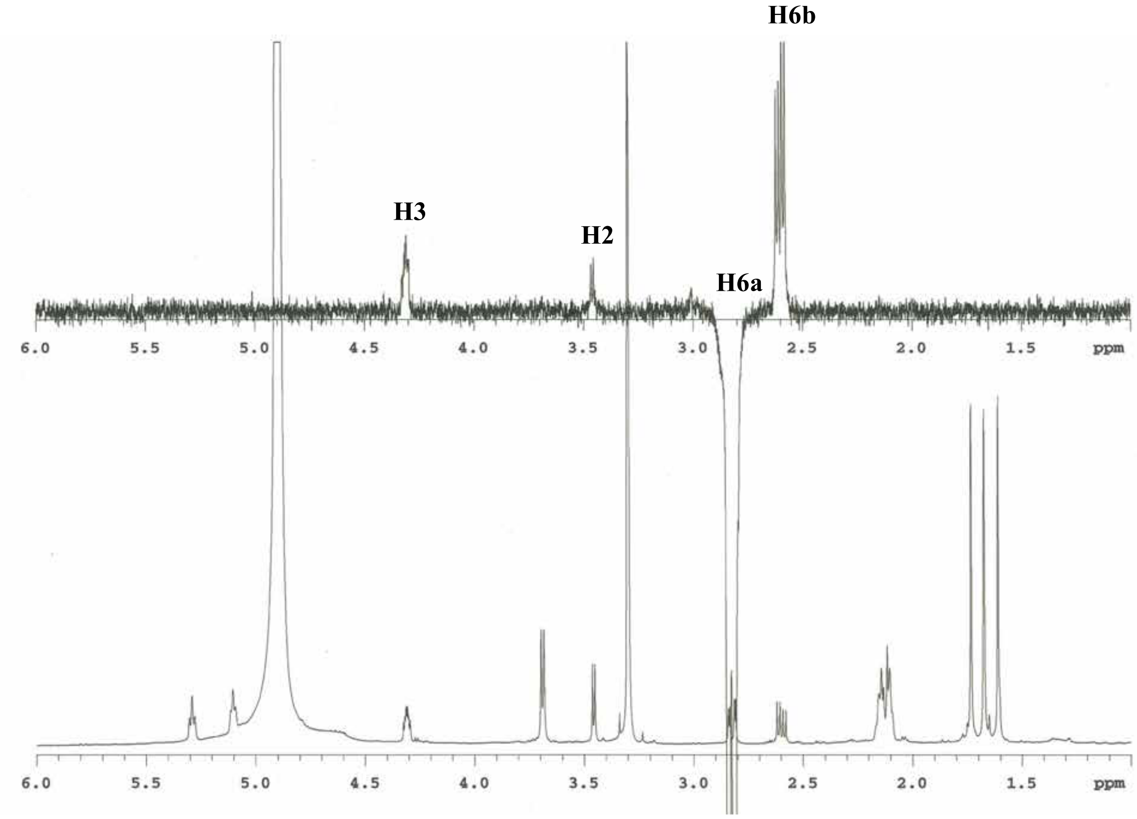

Figure S80. NOESY1D spectrum of **7** (CD_3OD, 600 MHz). Irradiated at δ5.14 ppm (H6a).

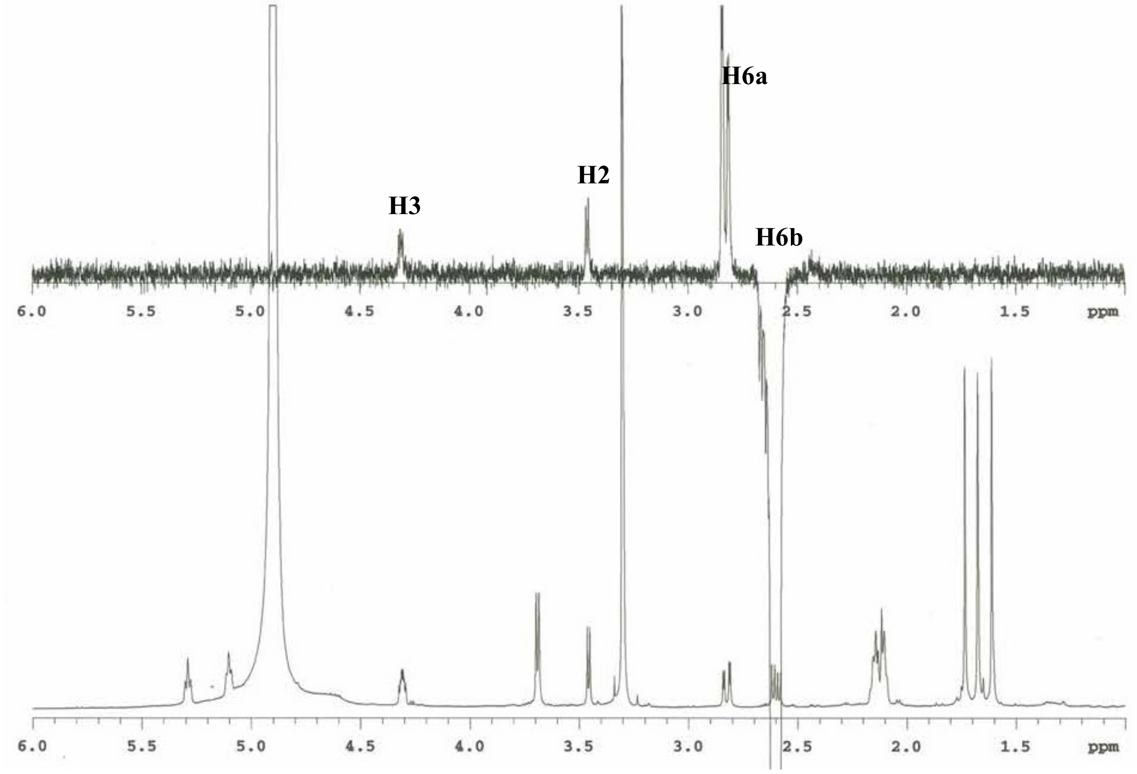

Figure S81. NOESY1D spectrum of **7** (CD_3OD, 600 MHz). Irradiated at δ5.14 ppm (H6b).

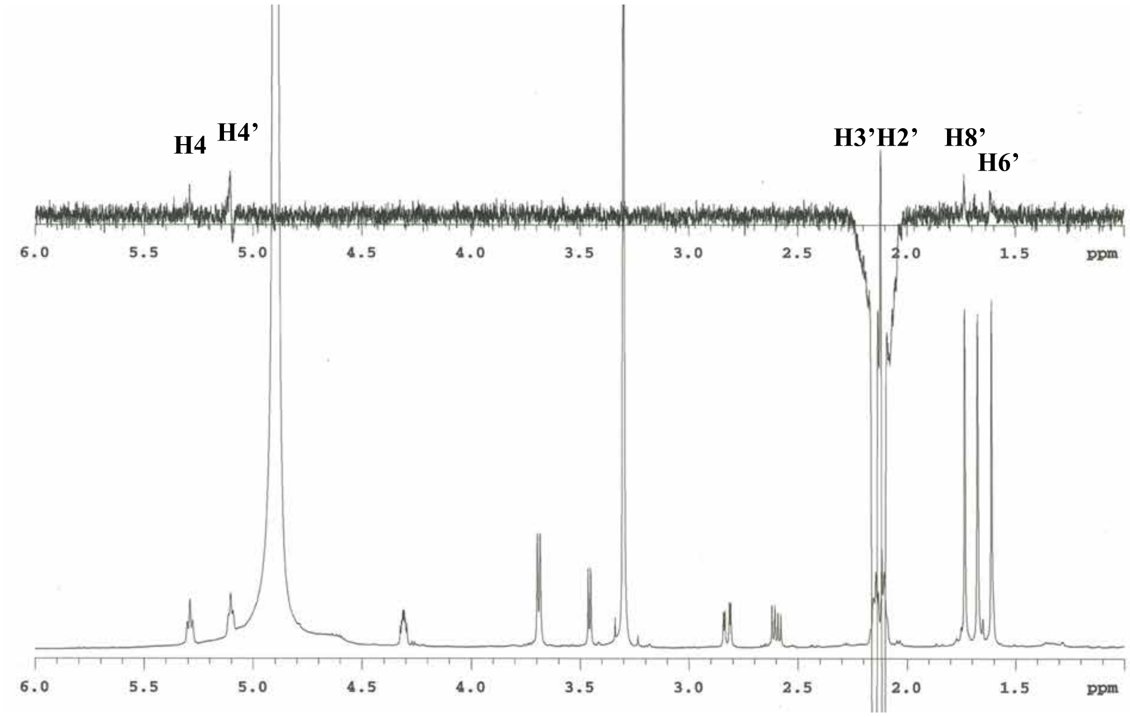

Figure S82. NOESY1D spectrum of **7** (CD_3OD, 600 MHz). Irradiated at δ5.14 ppm (H2’, 3’).

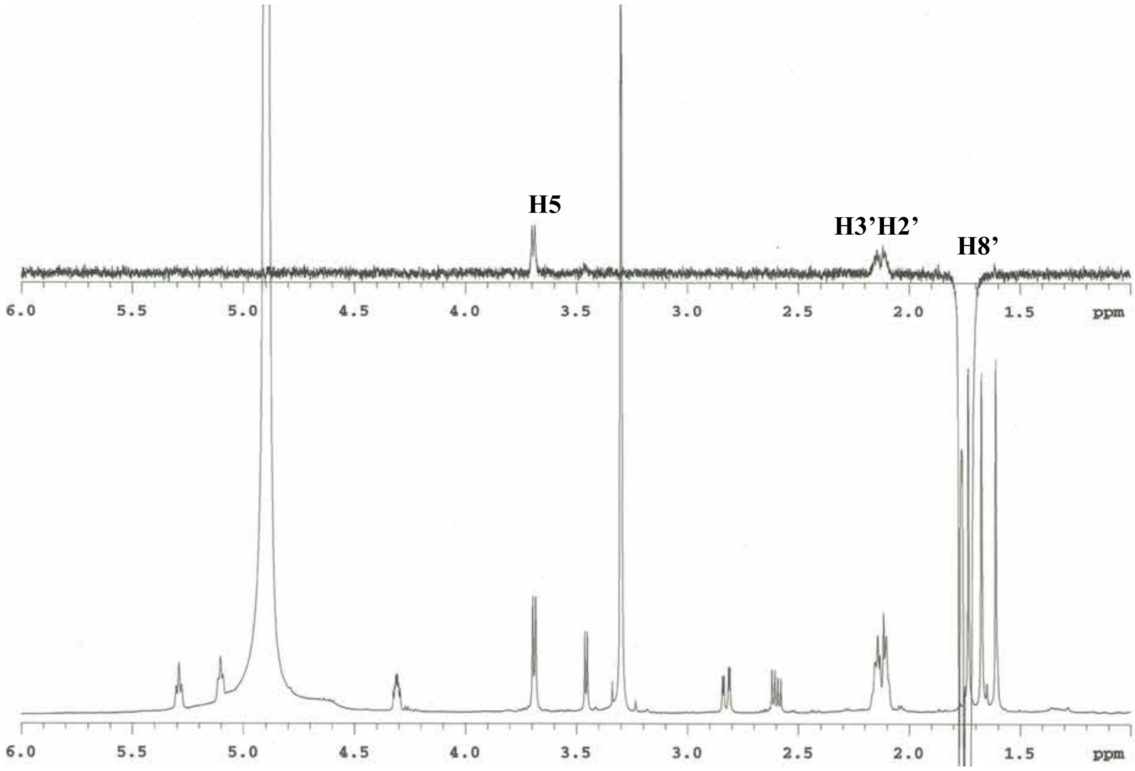

Figure S83. NOESY1D spectrum of **7** (CD_3OD, 600 MHz). Irradiated at δ5.14 ppm (H8’).

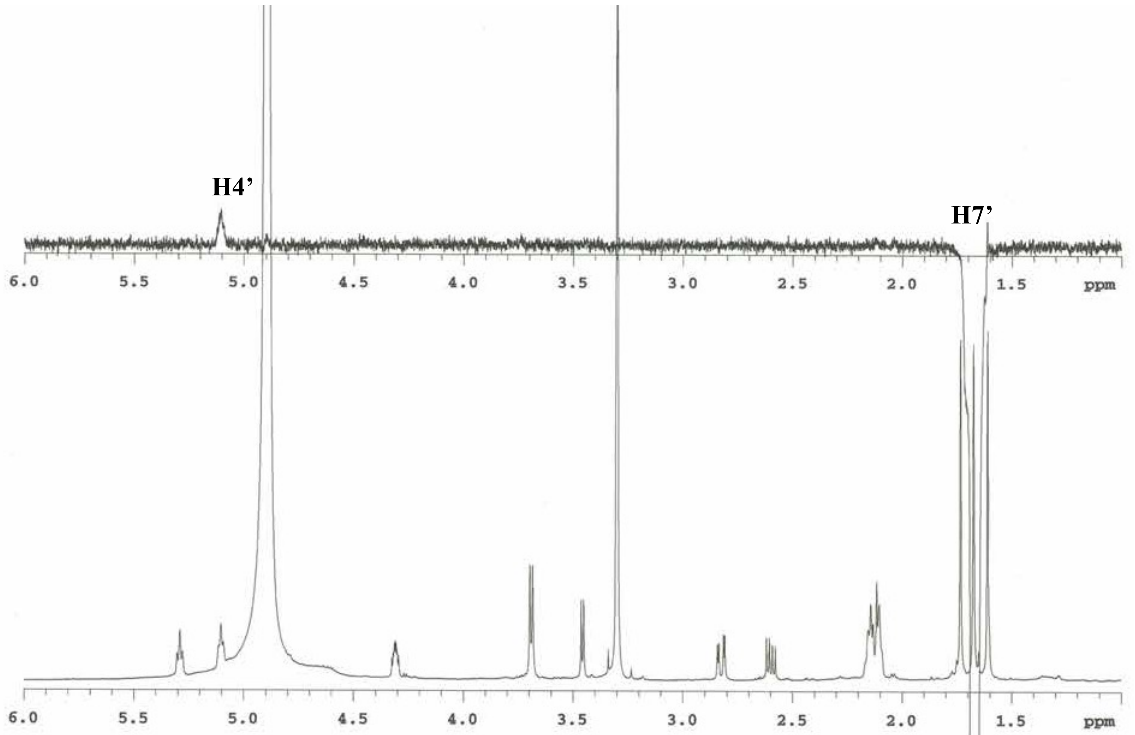

Figure S84. NOESY1D spectrum of **7** (CD_3OD, 600 MHz). Irradiated at δ5.14 ppm (H7’).

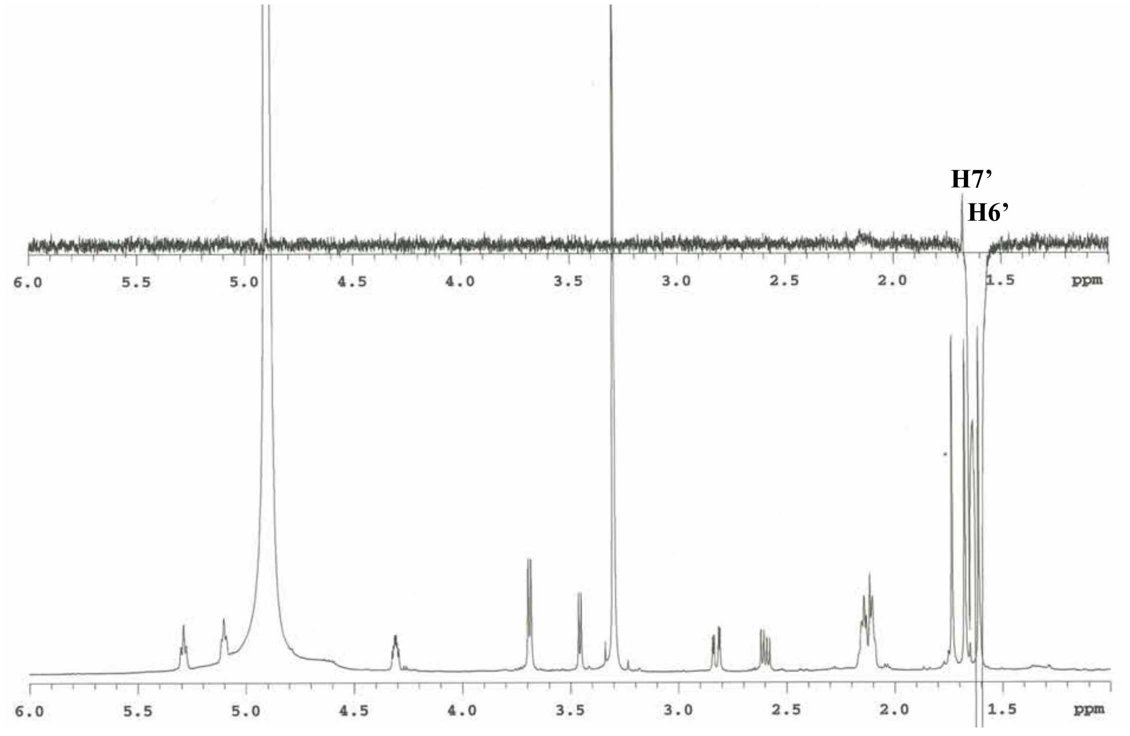

Figure S85. NOESY1D spectrum of **7** (CD_3OD, 600 MHz). Irradiated at δ5.14 ppm (H6’).

3. NMR spectra of synthetic 4 and 7.

・NMR spectra of synthetic *N*-geranyl-L-glutamic acid (**4**).

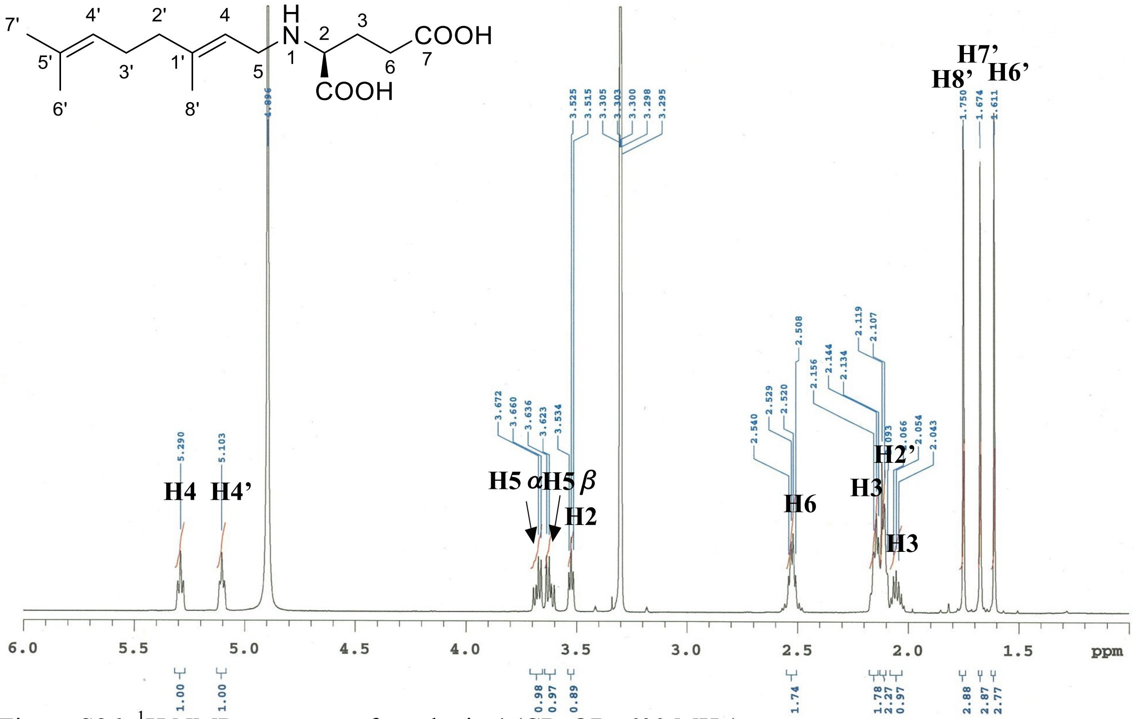

Figure S86. ^{1}H NMR spectrum of synthetic **4** (CD_3OD, 600 MHz).

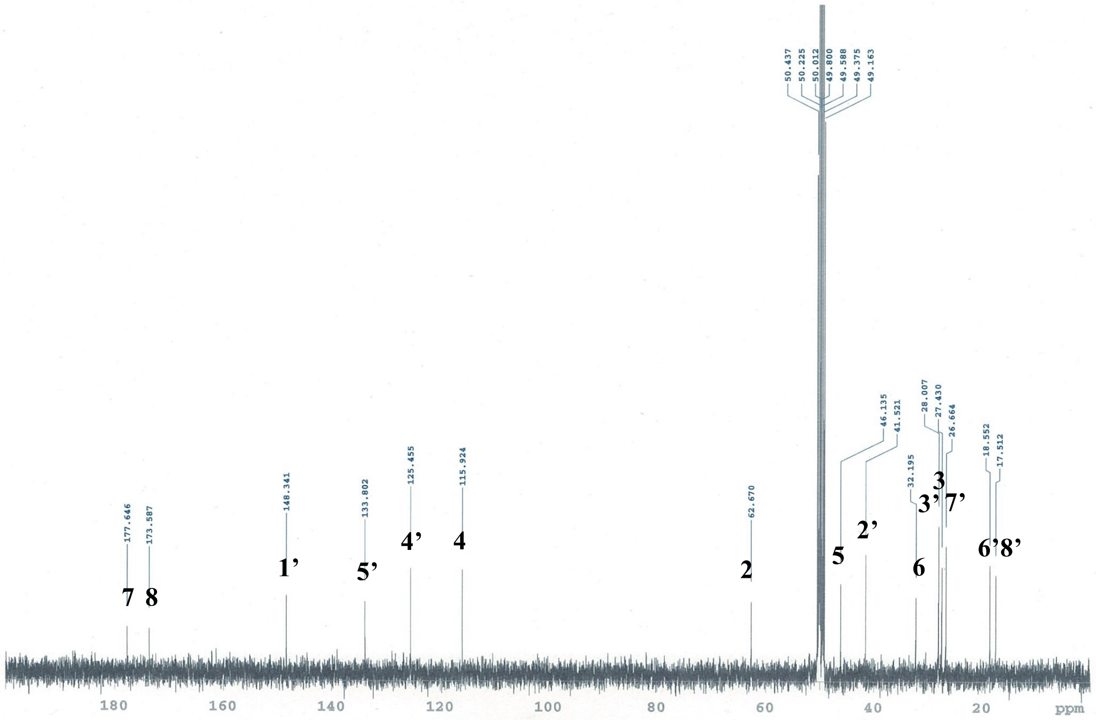

Figure S87. ^{13}C NMR spectrum of synthetic **4** (CD_3OD, 150 MHz).

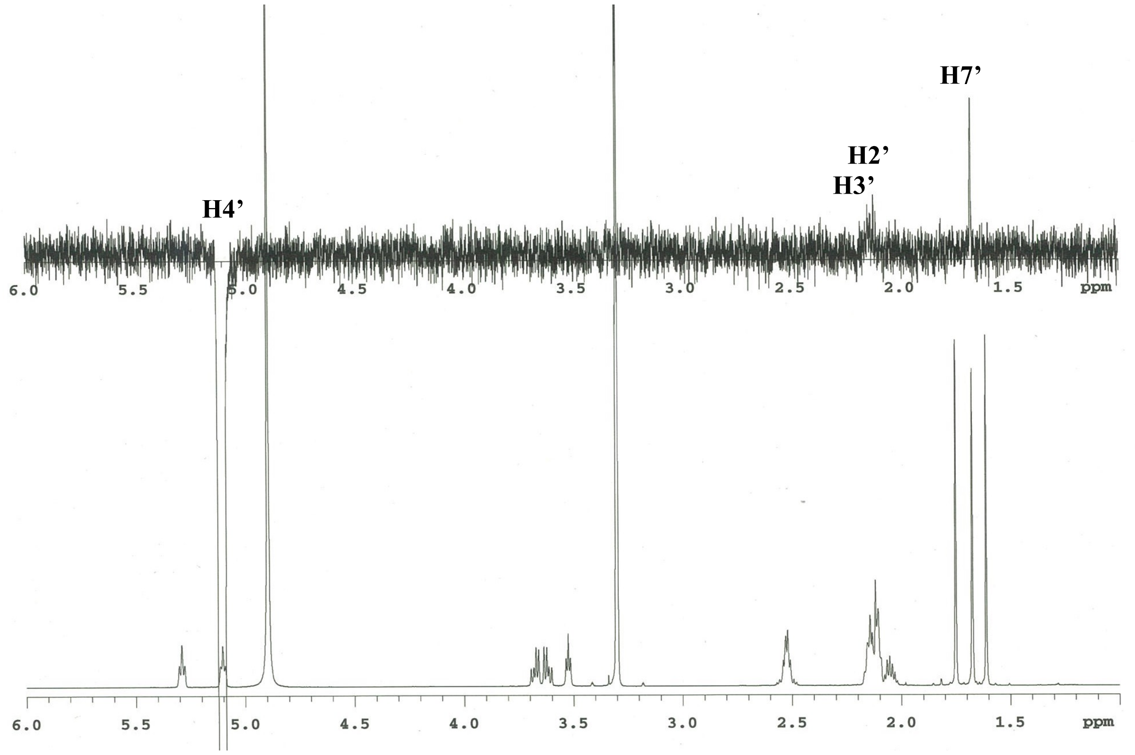

Figure S88. NOESY1D spectrum of synthetic **4** (CD_3OD, 600 MHz). Irradiated at δ5.10 ppm (H4’).

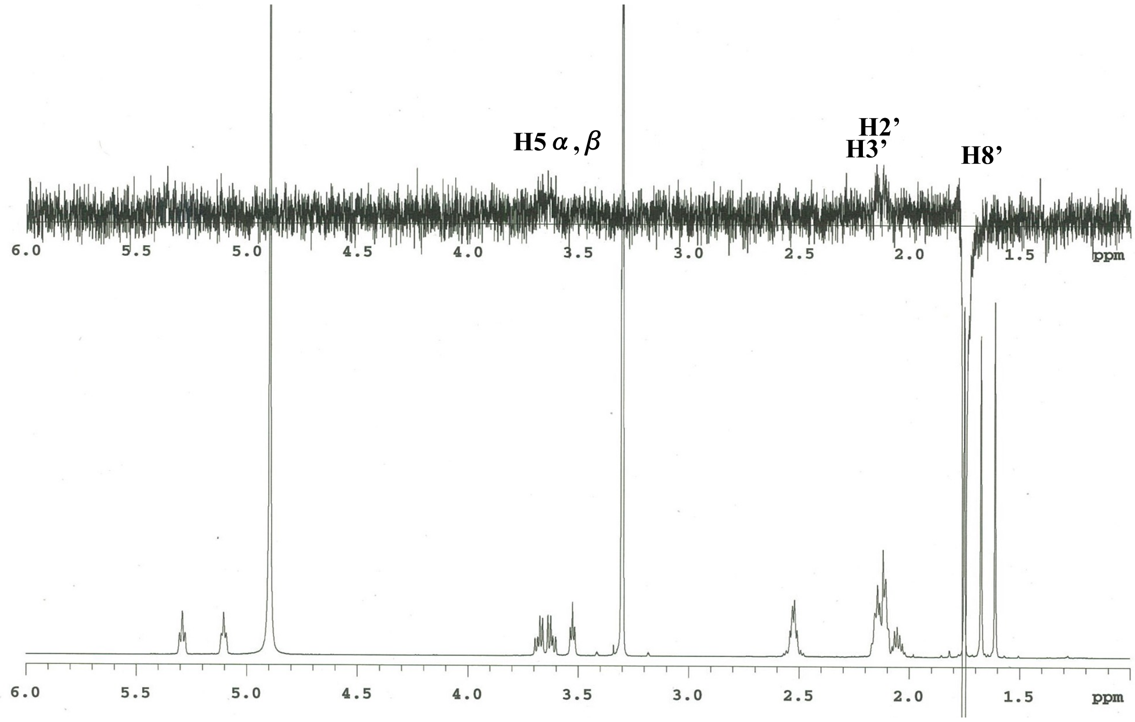

Figure S89. NOESY1D spectrum of synthetic **4** (CD_3OD, 600 MHz). Irradiated at δ1.75 ppm (H8’).

・NMR spectra of synthetic *N*-geranyl-3(*R*)-hydroxy-L-glutamic acid (**7**).

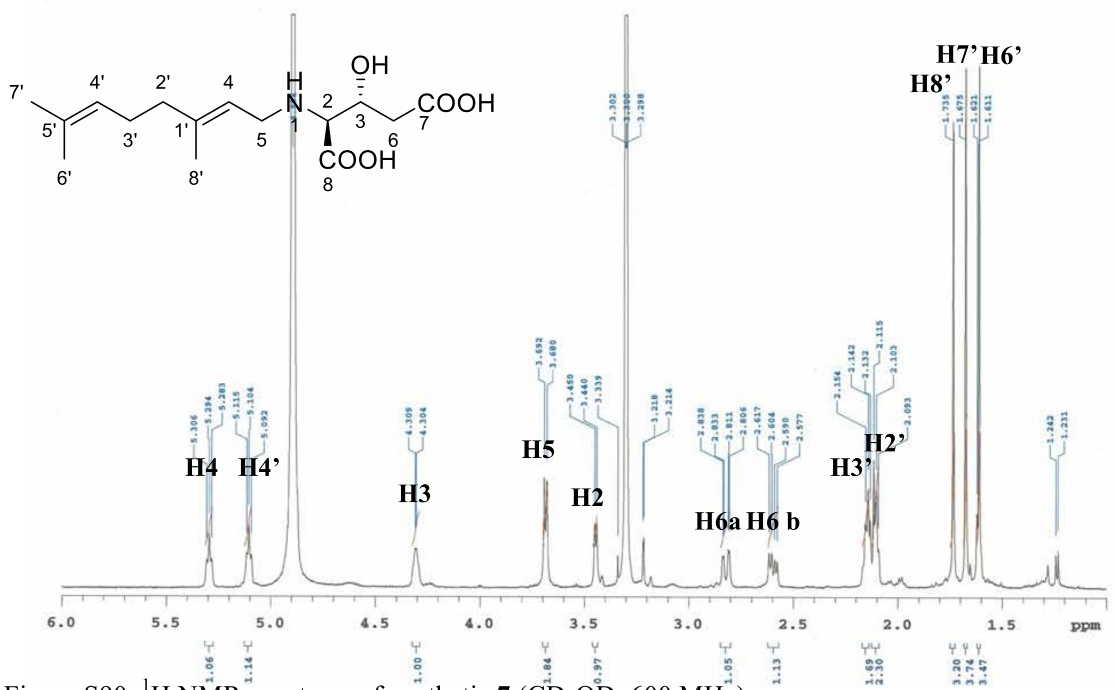

Figure S90. ^{1}H NMR spectrum of synthetic **7** (CD_3OD, 600 MHz).

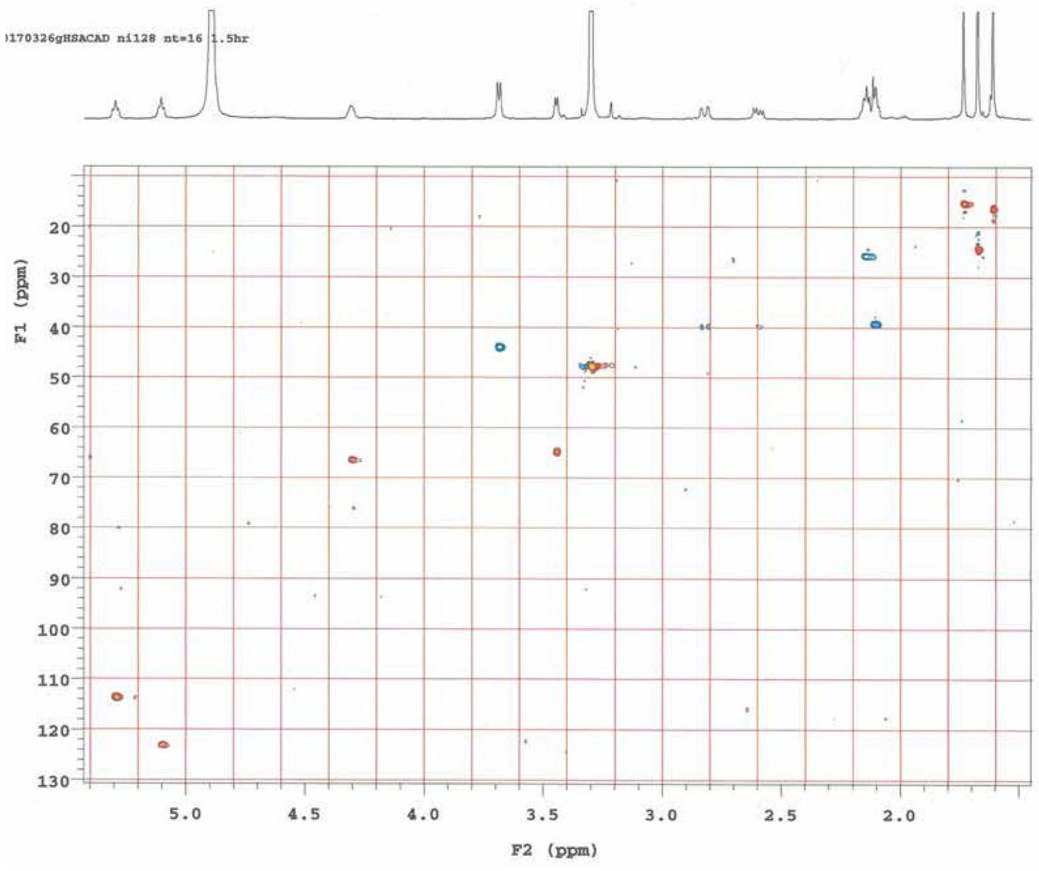

Figure S91. Gradient HSQC spectrum of synthetic **7** (CD_3OD, 600 MHz).

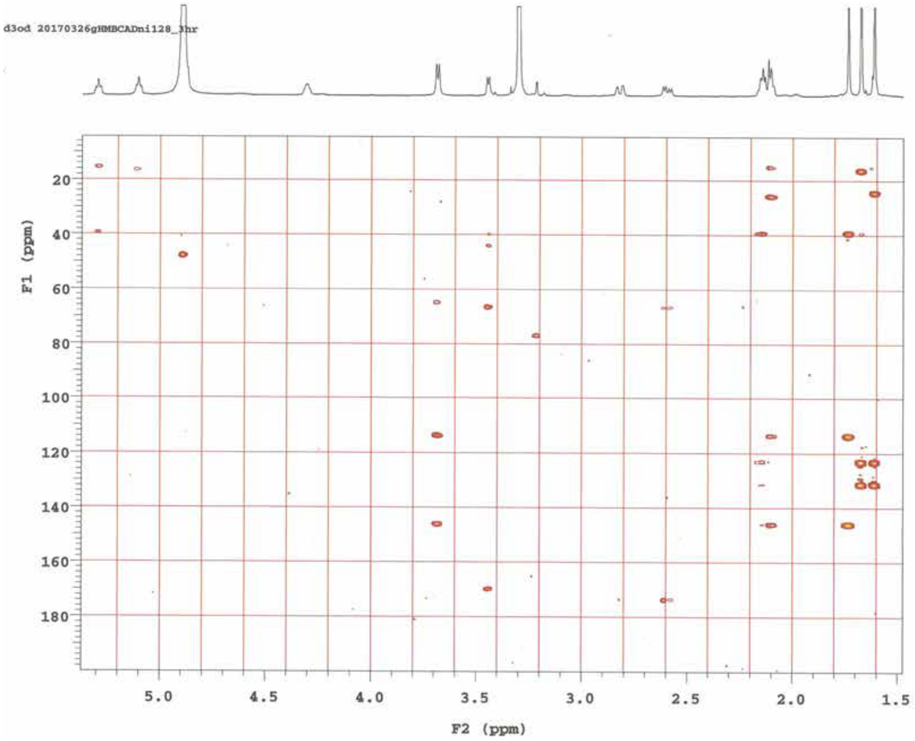

Figure S92. Gradient HMBC spectrum of synthetic **7** (CD_3OD, 600 MHz).

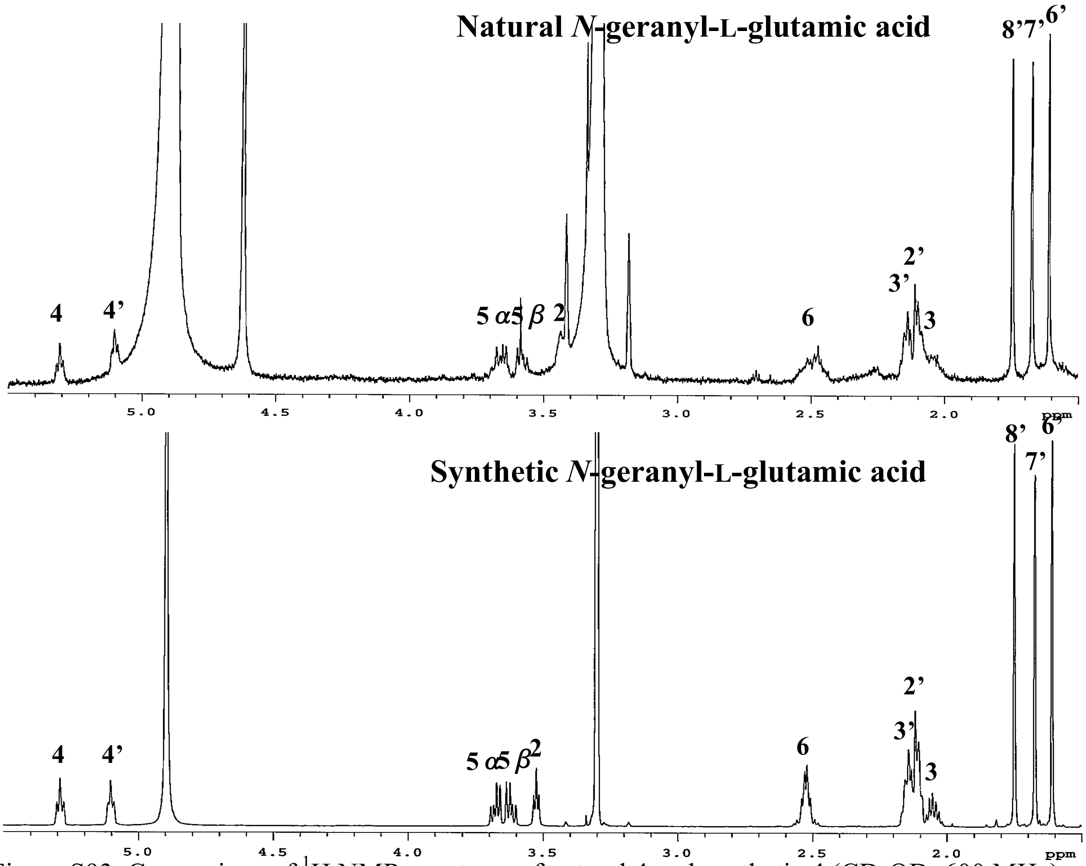

Figure S93. Comparison of ^{1}H NMR spectrum of natural **4** and synthetic **4** (CD_3OD, 600 MHz).

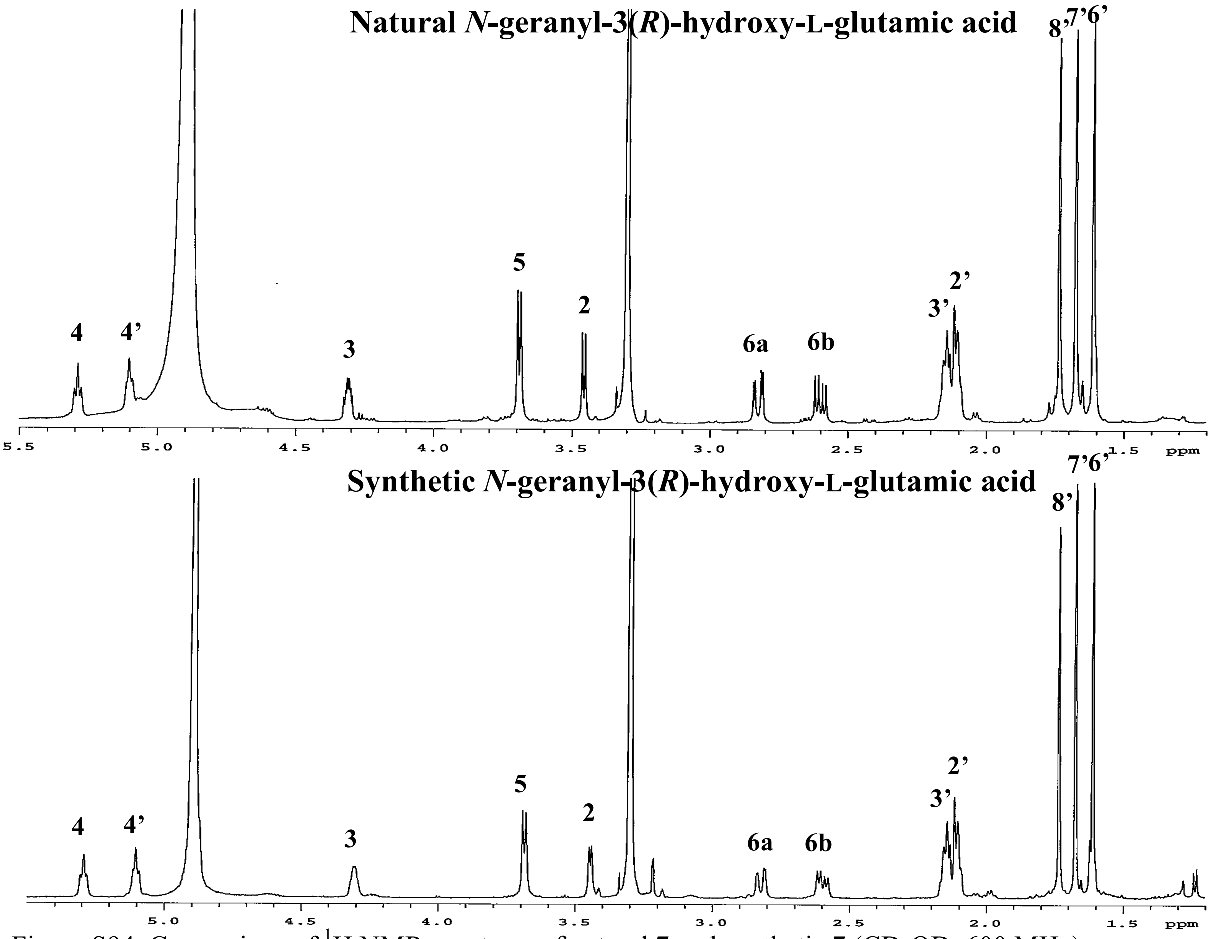

Figure S94. Comparison of ^{1}H NMR spectrum of natural **7** and synthetic **7** (CD_3OD, 600 MHz).

・NMR data of synthetic **4** and **7**.

Synthetic *N*-geranyl-L-glutamic acid (**4**): NMR data.

^{1}H NMR (CD_3OD, 600 MHz)δ 5.29 (1H, t, *J* = 7.3 Hz, H-4), 5.10 (1H, t, *J* = 6.5 Hz, H-4'), 3.67 (1H, dd, *J* = 13.4, 7.3 Hz, H-5α), 3.62 (1H, dd, *J* = 13.9, 7.9 Hz, H-5β), 3.53 (1H, t, *J* = 5.9 Hz, H-2), 2.53 (2H, m, H-6), 2.14 (2H, dt, *J* = 13.2, 7.1 Hz, H-3'), 2.11 (2H, t, *J* = 7.0 H-2'), 2.05 (2H, m, H-3), 1.75 (3H, s, H-8'), 1.67 (3H, s, H-7'), 1.61 (3H, s, H-6'); ^{13}C-NMR (CD_3OD, 151 MHz) δ 177.7 (COOH, C-7), 173.6 (COOH, C-8), 148.3 (C, C-1'), 133.8 (C, C-5'), 125.5 (CH, C-4'), 115.9 (CH, C-4), 62.7 (CH, C-2), 46.1 (CH_2, C-5), 41.5 (CH_2, C-2'), 32.2 (CH_2, C-6), 28.0 (CH_2, C-3'), 27.4 (CH_2, C-3), 26.7 (CH_3, C-7'), 18.6 (CH_3, C-6'), 17.5 (CH_3, C-8').

Synthetic *N*-geranyl-3(*R*)-hydroxy-L-glutamic acid (**7**): NMR data.

^{1}H NMR (CD_3OD, 600 MHz)δ 5.29 (1H, t, *J* = 7.3 Hz, H-4), 5.10 (1H, t, *J* = 6.8 Hz, H-4'), 4.31 (1H, d, *J* = 2.9 Hz, H-3), 3.69 (2H, d, *J* = 7.3 Hz, H-5), 3.45 (1H, d, *J* = 5.9 Hz, H-2), 2.82 (1H, dd, *J* = 16.0, 3.2 Hz, H-6a), 2.60 (1H, dd, *J* = 16.0, 7.6 Hz, H-6b), 2.14 (2H, q, *J* = 7.0 Hz, H-3'), 2.11 (2H, t, 7.0 H-2'), 1.74 (3H, s, H-8'), 1.68 (3H, s, H-7'), 1.61 (3H, s, H-6'); ^{13}C-NMR (CD_3OD, 151 MHz) δ 175.4 (COOH, C-7), 171.5 (COOH, C-8), 148.0 (C, C-1'), 133.1 (C, C-5'), 124.7 (CH, C-4'), 115.4 (CH, C-4), 68.3 (CH, C-3), 66.6 (CH, C-2), 45.8 (CH_2, C-5), 41.3 (CH_2, C-6), 40.9 (CH_2, C-2'), 27.3 (CH_2, C-3'), 26.1 (CH_3, C-7'), 17.9 (CH_3, C-6'), 17.0 (CH_3, C-8').

4. HR-LC-MS/MS spectra of natural 4 and synthetic 4.

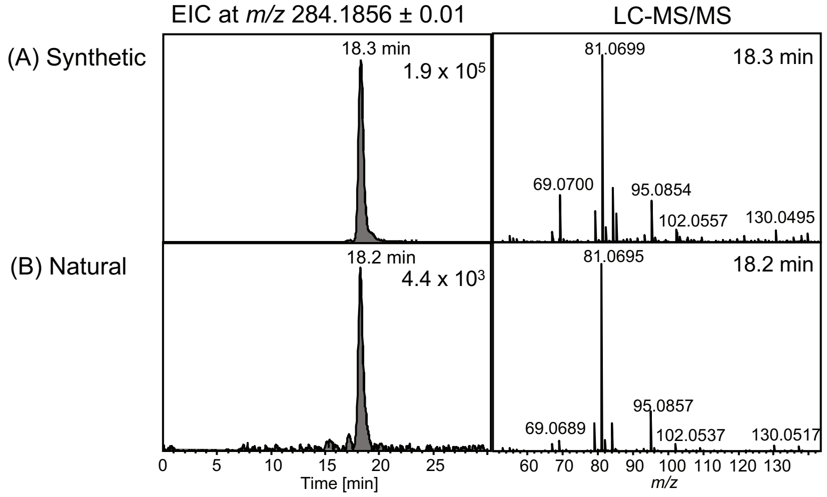

Figure S95. HR LC-MS/MS spectra of 284 (**4**). (A) Synthetic **4** and (B) natural **4** semi-purified from *C. armata*. The LC-MS conditions: Mightysil RP-18GP (5 µm, 0.2 x 15 cm), H_2O-MeOH-HCOOH (60:40:0.1, v/v/v), 0.2 mL/min. The MS/MS spectra were measured in the auto MS/MS mode. The precursor ions were of *m/z* 284.19 and width 2 Da. The sweeping collision energy was 40–160 eV. The amounts of the synthetic and natural **4** were 22 and 0.53 ng, respectively.

5. HR-ESI-MS spectra and NMR spectra of [^{15}N, D]*N*-geranyl-L-glutamic acid (4').

▪HR-ESI-MS spectra of [^{15}N, D]*N*-geranyl-L-glutamic acid (**4'**)

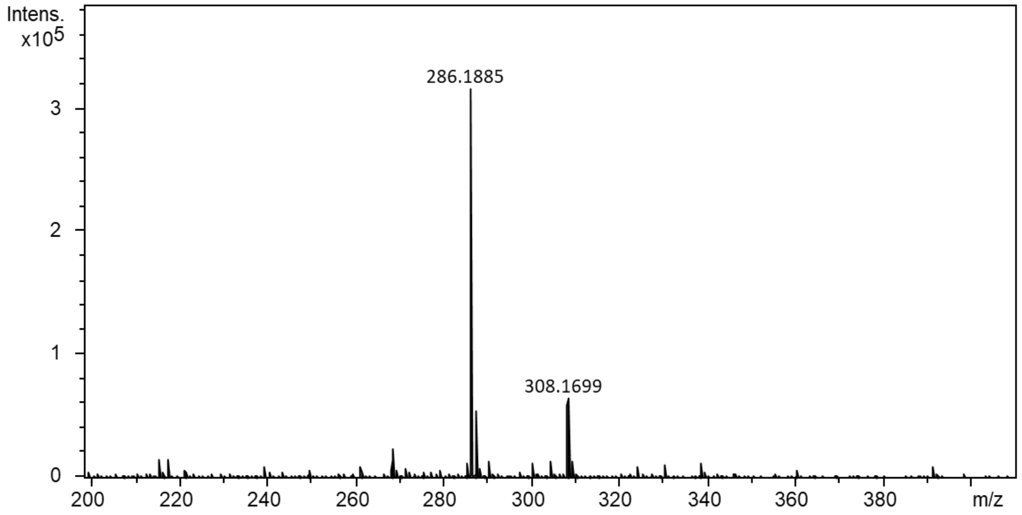

Figure S96. HR-ESI-TOF mass spectrum of [^{15}N, D]*N*-geranyl-L-glutamic acid (**4'**)

▪NMR spectra of [^{15}N, D]*N*-geranyl-L-glutamic acid (**4'**)

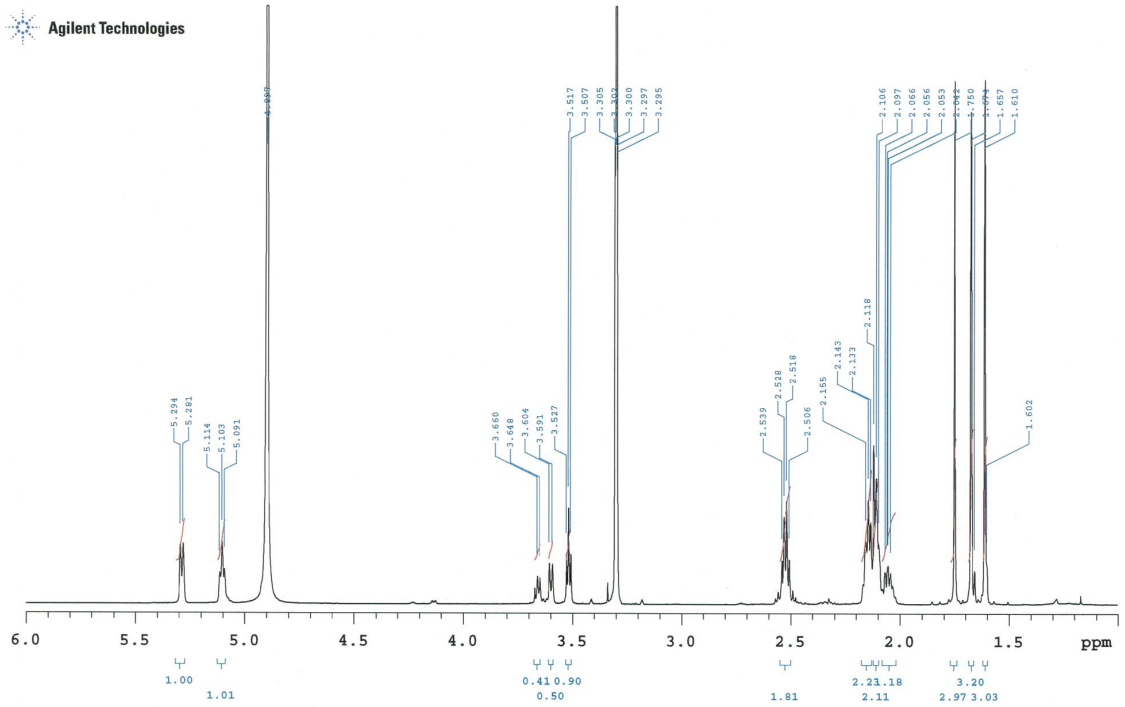

Figure S97. ^{1}H NMR spectrum of [^{15}N, D]*N*-geranyl-L-glutamic acid (**4'**) (CD_3OD, 600 MHz).

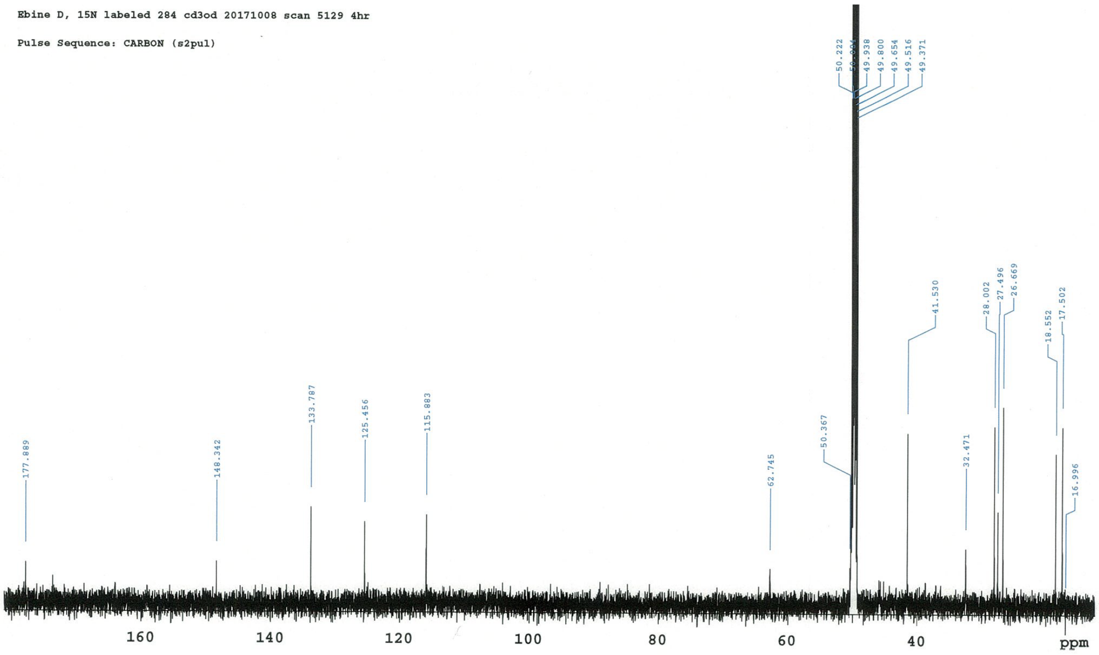

Figure S98. ^{13}C NMR spectrum of [^{15}N, D]*N*-geranyl-L-glutamic acid (**4’**) (CD_3OD, 600 MHz).

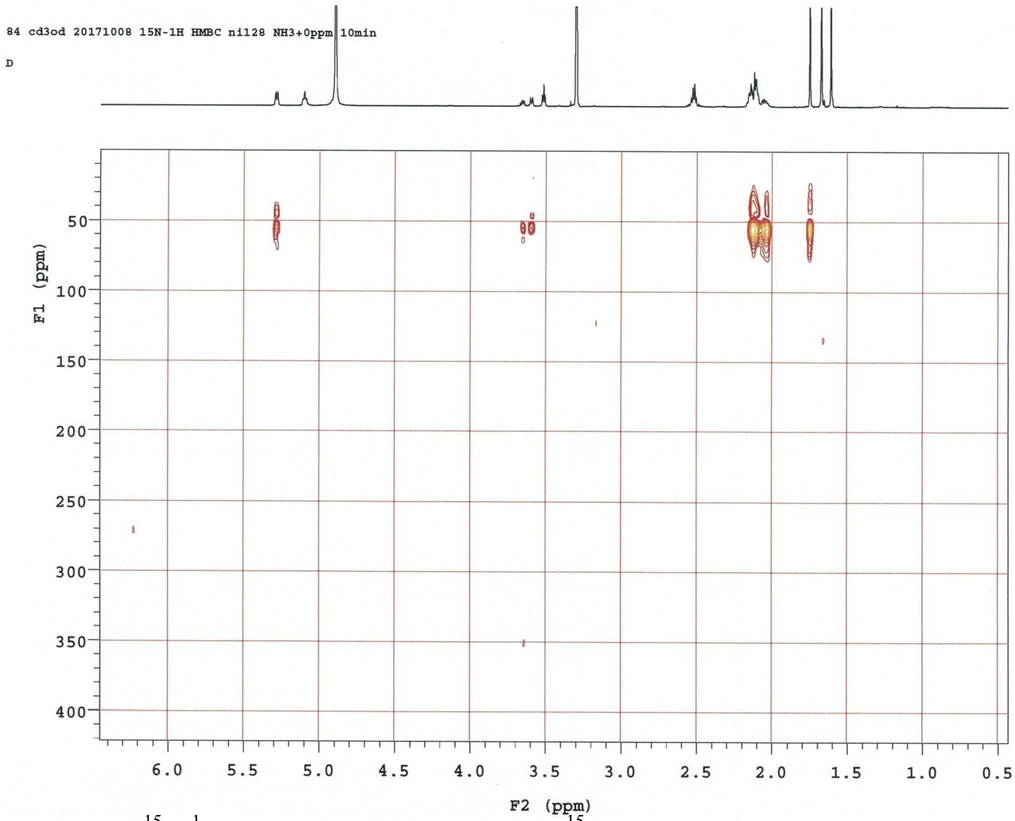

Figure S99. ^{15}N-^{1}H gradient HMBC spectrum of [^{15}N, D]*N*-geranyl-L-glutamic acid (**4’**) (CD_3OD, 600 MHz, $^{15}NH_3$=0 ppm)

6. Synthesis and purification of 9.

Threo-3-hydroxy glutamic acid (racemic; **9**) was synthesized from dimethyl 3-oxoglutarate (100 mg, 0.575 mmol) according to the reported method,[1] and obtained as a crude mixture of *erythro* and *threo* diastereomers (**9'**, 2 mg, 0.012 mmol). Before reaction, **9** was purified as follows. The solution of crude **9'** in 6 M HCl (1.5 mL) was passed through a Cosmosil 140C_{18}OPN column (1.5 mL) and **9'** was further washed with water (4.5 mL). These passing solution and water fraction were mixed and diluted with water to adjust the concentration of HCl to 0.1 M, and then loaded to a Dowex 50WX8 column (200–400 mesh, 1 mL; Muromachi Chemicals Inc., Fukuoka, Japan). The column was washed with 1.5 mL of water, and then, **9'** was eluted with 5 mL of 1 M NH_4OH. After removal of the solvent under vacuum, **9'** was dissolved with H_2O-HCOOH (100:0.1, v/v, 50 µl), and loaded into a TCI Dual ODS-CX15 column (4.6 × 250 mm, 5 µm, Tokyo Chemical Industries) pre-equilibrated with H_2O-HCOOH (100:0.1, v/v) to separate *threo* and *erythro* diastereomers. Compound **9** was eluted faster than its *erythro* type. The stereochemistry of obtained **9** (1 mg, 0.0061 mmol) was identified by ^1H NMR by comparison with reported data.[1,2]

Threo-3-hydroxy glutamic acid (**9**): HRESIMS $[M+H]^+$ *m/z* 164.0553 (calcd for $C_5H_{10}NO_5^+$ 164.0553). ^1H NMR (D_2O, 600 MHz) δ 4.44 (1H, dt, J = 8.4, 4.4 Hz, H3), δ 3.68 (1H, d, J = 4.4 Hz, H-2), δ 2.66 (1H, dd, J = 4.1, 15.9 Hz, H-4a), δ 2.53 (1H, dd, J = 8.5, 15.7 Hz, H-4b); ^{13}C-NMR (D_2O, 600 MHz,) δ 176.68 (COOH, C-5), 172.3 (COOH, C-1), 66.7 (CH, C-3), 58.6 (CH, C-2), 40.5 (CH_2, C-4).

7. LC-MS for routine detection of 1–7.

LC-MS was recorded on an API2000 mass spectrometer (ESI). A Mightysil RP-18GP column (2.0 × 150 mm, 5 µm) was used with H_2O-MeOH-HCOOH (40:60:0.1, v/v/v) for **2**, **3**, **4**, and **7**, and H_2O-MeOH-HCOOH (70:30:0.1, v/v/v) for **5** and **6** as the mobile phase at a flow rate of 0.2 mL/min at 25°C. Five ions at *m/z* 282, 284, 298, 300, and 312 corresponding to the $[M+H]^+$ ions of **1**–**7** were detected in the single ion monitoring (SIM) mode.

References

1. Broberg, A., Menkis, A. & Vasiliauskas, R. Kutznerides 1－4, Depsipeptides from the actinomycete *Kutzneria* sp. 744 inhabiting mycorrhizal roots of *Picea abies* seedlings. *J. Nat. Prod*. **69**, 97–102 (2006).
2. Tamborini, L. *et al*. Synthesis of new β-and γ-benzyloxy-*S*-glutamic acid derivatives and evaluation of their activity as inhibitors of excitatory amino acid transporters. *Tetrahedron* **65**, 6083–6089 (2009).
